# Integrating clinical decision support and mobile health for differentiated HIV service delivery in Lesotho (VITAL): a cluster-randomised non-inferiority trial

**DOI:** 10.1016/j.eclinm.2026.103850

**Published:** 2026-04-02

**Authors:** Nadine Tschumi, Malebanye Lerotholi, Mathebe Kopo, Mpho Kao, Lipontso Motaboli, Moleboheng Mokebe, Ntoiseng Chejane, Makobefo Chakela, Bienvenu L. Nsakala, Blaise Lukau, Alastair van Heerden, Ruanne V. Barnabas, Jesse Heitner, Adrienne E. Shapiro, Lorena Urda, Giuliana Sanchez, Tristan Lee, Jennifer A. Brown, Alain Amstutz, Jennifer M. Belus, Frédérique Chammartin, Niklaus D. Labhardt

**Affiliations:** a Division of Clinical Epidemiology, Department of Clinical Research, University Hospital Basel, Basel, Switzerland; b University of Basel, Basel, Switzerland; c SolidarMed Partnerships for Health, Maseru, Lesotho; d Syndicate for Public Science and Emerging Technologies, Wits Health Consortium, South Africa; e SAMRC/WITS Developmental Pathways for Health Research Unit, Department of Paediatrics, School of Clinical Medicine, University of the Witwatersrand, Johannesburg, Gauteng, South Africa; f Division of Infectious Diseases, Massachusetts General Hospital, Boston, MA, USA; g Departments of Global Health and Medicine, University of Washington, Seattle, USA; h Population Health Sciences, Bristol Medical School, University of Bristol, United Kingdom; i Oslo Centre for Biostatistics and Epidemiology, Oslo University Hospital, University of Oslo, Norway

**Keywords:** HIV, Antiretroviral therapy, Digital health, Differentiated service delivery, Africa

## Abstract

**Background:**

The World Health Organization conditionally endorses digital interventions to strengthen health systems, while acknowledging limited evidence on their benefits and harms. We assessed the effectiveness of digital health-supported differentiated service delivery on HIV treatment outcomes in rural southern Africa.

**Methods:**

The VITAL pragmatic, open-label, parallel-group, non-inferiority, cluster-randomised controlled clinical trial enrolled adults with HIV taking antiretroviral therapy (ART) at 18 rural, nurse-led clinics in Lesotho. Clinics were randomised to receive either digital documentation and clinical decision support for providers, along with mobile health for participants–comprising individualised automated text messaging and telemedicine–and preference-based multi-month dispensing of ART (VITAL group), or only digital documentation for providers (enhanced standard of care [eSOC] group). The primary endpoint was engagement in care with documented viral suppression (<50 copies per mL) at 24 months (window: 16–28 months). The adjusted odds ratio (aOR) was estimated in the modified intention-to-treat (mITT) population with a non-inferiority margin of 0.8. Safety endpoints were all-cause mortality, tuberculosis diagnoses, and disengagement from care. The trial was registered with ClinicalTrials.gov (NCT04527874) and the trial status is completed.

**Findings:**

Between October 14, 2020 and March 30, 2022, 5809 participants were enrolled, of whom 5770 were included in the mITT analysis (3401 in the VITAL group and 2369 in the eSOC group). The primary endpoint was reached by 2649 (77.9%) in the VITAL group and by 1759 (74.3%) in the eSOC group (aOR 1.18 [95% CI 0.95–1.46]). All-cause mortality and tuberculosis diagnosis were similar between groups (80 [2.4%] in the VITAL group and 53 [2.2%] in the eSOC group, adjusted hazard ratio 1.10 [0.78 to 1.58]; 15 [0.4%] in the VITAL group and 14 [0.6%] in the eSOC group, aOR 0.70 [0.30–1.63]). Disengagement from care was lower in the VITAL group (156 [4.6%] in the VITAL group and 167 [7.1%] in the eSOC group; aOR 0.67 [0.48–0.93]).

**Interpretation:**

Digital health-supported differentiated service delivery maintained levels of viral suppression and engagement in routine rural HIV care without increasing adverse outcomes. Although superiority was not demonstrated, the findings support the safe integration of digital health tools and multi-month dispensing of ART into HIV care. Mid-trial changes in national ART guidelines may have attenuated differences between groups.

**Funding:**

Moritz Straus-Foundation, 10.13039/100000001Swiss National Science Foundation.

**Research in context**

**Evidence before this study**

We searched PubMed on May 8, 2025, with the search strategy shown in Supplement Table S1, for randomised controlled trials, reviews and meta-analyses published between January 2018 and May 2025 on digital health-supported HIV care in Africa. A recent Cochrane review found that combining digital documentation with clinical decision support and mobile health interventions (e.g., appointment or adherence reminders) may improve healthcare processes, such as adherence to clinical guidelines, but provided limited evidence of improvements in clinical outcomes. Randomised controlled trials and meta-analyses on digital health interventions in Africa remain scarce and limited in scope, focusing primarily on text message reminders for appointments or medication adherence, and report inconsistent results regarding their effectiveness. One large multinational trial found no benefit of two-way text messaging on viral suppression among people with HIV experiencing second-line treatment failure. Only one randomised trial—PEBRA–specifically evaluated digital health-supported differentiated service delivery among adolescents and young adults with HIV in Lesotho; however, it did not demonstrate a significant improvement in viral suppression.

**Added value of this study**

To our knowledge, VITAL is the first randomised trial to evaluate a digital health-supported differentiated service delivery model applicable to all adults in HIV care in Africa. Unlike previous interventions that focused on either participants or providers, VITAL integrated provider-facing digital documentation and clinical decision support with participant-facing mobile health—comprising individualised automated text messaging and telemedicine–and preference-based multi-month ART dispensing. Conducted at 18 rural clinics in Lesotho, southern Africa, this pragmatic cluster-randomised trial enrolled 5770 participants and implemented digital health interventions built upon routine viral load monitoring data. The VITAL intervention was non-inferior but not superior to the enhanced standard of care in terms of the proportion of participants engaged in care with viral suppression at 24 months and showed modest improvement of engagement in care.

**Implications of all the available evidence**

Taken together with previous evidence, the non-inferiority of the VITAL trial suggests that broad implementation of digital health-supported differentiated service delivery in southern Africa depends on the cost of digital health solutions, user acceptability, and infrastructure readiness. The absence of superiority in clinical outcomes underscores the limitations of digital interventions, even when targeted at both providers and clients, in the absence of other systemic enablers–such as integrated electronic medical records, a digitally literate health workforce, and supportive national policies. In the context of constrained global HIV funding, simpler differentiated service delivery models may offer a more efficient use of resources. Nonetheless, future trials should reassess the cost-effectiveness and clinical value of digital health-supported differentiated service delivery as smartphone access, digital literacy, and national electronic medical records expand in rural southern Africa.

## Introduction

Over the past decades, the number of people receiving antiretroviral therapy (ART) globally has risen to over 30 million, representing an extraordinary achievement in the global HIV response.^1^ However, this expansion places growing strain on health systems, particularly high-burden settings where shortages of skilled healthcare workers and widening funding gaps present major challenges to sustaining quality care.^2^ The recent large-scale, unexpected United States funding cuts have further exposed the vulnerability of HIV programmes, underscoring the need for more resilient and efficient models of care.^3^

Differentiated service delivery has emerged as a strategy to improve the efficiency of HIV programmes while advancing client-centred care.^4^ A key component of differentiated service delivery is multi-month dispensing (MMD) of ART for people with viral suppression. In a pragmatic randomised trial in southern Africa, six-month MMD was found to be non-inferior to standard of care in terms of engagement in care.^5^ During the COVID-19 pandemic, the adoption of MMD was accelerated as a strategy to limit clinic visits and maintain continuity of care amid lockdowns and service disruptions.^6^

While MMD can ease the burden on health facilities, reduce travel costs, and minimize waiting times for clients,^7^^,^^8^ complementary mobile health offers potential to strengthen client–provider relationships, improve information exchange, encourage treatment adherence, and enhance quality of care.^9^ Over the past two decades, the rapid expansion in mobile phone coverage, affordability, and functionality in low-resource settings has created new opportunities for wide-reaching mobile health and telemedicine initiatives. Meta-analyses of mobile health interventions in Africa remain limited in scope, focusing primarily on client appointment or medication adherence reminders, and report inconsistent results regarding their effectiveness.^10^, ^11^, ^12^

Concurrently, the emerging digitalisation of health systems in low- and middle-income countries (LMICs) is creating new opportunities for more sophisticated digital interventions. Among these, digital documentation and clinical decision support systems hold promise for improving quality of care.^13^ Digital documentation systems help healthcare workers to collect and store client data, enabling them to track data more accurately and securely compared to paper-based systems, which are still widely used in LMICs. Based on the client data available in digital documentation systems, clinical decision support systems guide real-time clinical decision-making.^13^ A 2025 Cochrane review found that clinical decision support systems may improve healthcare processes such as guideline adherence. However, effects on clinical endpoints (e.g., mortality, disease control, or engagement in care) were variable and generally modest, with the overall strength of evidence limited by the small number and low quality of included studies.^14^ Similarly, the World Health Organization (WHO) recognizes the potential of digital decision support tools for frontline healthcare providers but notes a “limited evidence base” supporting their clinical impact in LMICs, especially for complex chronic diseases like HIV.^15^

To address this evidence gap, we conducted a cluster-randomised trial in rural Lesotho to assess the effectiveness of a digital health-supported differentiated HIV service delivery model that combines clinical decision support for providers with mobile health support and preference-based multi-month ART distribution for people with HIV.

## Methods

### Study design

The *Viral load triggered ART care in Lesotho (VITAL)* pragmatic, open-label, parallel-group, non-inferiority, cluster-randomised controlled clinical trial assessed 24-months outcomes among people taking ART. The intervention group received a digital health-supported differentiated HIV service delivery model that combined provider-facing clinical decision support and digital documentation with client-facing mobile health—comprising individualised automated text messaging and telemedicine–and preference-based multi-month ART dispensing (VITAL group). The control group received provider-facing digital documentation (enhanced standard of care [eSOC] group). A non-inferiority design was chosen because the intervention introduced longer ART dispensing intervals, which were untested at the time of protocol development. At the same time, its digital components were expected to improve information exchange, support nurse-led clinical decision-making, and empower clients via mobile health, thereby enhancing adherence to clinical protocols and maintaining quality of care despite prolonged visit intervals.

The VITAL trial was conducted at eighteen clinics in Butha-Buthe and Mokhotlong districts of Lesotho in southern Africa (Supplement Table S2). All included clinics were rurally located, nurse-led, and offered outpatient primary care services, including HIV care. The trial was approved in Lesotho by the National Health Research Ethics Committee (ID 220-2019). Additionally, the Ethikkommission Nordwest und Zentralschweiz in Switzerland provided a statement confirming the trial meets ethical requirements for a Swiss research project (Req-2020-00067). A study protocol manuscript has been published previously.^16^ The trial was registered on August 27, 2020 with ClinicalTrials.gov (NCT04527874), where a statistical analysis plan is available.

### Participants

Clinic was the unit of randomisation. Eligible clinics were public or missionary nurse-led clinics (not hospitals) providing ART services, situated in an area with regular internet connection, and with consent of the clinic management. People with HIV were eligible for inclusion if they were receiving ART, were above eighteen years old, expressed the intention to remain at the same clinic for the duration of the trial, and provided written informed consent as described previously.^16^ Access to a mobile phone was not an eligibility criterion. The enrolment period of at least six months was selected to ensure that all individuals receiving HIV care at participating clinics had the opportunity to be offered participation. Data on sex were retrieved from medical records or if not available at enrolment, through self-report with the options of female or male (matching the available options in local medical records).

### Randomisation and masking

Clinics were randomly assigned via concealed 1:1 allocation to either the VITAL or the eSOC group. Randomisation was stratified by district and took place at meetings with representatives of all eligible clinics in both districts. At these events, clinic representatives drew opaque, sealed, equally sized envelopes containing the group allocation. Allocation disclosure and documentation thereof only happened after all representatives had drawn their envelope. The sequence of drawing was randomly generated in advance by an independent person drawing from a second pile of opaque, sealed envelopes containing the names of the clinics. Participants, clinic staff and study staff were not masked due to the nature of the intervention.

### VITAL application

The VITAL application (VITALapp) was custom-built as a clinical documentation tool, a clinical decision support system, and an interface for automated text-message provision to participants. The VITALapp was linked to the Viral Load Cohort North-East Lesotho (VICONEL), which has been collecting all routine viral load measurements in Butha-Buthe and Mokhotlong districts from the laboratory information system since 2016 and 2018, resepctively.^17^ Through this connection, the VITALapp accessed and displayed participants’ clinical and laboratory history, including past viral load results and current as well as previous ART regimens. Aligned with the National HIV Management Guidelines, the VITALapp then recommended scheduling of viral load testing, management of unsuppressed viral loads, virologic failure, and VITAL trial-specific procedures such as MMD of ART.^18^^,^^19^ The VITALapp recommendations were updated during the trial in December 2022 to align with an amendment to the national ART guidelines.^19^

Through the VITALapp, text messages to participants could be customised by providers or study staff and were then fed back to the VICONEL database, from which the personalised messages were automatically dispatched at predefined time points. The VITALapp was used by healthcare providers throughout the study period for all consultations of VITAL participants across both groups. However, in control clinics, the VITALapp only allowed for documentation and displaying participants’ clinical and laboratory history from the VICONEL database (see Supplement Section A for more details).

### Intervention and control

At the VITAL clinics, providers received clinical decision support through the VITALapp and participants received preference-based mobile health support. During the enrolment visit, participants could opt into automated text messaging and customize their choice. Available text message options were: viral load result notification, clinic visit reminders, and ART adherence reminders. Participants had two telemedical support options: (1) they could request a call-back at any time for a telemedical consultation with a VITAL study nurse regarding any clinical or study-related questions; and (2) in cases of viraemia, they could choose between standard-of-care enhanced adherence counselling at the clinic or telemedical adherence counselling. Additionally, at the enrolment visit, participants completed a questionnaire to indicate their preferred duration for ART MMD (ranging from 1 to 12 months).

For providers, in addition to displaying relevant clinical and laboratory information, the VITALapp provided clinical decision support for HIV care. Depending on the last available previous viral load result it recommended the next date for guideline-adherent viral load testing. In cases of a suppressed viral load, the VITALapp recommended MMD until the next scheduled test while also providing the participant's documented MMD preference. In case of an unsuppressed viral load, the VITALapp provided the respective recommendations for a timely follow-up viral load testing and asked the nurse to offer participants the choice between onsite or telemedical enhanced adherence counselling. The VITALapp featured an automatically updated list of participants with virological failure and included a streamlined digital procedure to notify the district ART advisory committee to start the process of switch to second-line ART regimen for those eligible (see Supplement Section B for more details). Further, the VITALapp provided guidance on tuberculosis (TB) diagnostic testing for participants reporting TB symptoms and provision of TB preventive therapy for those eligible. All recommendations followed the National Guidelines that were in place at the time.^18^^,^^19^ The VITAL intervention was developed and refined with input from local healthcare professionals, staff from participating facilities, district-level stakeholders, and community members. Across both groups, the VITALapp was used for clinical documentation purposes, including displaying each participant's last three viral load results if available. Additionally, study staff in both groups received, via the VITALapp, a list of participants who were overdue for their next clinic visit by at least two months, along with their contact information, to facilitate and document tracing. However, in the eSOC group, all other functionalities of the VITALapp–namely clinical decision support, the list of participants with treatment failure, and the mobile health interface–were disabled.

### Procedures

At the enrolment visit, consenting participants attending routine HIV care were enrolled and interviewed by study staff following a structured questionnaire on the VITALapp before completing routine clinical consultation with a clinic nurse. Apart from optional telemedical consultations in the VITAL group, the study staff had no further interaction with participants after the enrolment visit. All care and follow-up decisions were made by routine clinic staff. Nurses could overrule recommendations from the VITALapp at any time without providing a justification. There were no protocol-defined visits or viral load testing; instead, individual viral load monitoring schedules as per national guidelines were followed.^18^^,^^19^

Individuals did not receive financial incentives for participating in the study, but participating clinics in both groups were remunerated ($0.80 per enrolled participant and $0.80 for each complete 12-month follow-up).

Serious adverse events were systematically captured (see Supplement Section C). Serious adverse events were defined as any untoward medical occurrence that resulted in death, was life-threatening, required hospitalisation, led to persistent or significant disability or incapacity, or caused a congenital anomaly or birth defect. Each serious adverse event was assessed by the sponsor-investigator to determine its potential relationship to the study intervention.

Time and motion studies tracking clinic staff activities during participant visits were conducted on a convenience sample of enrolment and follow-up visits to explore potential differences in staff resource requirements resulting from the intervention. Additionally, workload assessment surveys were administered to a convenience sample of clinic staff to gather insights into the effort required to implement activities within the VITAL and eSOC groups.

### Outcomes

The primary endpoint was engagement in care with documented viral suppression (<50 copies per mL) at 24 months (window 16–28 months). The primary endpoint was ascertained through routine care and centrally assessed using the VICONEL database.^17^ Onsite documentation checks were performed for all participants without a viral load result within the endpoint window. The absence of a viral load measurement for any reason within the primary endpoint window–which, according to national guidelines, all participants should have undergone^18^^,^^19^–was considered a failure to reach the primary endpoint. The cutoff for viral suppression in the primary endpoint was amended from 20 copies per mL to 50 copies per mL during the trial to align with the January 2022 amendment to the national ART guidelines, which revised the threshold defining viraemia from 1000 to 50 copies per mL, and considering the detection threshold of 40 copies per mL of the increasingly used point-of-care viral load testing at some of the study-sites.^19^ The first primary endpoint window opened thereafter in February 2022, and all participants were assessed using this updated cutoff. All viral load and ART regimen data used for secondary analyses were obtained from routine care records and centrally assessed via the VICONEL database. VITAL visit data and TB preventive therapy data were collected through the VITALapp, while TB diagnosis data–including clinical diagnoses and results from GeneXpert, TB Lipoarabinomannan (LAM), chest X-ray, culture and drug susceptibility testing, and line probe assay–were extracted from paper-based clinic records.

Secondary endpoints were: (1) the primary endpoint among participants with an unsuppressed viral load (≥50 copies per mL) during the first 12 months (window 0–16 months) of follow-up; (2) mortality at 12 (window 0–12 months) and 24 months (window 0–28 months); (3) TB diagnosis at 12 (window 0–16 months) and 24 months (window 0–28 months); (4) disengagement from care at 12 (window 4–16 months) and 24 months (window 16–28 months) defined as the absence of both a documented clinic visit and a viral load test; (5) time to follow-up viral load in case of an unsuppressed viral load (≥50 copies per mL); (6) time to ART regimen adaption in case of virologic failure; (7) rate of clinic visits at 24 months after enrolment (i.e., average number of visits per person-year); (8) ART regimen modification due to virologic failure at 12 (window 0–16 months) and 24 months (window 0–28 months) among participants with virologic failure; and (9) having ever received TB preventive therapy by 24 months (window 0–28 months). All amendments to protocol-defined endpoints–including secondary endpoints moved to separate manuscripts (technical mHealth outcomes and cervical cancer screening), feasibility-related endpoints that were dropped, and the two additional sensitivity analyses–are detailed in the statistical analysis plan section 6.1., and in Supplement Table S3.

Costing analyses assessed provider human resource needs per participant in both groups in terms of median provider time spent per visit.

### Statistical analysis

Based on data from the participating clinics before trial start, we assumed that 65% of participants would have a documented suppressed viral load within the endpoint window. The non-inferiority margin for the lower bound of the 95% confidence interval (CI) of the adjusted odds ratio (aOR) of reaching the primary endpoint was set at 0.8. This corresponds to a 5% lower absolute probability of reaching the primary endpoint in the VITAL group compared to the eSOC group. Given the observed trial data, the non-inferiority margin of 0.8 corresponds to an absolute risk difference of 4.5%.

The sample size calculation was based on an individually randomised design, adjusted by a design effect.^20^ Using data from the participating clinics at the time of protocol development, we estimated an intraclass correlation coefficient of 0.04 and incorporated the estimated mean number of participants per clinic, along with its standard deviation, into our design effect. We set the desired type I error rate at 0.025. With nine clusters in both the VITAL and eSOC groups, the trial had 85% power to detect a one-sided difference of more than 10% in the primary endpoint between the two groups.

Baseline characteristics are reported for the modified intention-to-treat (mITT) population, defined as all participants excluding those retrospectively determined to be ineligible or those with missing core data (e.g., patient identifier or enrolment site). Data are reported by randomised group, with medians and interquartile ranges (IQRs) for continuous variables and counts and percentages for categorical variables. The primary endpoint was analysed using multi-level logistic regression models, including the clinic as a random intercept and group allocation and the randomisation stratification factor (district) as fixed effects. No individual-level baseline covariate adjustment was performed. An individual-level analysis was nevertheless selected because cluster sizes varied substantially (Supplement Table S2). Results are reported as adjusted odds ratios (aORs) with 95% CIs and as adjusted absolute risk differences (aRDs) with 95% CIs. CIs were estimated from 1000 parametric bootstrap replicates and served as the basis for drawing conclusions regarding non-inferiority and superiority. In addition, we report p-values for the aOR, calculated using likelihood ratio tests, in reference to the superiority comparison. For the primary endpoint, non-inferiority and superiority in the mITT population were assessed without statistical multiplicity adjustment, following the closed testing principle.^21^

The per-protocol analysis of the primary endpoint was limited to participants with all viral load results available in the VITALapp within 31 days of phlebotomy during the study period. In sensitivity analyses, the primary endpoint analysis was repeated using an alternative definition of viral suppression (<1000 copies per mL, aligned with the WHO threshold for virological failure), and imputing missing viral load measurements in the primary endpoint window by carrying forward the most recent available viral load obtained within 12 months prior to enrolment; both assessed the robustness of the primary analysis to the endpoint definition. Effect modification of the primary endpoint by age group, sex, and mobile phone access was assessed by incorporating an interaction term between the trial group and the effect modifier. For secondary endpoints, multi-level logistic regression models were used to estimate aORs, applying the same adjustments as for the primary outcome. For mortality at 12 and 24 months, we estimated hazard ratios using Cox proportional hazards models, adjusting only for district and group. In cases of unsuppressed viral load, we estimated the adjusted mean time to follow-up using a multi-level linear regression model, applying the same adjustments as for the primary outcome. The number of clinic visits was analysed using a multi-level Poisson regression model with study duration as an offset, estimating the adjusted incidence rate ratio (aIRR) and applying the same adjustments as for the primary outcome. Secondary endpoints were categorised according to European Medicines Agency guidance into three groups: (i) those that may serve as a basis for additional claims; (ii) safety endpoints; and (iii) supportive evidence. For endpoints in category (i), a hierarchical testing procedure was applied to control for multiplicity.^22^ Missing data in the secondary analyses were left as missing and are indicated in the respective results table using footnotes.

A post-hoc analysis was performed to explore the effect of adjusting the primary analysis for viral load in the year prior to enrolment, age, and sex.

### Role of the funding source

The funders of the study had no role in study design, data collection, data analysis, data interpretation, or writing of the report. Authors had full access to the study data, and NT and NDL share the final responsibility for the decision to submit the manuscript for publication.

## Results

**Fig. 1:**
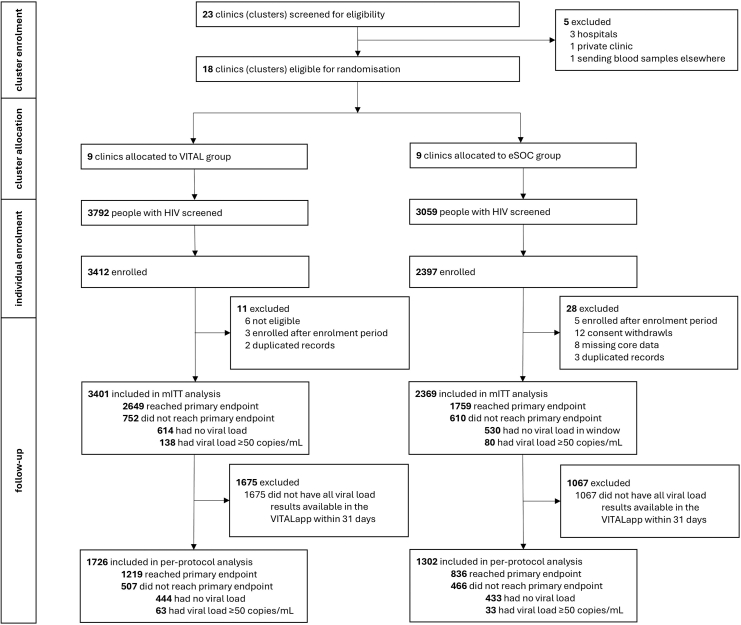
Trial profile. eSOC, enhanced standard of care; mITT, modified intention to treat.

Between October 14, 2020 and March 30, 2022, 6852 individuals were screened and 5809 were enrolled. Thirty-nine participants (11 in the VITAL group and 28 in the eSOC group) were retrospectively found to be ineligible (n = 6), enrolled twice (n = 5), enrolled after the enrolment period (n = 8), withdrew consent during the study (n = 12), or had missing core data (n = 8), resulting in 5770 participants in the mITT population. 3401 (58.9%) were in the VITAL group and 2369 (41.1%) were in eSOC group (Fig. 1).

Baseline sociodemographic and clinical characteristics are summarised in Table 1. 3803 (65.9%) participants were female, the median age was 41.3 years (IQR 33.7–51.4), and the median duration of receiving ART was 5.1 years (IQR 2.7–8.2); these characteristics were similar between groups. There were differences between groups for the stratification factor district (60.8% Butha-Buthe in the eSOC vs. 46.0% in the VITAL group), employment (45.0% unemployed in the eSOC vs. 22.2% in the VITAL group) and the one-way travel time to clinic (22.9% above 120 min in the eSOC vs. 40.1% in the VITAL group).

**Table 1:** Participant characteristics at enrolment.

	VITAL group (n = 3401)	Enhanced standard of care group (n = 2369)	Total (n = 5770)
District			
Butha-Buthe	1566 (46.0%)	1441 (60.8%)	3007 (52.1%)
Mokhotlong	1835 (54.0%)	928 (39.2%)	2763 (47.9%)
Sex			
Female	2256 (66.3%)	1547 (65.3%)	3803 (65.9%)
Male	1145 (33.7%)	822 (34.7%)	1967 (34.1%)
Age	42.1 [34.4, 51.7]	40.4 [32.8, 50.7]	41.3 [33.7, 51.4]
18–24	200 (5.9%)	157 (6.6%)	357 (6.2%)
25–39	1281 (37.7%)	1002 (42.3%)	2283 (39.6%)
40–59	1481 (43.5%)	969 (40.9%)	2450 (42.5%)
≥60	439 (12.9%)	241 (10.2%)	680 (11.8%)
Education (highest level completed)			
No education or less than primary school	670 (19.7%)	308 (13.0%)	978 (16.9%)
Primary school	1451 (42.7%)	1129 (47.7%)	2580 (44.7%)
Secondary or tertiary school	558 (16.4%)	575 (24.3%)	1133 (19.6%)
Other non-formal education	700 (20.6%)	335 (14.1%)	1035 (17.9%)
Missing	22 (0.6%)	22 (0.9%)	44 (0.8%)
Employment situation			
(Self-) employed^a^	2486 (73.1%)	1197 (50.5%)	3683 (63.8%)
Unemployed	755 (22.2%)	1065 (45.0%)	1820 (31.5%)
Other	93 (2.7%)	70 (3.0%)	163 (2.8%)
Missing	67 (2.0%)	37 (1.6%)	104 (1.8%)
One-way travel time to health centre, in minutes			
<60	558 (16.4%)	719 (30.4%)	1277 (22.1%)
60–120	1374 (40.4%)	1084 (45.8%)	2458 (42.6%)
>120	1365 (40.1%)	542 (22.9%)	1907 (33.1%)
Missing	104 (3.1%)	24 (1.0%)	128 (2.2%)
Cost of round-trip transport to health centre, in US$			
<1.1	1035 (30.4%)	730 (30.8%)	1765 (30.6%)
1.1–2.2	1636 (48.1%)	1109 (46.8%)	2745 (47.6%)
>2.2	528 (15.5%)	216 (9.1%)	744 (12.9%)
Missing	202 (5.9%)	314 (13.3%)	516 (8.9%)
Time receiving ART, in years	5.3 [3.0, 8.5]	4.8 [2.3, 7.9]	5.1 [2.7, 8.2]
<5	1570 (46.2%)	1247 (52.6%)	2817 (48.8%)
5–9	1189 (35.0%)	731 (30.9%)	1920 (33.3%)
≥10	642 (18.9%)	391 (16.5%)	1033 (17.9%)
ART regimen at enrolment			
INSTI-based	3316 (97.5%)	2280 (96.2%)	5596 (97.0%)
PI-based	39 (1.1%)	38 (1.6%)	77 (1.3%)
NNRTI-based	42 (1.2%)	46 (1.9%)	88 (1.5%)
Missing	4 (0.1%)	5 (0.2%)	9 (0.2%)
Receiving TB preventive therapy at enrolment			
Yes	95 (2.8%)	104 (4.4%)	199 (3.4%)
No	3306 (97.2%)	2265 (95.6%)	5571 (96.6%)
Receiving TB therapy at enrolment			
Yes	16 (0.5%)	17 (0.7%)	33 (0.6%)
No	3385 (99.5%)	2352 (99.3%)	5737 (99.4%)
Receiving co-trimoxazole at enrolment			
Yes	87 (2.6%)	58 (2.4%)	145 (2.5%)
No	3314 (97.4%)	2311 (97.6%)	5625 (97.5%)

Data are median (IQR) or n (%). ART, antiretroviral therapy; INSTI, integrase strand-transfer inhibitor; NNRTI, non-nucleoside reverse transcriptase inhibitor; PI, protease inhibitor; TB, tuberculosis.

a Including homemaker/subsistence farmer.

The primary endpoint was reached by 2649 (77.9%) in the VITAL group and by 1759 (74.3%) in the eSOC group, with no significant difference between groups (aOR 1.18 [95% CI 0.95–1.46]; aRD 0.03 [−0.01 to 0.07]; intraclass correlation coefficient 0.01; Table 2). The lower bound of the 95% CI for the aOR was above the pre-defined non-inferiority margin of 0.8 but not above 1.0, indicating that the criterion for non-inferiority was met, while superiority was not established. For the per protocol analysis, 1675 in the VITAL group and 1067 in the eSOC group were excluded, resulting in 1726 and 1302 in the VITAL and the eSOC group respectively. The primary endpoint was reached by 1219 (70.6%) participants in the VITAL group and by 836 (64.2%) in the eSOC group (aOR 1.26 [0.92–1.72]; aRD 0.05 [−0.02 to 0.12]).

**Table 2:** Primary analyses and endpoints.

	VITAL group (n = 3401)	Enhanced standard of care group (n = 2369)	Adjusted risk difference (95% CI)	Adjusted odds ratio (95% CI)	p-value^a^
Primary endpoint, mITT analysis	2649 (77.9%)	1759 (74.3%)	0.03 (−0.01–0.07)	1.18 (0.95–1.46)	0.13
Primary endpoint, details					
Viral load <50 copies per mL	2649 (77.9%)	1759 (74.3%)			
Viral load 50–999 copies per mL	110 (3.2%)	50 (2.1%)			
Viral load ≥1000 copies per mL	28 (0.8%)	30 (1.3%)			
No documented viral load	614 (18.1%)	530 (22.4%)			
Primary endpoint, per-protocol analysis	1219 (70.6%)^d^	836 (64.2%)^e^	0.05 (−0.02–0.12)	1.26 (0.92–1.72)	0.16
Primary endpoint, sensitivity analyses (mITT)					
mITT, suppression defined as <1000 copies per mL	2759 (81.1%)	1809 (76.4%)	0.04 (0.00–0.08)	1.28 (1.01–1.64)	0.052
mITT, using imputations for missing endpoints	3166 (93.1%)	2182 (92.1%)	–f	1.14 (0.93–1.40)	0.23
Primary endpoint, subgroup analyses (mITT)					
By age					0.52^b^^,^^c^
18–24 years	112/200 (56.0%)	97/157 (61.8%)			
25–39 years	963/1281 (75.2%)	703/1002 (70.2%)			
40–59 years	1207/1481 (81.5%)	769/969 (79.4%)			
≥60 years	367/439 (83.6%)	190/241 (78.8%)			
By sex					0.49^b^
Female	1765/2256 (78.2%)	1145/1547 (74.0%)			
Male	884/1145 (77.2%)	614/822 (74.7%)			
By cell phone access					0.45^b^
Yes	2054/2615 (78.5%)	1348/1783 (75.6%)			
No	595/786 (75.7%)	411/586 (70.1%)			

CI, confidence interval; IQR, interquartile range; mITT, modified intention to treat.

a p-values for the adjusted odds ratio or adjusted hazard ratio were calculated using likelihood ratio tests and refer to the superiority comparison.

b p-value from the interaction term between the trial group and this variable obtained through likelihood ratio tests.

c Calculated using the continuous variable.

d Not included in per-protocol analysis: 1675.

e Not included in per-protocol analysis: 1067.

f could not be estimated.

In sensitivity analyses, increasing the threshold for viral suppression to less than 1000 copies per mL in the mITT population led to a modest increase in the effect estimates favouring the VITAL group (aOR 1.28 [1.01–1.64]; aRD 0.04 [0.00–0.08]). Imputing missing viral loads in the primary endpoint window in the mITT population did not meaningfully alter outcomes (aOR 1.14 [0.93–1.40]). Prespecified effect modification analyses for sex, age group, and cell phone access showed no evidence for a credible interaction (Table 2). Adjusting the primary analysis for viral load in the year prior to enrolment, age, and sex did not meaningfully change the primary outcome (Supplement Section D). All-cause mortality and TB diagnosis at 24 months were similar between groups (80 [2.4%] in the VITAL group and 53 [2.2%] in the eSOC group, adjusted hazard ratio 1.10 [0.78–1.58]; 15 [0.4%] in the VITAL group and 14 [0.6%] in the eSOC group, aOR 0.70 [0.30–1.63]). Disengagement from care at 24 months was lower in the VITAL group (156 [4.6%] in the VITAL group and 167 [7.1%] in the eSOC group; aOR 0.67 [0.48–0.93]). All other safety and secondary endpoints did not differ significantly between study groups (Table 3).

**Table 3:** Secondary analyses and endpoints, as well as provider resources.

	VITAL group (n = 3401)	Enhanced standard of care group (n = 2369)	Measure	Estimate (95% CI)	p-value^a^
Secondary endpoints: basis for additional claims (mITT)					
Primary endpoint among participants with a viral load ≥50 copies per mL during the first 12 months (n = 402)	170 (67.7%)	98 (64.9%)	aOR	1.09 (0.63–1.86)	0.76
Secondary endpoints: safety (mITT)					
All-cause mortality at 12 months^d^	43 (1.3%)	32 (1.4%)	aHR	0.98 (0.63–1.61)	0.93
All-cause mortality at 24 months^d^	80 (2.4%)	53 (2.2%)	aHR	1.10 (0.78–1.58)	0.59
Tuberculosis diagnosis at 12 months	6 (0.2%)^e^	4 (0.2%)^f^	aOR	–i	–i
Tuberculosis diagnosis at 24 months	15 (0.4%)^g^	14 (0.6%)^h^	aOR	0.70 (0.30–1.63)	0.40
Disengagement from care at 12 months	74 (2.2%)	70 (3.0%)	aOR	0.71 (0.38–1.36)^e^	0.29
Disengagement from care at 24 months	156 (4.6%)	167 (7.1%)	aOR	0.67 (0.48–0.93)	0.033
Secondary endpoints: supportive evidence (mITT)					
Median time to follow-up viral load in case of a viral load ≥50 copies per mL, in days (n = 1170 among 829 participants)	275 (IQR 168–370)	252 (IQR 152–363)	Adjusted mean difference	9.3 (−20.9–39.2)	0.50
Median rate of clinic visits at 24 months after enrolment, per year	2.5 (IQR 2–3.5)	2.5 (IQR 2–3)	aIRR	1.24 (0.98–1.55)	0.075
Proportion of participants with ART regimen modification due to virologic failure at 12 months among participants with virologic failure^c^	1/21 (4.8%)	0/10 (0.0%)	–	–j	–j
Proportion of participants with ART regimen modification due to virologic failure at 24 months among participants with virologic failure^c^	2/46 (4.3%)	2/36 (5.6%)	–	–j	–j
Median time to ART regimen modification in case of virological failure, in days^c^ (n = 4)	272 (IQR 185–350)	418 (IQR 310–525)	–	–j	–j
Proportion of participants who ever received tuberculosis preventive therapy	3149 (92.6%)	2208 (93.2%)	aOR	0.94 (0.57–1.62)	0.83
Provider resources					
Median time nurse spent per participant visit, in minutes (n = 37)	12 (IQR 7–17)	18.5 (IQR 10.5–22)	–	–j	0.11^b^
Median time counsellors spent per participant visit, in minutes (n = 37)	0 (IQR 0–0)	3 (IQR 0–5)	–	–j	0.012^b^

aOR, adjusted odds ratio; aHR, adjusted hazard ratio; aIRR, adjusted incidence rate ratio; ART, antiretroviral therapy; CI, confidence interval; IQR, interquartile range; mITT, modified intention to treat.

a p-values for the adjusted odds ratio or adjusted hazard ratio were calculated using likelihood ratio tests and refer to the superiority comparison.

b p-values for provider resources were calculated using Wilcoxon rank-sum tests.

c Virological failure was defined as either two viral load measurements ≥1′000 copies per mL or three measurements ≥20 copies per mL until a national guideline update in January 2022, and as two consecutive viral loads ≥50 copies per mL thereafter.

d One death was excluded from mortality analyses due to a missing date of death.

e Had missing data: 164.

f Had missing data: 427.

g Had missing data: 155.

h Had missing data: 417.

i Could not be estimated.

j Not attempted to estimate.

Among secondary endpoints providing supportive evidence, healthcare process measures were comparable between groups (Table 3). Among participants with a viral load ≥50 copies per mL, the median time to follow-up viral load testing was 275 days (IQR 168–370) in the VITAL group and 252 days (IQR 152–363) in the eSOC group (adjusted mean difference 9.3 [−20.9–39.2]). The rate of clinic visits over 24 months was 2.5 visits per year in both groups (aIRR 1.24 [0.98–1.55]). ART regimen modifications in participants with virologic failure were rare, occurring in 2 out of 46 (4.3%) in the VITAL group and in 2 out of 36 (5.6%) in the eSOC group. The cumulative coverage of TB preventive therapy by 24 months was 92.6% in the VITAL group and 93.2% in the eSOC group by 24 months (aOR 0.94 [0.57–1.62]).

Based on 37 time-and-motion observations, the median time nurses spent per participant visit was 12 min (IQR 7.0–17.0) in the VITAL group and 18.5 min (IQR 10.5–22.0) in the eSOC group. For counsellors, the median time per visit was 0 min (IQR 0.0–0.0) in the VITAL group and 3 min (IQR 0.0–5.0) in the eSOC group (Table 3). These findings were complemented by 19 self-reported workload assessments from nurses, which also did not indicate a meaningful difference in the staff resources required per participant (Supplementary Table S5).

## Discussion

The VITAL trial assessed whether differentiated service delivery, supported by multiple digital health components–individualised automated text messaging and telemedicine for participants receiving ART and clinical decision support for their healthcare providers–could improve treatment outcomes in a rural setting in southern Africa. While the VITAL group demonstrated non-inferiority compared to the eSOC group, we observed no significant improvement in viral suppression after 24 months. Although a sensitivity analysis using a 1000 copies per mL threshold yielded a borderline superior result, its effect size remained comparable. Engagement in care was, however, higher in the VITAL group.

Our findings align with previous evidence both on differentiated HIV service delivery and on digital health-supported care in LMICs. While the intervention combined multiple digital components—clinical decision support for providers and mobile health for participants—we found no evidence that these technologies reduced the rate of clinic visits or improved clinical outcomes. This is consistent with recent Cochrane and umbrella reviews, which reported no or only small effects of digital health interventions on clinical outcomes in HIV care.^12^^,^^14^ Similarly, a multinational, randomised trial found that a two-way mobile phone adherence intervention did not improve viral suppression among participants with second-line ART failure.^23^ The PEBRA trial, which tested a peer educator-supported, app-based differentiated service delivery model for young people in the same setting as VITAL, also did not find improved clinical outcomes.^24^ However, VITAL demonstrated a slightly lower risk of disengagement from care in the intervention group, suggesting that digital health-supported differentiated service delivery may improve continuity of care. This supports WHO's cautious endorsement of digital interventions–highlighting their potential while calling for more rigorous, context-specific evaluations of their effectiveness and safety.^15^

Three further observations regarding HIV care in participating clinics are of particular interest. Regimen adjustments following virologic failure were rare–only 4 of 82 participants with virologic failure received ART modification. This finding underscores the need for both second- and third-line treatment options and improved training and sensitization of healthcare providers on treatment failure and drug resistance. The high proportion of participants missing viral load measurements in the in the primary endpoint window suggests future interventions should focus even more on improving service provision, uptake and follow-up. As an encouraging result we found that both arms had ≥90% cumulative coverage of TB preventive therapy by 24 months, which is above regional estimates.^25^

The VITAL trial has several limitations. First, delays in viral load availability for display and clinical decision support in the VITALapp were common, as reflected by fewer than half of participants being included in the per-protocol analysis. However, the results of the per-protocol analysis suggest that these challenges did not drive the primary outcome of the trial. Second, two mid-trial changes in the Lesotho National ART Guidelines affected the study context. At the time of study design, the standard of care allowed for one-to three-month ART distribution, whereas our intervention–allowing up to twelve-month distribution–was innovative and largely untested. However, following the onset of the COVID-19 pandemic, six-month ART distribution became standard of care, thereby reducing the contrast between the two groups. In addition, the national definition of viral suppression was updated, necessitating the corresponding amendment of the VITALapp recommendations and the primary endpoint. This change is unlikely to have meaningfully impacted the clinical management of participants, as the initial VITALapp cutoff of 20 copies per mL was stricter than the guideline recommendation, leading to a brief period between the guideline rollout and the VITALapp update when actions were triggered at 20 copies per mL rather than 50 copies per mL. Third, we compared the VITAL group to a substantially enhanced standard of care group that included digital documentation–a component that itself is considered a digital health intervention.^15^ This enhancement was necessary to ensure comparability of follow-up data, but may have further reduced the contrast between the groups. Fourth, cluster-randomisation led to a few imbalances between the groups. Participating clinics (clusters) varied in size and participant volume, leading to a higher number of participants in the VITAL group, primarily due to one large clinic (Supplement Table S2). Furthermore, there was partly an imbalance in participant characteristics. Notably, a greater proportion of participants in the VITAL group lived far from their clinic, a potential determinant for disengagement from care. Fifth, although intention to remain at the same facility for the study duration was an inclusion criterion, some participants may have transferred between control and intervention clinics; however, such transfers were infrequent. Finally, due to the nature of the intervention, this was an open-label trial. However, as the primary outcome was an objective laboratory measure and participant tracing and digital documentation ensured comparable reporting across groups, we deem the risk of bias on the primary outcome or main conclusions as minimal.

The VITAL trial has several strengths. It is the first randomised trial to evaluate a digital health package for HIV care that includes components targeting both healthcare providers and participants. The study population is highly representative of adults receiving HIV care at nurse-led clinics in a rural setting in southern Africa. Its pragmatic design–without study-specific visits and relying on data collected through routine care–enables direct conclusions about effectiveness in real-world conditions. Finally, the consistency of findings across sensitivity analyses and subgroups further strengthens confidence in the results.

In conclusion, the non-inferiority of the VITAL trial suggests that broad implementation of digital health-supported differentiated service delivery in southern Africa will depend largely on the cost of digital health solution, user acceptability, and infrastructure readiness. The absence of superiority in clinical outcomes underscores the limitations of digital interventions in the absence of other systemic enablers. In the context of a growing HIV funding shortfall, simpler differentiated service delivery models may currently represent a more efficient allocation of limited resources. However, the digital health landscape is rapidly evolving. As electronic medical record systems achieve full implementation, smartphone penetration and digital literacy increases in rural settings across Africa there will be a critical need to reassess the clinical and cost-effectiveness of optimised digital interventions in HIV care.

## Contributors

NDL conceptualised the study. NT and NDL acquired funding. NDL, NT, ML, AS, AvH, JAB, AA, and JMB designed the study. NDL, NT and ML carry overall responsibility for the trial. NT wrote the statistical analysis plan and conducted all analyses. FC is the senior responsible statistician and checked all analyses. NT, GS, TL, MC, LM, and LU had access to, managed, and verified the underlying study data. MaK and MpK carried district coordination responsibilities and ML oversaw both districts. MM and NC were study nurses. BL and BLN were study physicians. RVB and JH designed and conducted the costing analysis. NT drafted the first version of the manuscript, which was reviewed by all authors.

## Data sharing statement

De-identified, coarse-grained participant data will be made publicly available via the data repository Zenodo (https://zenodo.org/) upon publication. A protocol manuscript has been published^16^ and the trial protocol and statistical analysis plan are available at https://clinicaltrials.gov/study/NCT04527874.

## Declaration of interests

NDL reports having received travel grants to conferences from Gilead Sciences and ViiV Healthcare, and his division received honoraria for consultancies from ViiV Healthcare. AS reports receiving funding from Merck as a clinical trial investigator. RVB serves on a Gilead Sciences DMC and received an honorarium for this role. All other authors declare no competing interests.

## Appendix A Supplementary data

Appendix

Abstract Sesotho

Clinical Protocol
