# Supplementary material for: Integrating clinical decision support and mobile health for differentiated HIV service delivery in Lesotho (VITAL): a cluster-randomised non-inferiority trial
Source: eClinicalMedicine. 2026 Apr 2;94:103850. doi: 10.1016/j.eclinm.2026.103850 (PMC13084333; doi:10.1016/j.eclinm.2026.103850)
Supplement: Appendix [file mmc1.docx]

**Appendix**

**Integrating clinical decision support and mobile health for differentiated HIV service delivery in Lesotho: a cluster-randomised non-inferiority trial (VITAL)**

Table of contents

[Supplement Table 1: Search strategy in PubMed. 2](#_Toc218519637)

[Supplement Table 2: Participants per Site. 3](#_Toc218519638)

[Supplement Table 3: Changes to the study protocol 4](#_Toc218519639)

[Supplement Section A: VITALapp. 7](#_Toc218519640)

[Supplement Section B: Virological failure. 8](#_Toc218519641)

[Supplement Section C: Serious Adverse Events. 8](#_Toc218519642)

[Supplement Table 4: Workload assessments for nurses. 9](#_Toc218519643)

[References. 9](#_Toc218519644)

## Supplement Table 1: Search strategy in PubMed.

| **Search 1** |  |
| --- | --- |
| #5 | #1 AND #2 AND #3 AND #4 AND (“2018/01/01”[Date – Publication] : “2025/05/01”[Date – Publication]) |
| #4 | “Africa” |
| #3 | “randomized controlled trial” OR “controlled clinical trial” OR “randomized” |
| #2 | “digital health” OR “mHealth” OR “mobile health” OR “eHealth” OR “electronic health” OR “clinical decision support” OR “telemedicine” OR “SMS” |
| #1 | “human immunodeficiency virus” OR “human immunodeficiency virus infection” OR HIV OR “antiretroviral treatment” OR “antiretroviral therapy” OR “antiretroviral therapy, highly active” OR “highly active antiretroviral therapy” OR HAART OR ART |
| **Search 2** |  |
| #6 | #1 AND #2 AND #3 AND #4 AND #5 AND (“2018/01/01”[Date – Publication] : “2025/05/01”[Date – Publication]) |
| #5 | “Africa” |
| #4 | “randomized controlled trial” OR “controlled clinical trial” OR “randomized” |
| #3 | “digital health” OR “mHealth” OR “mobile health” OR “eHealth” OR “electronic health” OR “clinical decision support” OR “telemedicine” OR “SMS” |
| #2 | “differentiated service delivery” OR DSD |
| #1 | “human immunodeficiency virus” OR “human immunodeficiency virus infection” OR HIV OR “antiretroviral treatment” OR “antiretroviral therapy” OR “antiretroviral therapy, highly active” OR “highly active antiretroviral therapy” OR HAART OR ART |

Our PubMed search strategy began with a broad search on digital health for HIV care in Africa, followed by a more targeted search on digital health- supported differentiated HIV service delivery.

## Supplement Table 2: Participants per Site.

| Clinic | | Overall (N=5770) | eSOC group (N=2369) | VITAL group (N=3401) | Estimated number of participants (2018)* | |
| --- | --- | --- | --- | --- | --- | --- |
| Butha-Buthe district (%) | | 3007 (52.1) | 1441 (60.8) | 1566 (46.0) | |  |
|  | 1 | 337 (5.8) | 337 (14.2) | 0 (0.0) | | 317 |
|  | 2 | 445 (7.7) | 0 (0.0) | 445 (13.1) | | 418 |
|  | 3 | 177 (3.1) | 177 (7.5) | 0 (0.0) | | 299 |
|  | 4 | 343 (5.9) | 343 (14.5) | 0 (0.0) | | 452 |
|  | 5 | 380 (6.6) | 380 (16.0) | 0 (0.0) | | 403 |
|  | 6 | 378 (6.6) | 0 (0.0) | 378 (11.1) | | 376 |
|  | 7 | 174 (3.0) | 0 (0.0) | 174 (5.1) | | 261 |
|  | 8 | 204 (3.5) | 204 (8.6) | 0 (0.0) | | 308 |
|  | 9 | 278 (4.8) | 0 (0.0) | 278 (8.2) | | 255 |
|  | 10 | 291 (5.0) | 0 (0.0) | 291 (8.6) | | 248 |
| Mokhotlong district (%) | | 2763 (47.9) | 928 (39.2) | 1835 (54.0) | |  |
|  | 11 | 397 (6.9) | 0 (0.0) | 397 (11.7) | | 290 |
|  | 12 | 217 (3.8) | 217 (9.2) | 0 (0.0) | | 330 |
|  | 13 | 149 (2.6) | 149 (6.3) | 0 (0.0) | | 139 |
|  | 14 | 783 (13.6) | 0 (0.0) | 783 (23.0) | | 751 |
|  | 15 | 282 (4.9) | 282 (11.9) | 0 (0.0) | | 418 |
|  | 16 | 342 (5.9) | 0 (0.0) | 342 (10.1) | | 335 |
|  | 17 | 280 (4.9) | 280 (11.8) | 0 (0.0) | | 388 |
|  | 18 | 313 (5.4) | 0 (0.0) | 313 (9.2) | | 252 |

*Estimated for sample size calculation using routine data

## Supplement Table 3: Changes to the study protocol

| **Change** | **Date** | **Description** |
| --- | --- | --- |
| Change in VITAL algorithm | 16.12.2022; approved by National Health Research Ethics Committee of Lesotho | To align viral load cutoff with amendment to national ART guidelines (sixth edition, January 2022). |
| Primary outcome | 17.04.2024; approved by National Health Research Ethics Committee of Lesotho (protocol version v1.4, dated 23.06.2023) | The cutoff for viral suppression in the primary endpoint was amended from 20 copies per mL to 50 copies per mL during the trial to align with the 2022 amendment to the Lesotho national ART guidelines and considering the detection threshold of 40 copies per mL of the point-of-care viral load testing platforms increasingly used in Lesotho. |
| Secondary outcomes moved to separate manuscripts:   - SMS result requests - SMS delivery success - Nurse call-back use - automated TB screening - cervical cancer screening - participant appreciation of the VITAL model | 03.09.2024; approved by Sponsor (SAP v1.0, dated 02.09.2024) | The reason for these removals is that each would require substantial additional detail and supporting information beyond the scope of this article. |
| Secondary outcomes dropped:   - provider appreciation of the VITAL model | 03.09.2024; approved by Sponsor (SAP v1.0, dated 02.09.2024) | Dropped due to resource constraints. |
| Changes to secondary outcomes:   - the cutoff of viral suppression was adapted to 50 copies per mL - Switch was changed to ART regimen modification | 03.09.2024; approved by Sponsor (SAP v1.0, dated 02.09.2024) | - In alignment with primary outcome - nation-wide roll-out of dolutegravir in Lesotho led to a change of the guidelines when to switch to second-line due to ART failure and core agent changes became extremely rare among persons taking dolutegravir. |
| Sensitivity analyses added:   - primary endpoint with imputation for missing viral loads - primary endpoint in a per-protocol population | 03.09.2024; approved by Sponsor (SAP v1.0, dated 02.09.2024) | Sensitivity analyses were added to assess the robustness of the primary endpoint. |
| Adjustment for individual-level baseline covariables | 03.09.2024; approved by Sponsor (SAP v1.0, dated 02.09.2024) | Although considered at the protocol stage, the Statistical Analysis Plan did not include covariate adjustment. This decision was driven by anticipated substantial missingness in the most prognostic individual-level variable - pre-enrolment viral load obtained from routine-care records within the year before enrolment - which would have limited both the feasibility and interpretability of adjusted analyses. A post-hoc analysis adjusting for individual-level baseline covariables was added to the Supplementary material at revision stage. |
| ITT to mITT | 10.2024-01.2025; during final data cleaning | We excluded participants who were retrospectively identified as ineligible, as well as those with missing core data required to determine the primary outcome (e.g., participant identifier or enrolment site). |
| Costing analysis | 02.2025-05.2025; during analysis | Based on the limited differences observed in provider time and visit frequency between groups, a full economic evaluation was not pursued as it was unlikely to yield additional meaningful insight. |

## Supplement Section A: VITALapp.

**Supplement Figure 1:** VITALapp - mobile health preferences collected at enrolment by study staff. Visible and updatable for the nurse.

**
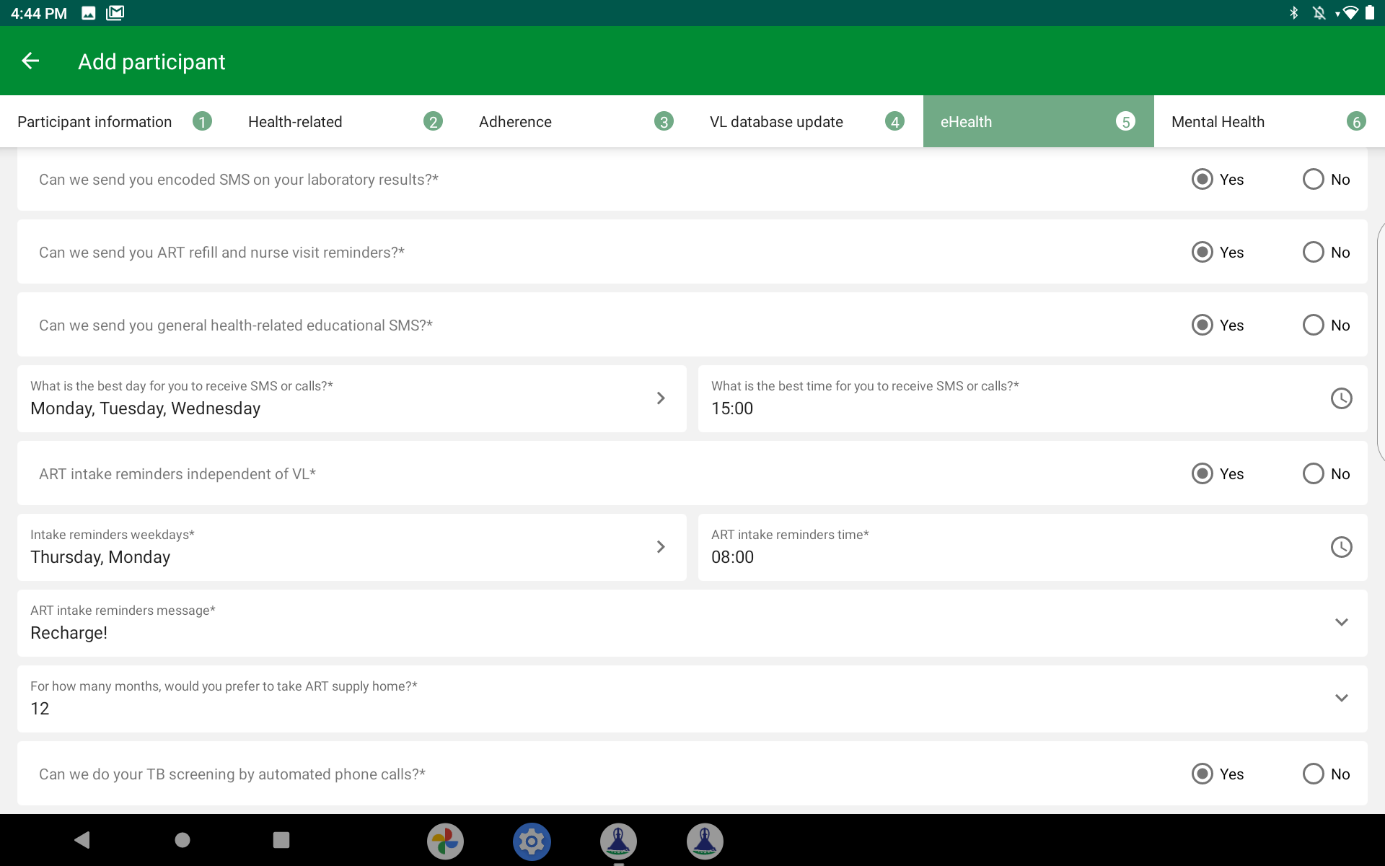
**

**Supplement Figure 2:** VITALapp – nurse overview page, based on viral load results from the VICONEL database (exemplary data)
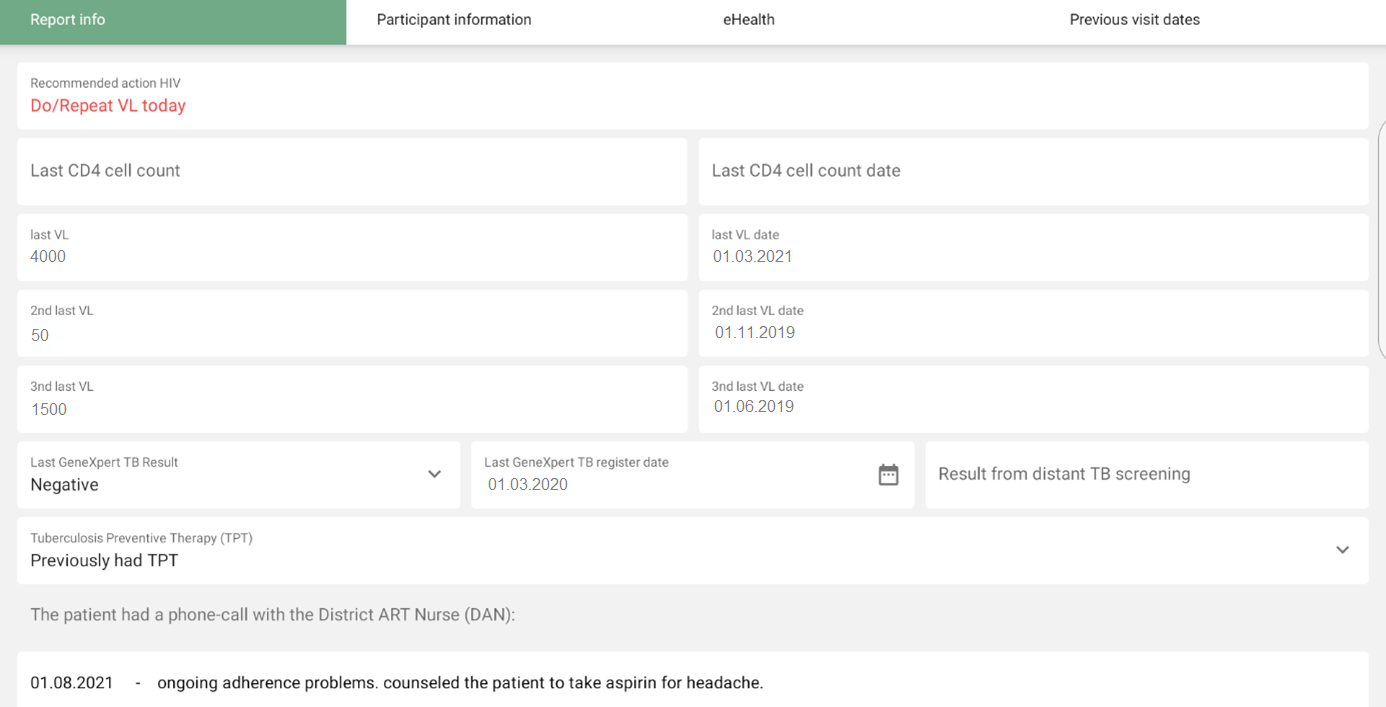


## Supplement Section B: Virological failure.

At start of the VITAL trial, virological failure was defined as either two viral load measurements ≥1’000 copies/mL or three measurements ≥20 copies/mL. Following the guideline amendment in January 2022, our definition aligned with the standard of care, defining virological failure as two consecutive viral loads ≥50 copies/mL.^1,2^

For switching to a second- or third-line regimen, the Lesotho Guidelines require the health care provider to submit a written report summarising the clinical, therapeutic, and laboratory history to the ART Advisory Committee. Based on this report, the committee may approve a treatment switch, request additional information or tests (e.g. an HIV drug resistance test), or decline the request.

## Supplement Section C: Serious Adverse Events.

Health care personnel at VITAL clinics were instructed to notify the VITAL nurse - or, if unavailable, the VITAL local principal investigator - within 72 hours of becoming aware of a serious adverse event (SAE). The VITAL local principal investigator was required to inform the Sponsor/Chief Investigator within 72 hours of becoming aware of the SAE. If a causal relationship with the VITAL intervention could not be ruled out, the Sponsor/Chief Investigator was obligated to notify the local ethics committee in Lesotho within 72 hours of becoming aware of the event. The annual report to the Ethics Committee in Lesotho on the VITAL study progress included a line listing of all SAEs, regardless of their causal relationship to the VITAL intervention.

## Supplement Section D: Post-hoc analysis adjusting for baseline variables.

A post-hoc sensitivity analysis adjusted the primary analysis for viral load in the year prior to enrolment, age, and sex, in line with guidance by Holmberg and colleagues to include only strongly prognostic factors^3^. As viral load measurements at enrolment were unavailable for most participants, viral load in the year prior to enrolment was categorised as suppressed, low-level, or high; missing values were further distinguished between participants who initiated ART within six months before enrolment, for whom no viral load measurement was expected, and those with missing values despite an expected measurement according to guidelines (**Supplement Table 4**). The adjusted odds ratio for the primary endpoint was 1·14 [95% CI 0·93 to 1·40], with an adjusted risk difference of 0·03 [–0·01 to 0·07]. Overall, this post-hoc analysis did not materially change the effect estimates or study conclusions.

| **Supplement Table 4.** Viral load in the year prior to enrolment | | |
| --- | --- | --- |
|  | **Enhanced standard of care group** | **VITAL group** |
| <50 copies per mL | 1706 (72.0%) | 2502 (73.6%) |
| 50-999 copies per mL | 115 (4.9%) | 216 (6.4%) |
| ≥1000 copies per mL | 49 (2.1%) | 51 (1.5%) |
| Missing viral load (unjustified) | 350 (14.8%) | 451 (13.3%) |
| Missing viral load (justified: new on ART) | 149 (6.3%) | 181 (5.3%) |

## Supplement Table 5: Workload assessments for nurses.

| **Nurse** | **Enhanced standard of care group (N=10)** | | **VITAL group**  **(N=9)** | |
| --- | --- | --- | --- | --- |
|  | Never / Occassionally / Regularly | Median (IQR) Weighted minutes | Never / Occassionally / Regularly | Median (IQR) Weighted minutes |
| Activities only for study purpooses | *8/1/0* | **0** (0-0) | *9/0/0* | **0** (0-0) |
| File retrieval or filing | *4/6/0* | **1** (0-1) | *4/5/0* | **1** (0-2) |
| History taking & charting NOT using VITAL | *0/1/9* | **5** (5-15) | *2/0/7* | **3** (1-5) |
| History taking & charting using VITAL | *3/2/5* | **1.5** (0-2) | *2/0/7* | **5** (2-5) |
| Measuring anthropometrics & vitals | *0/3/7* | **3** (2-5) | *2/0/7* | **2** (1-5) |
| Other administrative activities for participants | *6/2/2* | **0** (0-5) | *6/1/2* | **0** (0-3) |
| Other clinical activities for participants | *4/2/4* | **3** (0-10) | *6/1/2* | **0**- (0-10) |
| Patient Consultation (including physical exams, specimin collection, discussions & advice) | *0/0/10* | **15** (10-20) | *0/0/9* | **15** (5-30) |
| Patient discharge / scheduling | *0/0/7* | **1** (0.75- 1) | *0/0/5* | **2** (2-2) |
| Pharmacy | *1/5/4* | **3.5** (2-5) | *0/1/8* | **5** (2-5) |
| Pill count & counseling | *6/4/0* | **0** (0-4) | *2/7/0* | **3** (2-5) |
| Post-visit charting & documentation | *0/2/8* | **5** (3-10) | *0/0/9* | **5** (5-5) |
| VITALapp updating, uploading, or usage without clinet | *0/2/7* | **2.5** (1-3) | *0/2/7* | **2** (2-2) |
| Waiting (for client, for results, or otherwise) | *3/4/3* | **7.5** (0-15) | *6/1/2* | **0** (0-0) |

Times, medians, and IQRs are in minutes. Weighted indicates that responses marked as “occasionally” (rather than “regularly”) had their reported times counted at half value. IQR = interquartile range.

## References.

1. Ministry of Health Lesotho. NATIONAL GUIDELINES ON THE USE OF ANTIRETROVIRAL THERAPY FOR HIV PREVENTION AND TREATMENT, Sixt Edition. (2022).

2. Ministry of Health, Government of Lesotho. Addendum to the national guidelines on the use of antiretroviral therapy for HIV prevention and treatment. (2019).

3. Holmberg, M. J. & Andersen, L. W. Adjustment for Baseline Characteristics in Randomized Clinical Trials. *JAMA* **328**, 2155–2156 (2022).
