# Supplementary material for: Integrating clinical decision support and mobile health for differentiated HIV service delivery in Lesotho (VITAL): a cluster-randomised non-inferiority trial
Source: eClinicalMedicine. 2026 Apr 2;94:103850. doi: 10.1016/j.eclinm.2026.103850 (PMC13084333; doi:10.1016/j.eclinm.2026.103850)
Supplement: Abstract Sesotho [file mmc2.docx]

*The following translations were submitted by the authors, and we reproduce them as supplied. They have not been peer reviewed. Our editorial processes have only been applied to the original abstract in English, which should serve as reference for this manuscript*

**Ts’ebetso e kopantseng liqeto tsa bongaka le bophelo bo botle ho sebelisoa marang-rang bakeng sa phano ea litšebeletso tsa HIV Lesotho (VITAL)**

Background / Selelekela:

Mokhatlo oa lefats'e oa bophelo, o thoholetsa tlasa lipehelo tse itseng ts'ebeliso ea marang-rang ho ntlafatsa phano ea lits'ebeletso tsa bophelo, le hao ntse o ananela bopaki bo fokolang ba melemo le likotsi tsa ts'ebeliso ea marang-rang kalafong. Re hlahlobile katleho ea phano ea lits’ebeletso tsa bophelo ka ts’ehetso ea marang-rang e tsepameng ho thusa mokuli mabapi le liphetho tsa kalafo metseng e tikolohong e Boroa ho Aforeka.

Methods / Mokhoa ea tsebetso:

VITAL ke boithuto bo entsoeng litsing tsa bophelo, ka ho ngolisa batho ba baholo ba kalafong ea HIV (ART) metseng e leshome le metso e robong, e entelletsoeng pele ke baoki litsing tsa bophelo Lesotho ho sebelisoa lotho, moo ho bapisuoeng lihlopha tse peli tsa bakuli). Litsi tsena tsa bophelo li ne li khethiloe ka lotho pakeng tsa moo phano ea lits’ebeletso tsa bophelo le ho tlaleha litokomane e leng ea marang-rang, le tšehetso ea liqeto tsa bongaka bakeng sa baoki ka marang-rang. Batho bankang karolo boithutong bona (bakuli) ba ne ba thola ts’eetso ea bophelo bo botle ka marang-rang ka ho fua melaetsa mehaleng ea bona. Bane ba ikhethela naa ba batla tlatsetso ea litlhare (ART) tsa nako e kae hoea selemong (Sehlopa sa VITAL) Tsena li ne li kalloa mabapi le mokhoa o tloaheileng oa tsebetso (eSOC) moo feela litokomane li ne litlatsoa ka mokhoa oa marang-rang.

Sepheo sa mantlha e ne e le ho ts’epahalla hoba kahara tlhokomelo ena ea boithubo le hoba le likomane tse pakang ho theoha hoa sekhahla sa kokoanahloko ea HIV maling (<50 likopi ka mL) nakong ea likhoeli tse 24 (Pakeng tsa nako tsa: likhoeli tse 16-28) tsa boithuto. Karolelano e fetotsoeng ea li-odds (aOR) e hakantsoe ho palo e fetotsoeng ea sepheo sa ho phekola (mITT) ka karolo e seng tlase ho 0.8. Lintlha tsa mantlha tsa ts’ireletseho ea bakuli e bile lipalo tsa mafu ohle, lipalo tsa lits’oaetso tsa lefuba, le ho tlohela kalafo. Boithuto bo ngolisitsoe tlasa ClinicalTrials.gov (NCT02527874) ‘me boithuto bo phethetsoe.

Findings / Liphuphutso:

Lipakeng tsa la 14 Mphalane 2020 le la 30 Hlakubele 2022, ho ngolisitsoe bakuli ba 5809 moo ba 5770 ba ileng ba kengoa tlasa tlhahlobong ka sepheo sa ho phekola (mITT) (3401 sehlopheng sa VITAL le 2369 sehlopheng sa eSOC). Sepheo sa mantlha se fihletsoe ka 2649 (77 · 9%) sehlopheng sa VITAL le ke 1759 (74 · 3%) sehlopheng sa eSOC (aOR 1 · 18 [95% CI 0 · 95 hoea 1 · 46]). Palo ea mafu ka kakaretso le ba fumanoeng bana le lefu la lefuba ea ts’oana/haena phapang lipakeng tsa lihlopha ka bobeli (80 [2 · 4%] sehlopheng sa VITAL le 53 [2·2%] sehlopheng sa eSOC, sekhahla sa ketsahalo (hazard ratio) 1 · 10 [0·78 hoea 1 · 58]; 15 [0·4%] sehlopheng sa VITAL le 14 [0 · 0 0 7] sehlopha sa eSOC aOR 0.6 (0.30 hoea 1 .63). Ho arohana/ ho emisa/ ho tlohela kalafo kapa tlhokomelo hoa bakuli ho bile tlase sehlopheng sa VITAL (156 [4 · 6%] sehlopheng sa VITAL le 167 [7 · 1%] sehlopheng sa eSOC; aOR 0 · 67 [0 · 48 hoea 0 · 93]).

Interpretation / Tlhaloso:

Phano ea lits’ebeletso tsa bophelo tse tsepameng holim’a mokuli ka ts’ehetso ea marang-rang ho fihlela boemo ba ho robatsa koakoana hloko maling le ho ts’ehetsa tlhokomelo e tloaelehileng ea HIV metseng ntle le ho nyolla sekhahla sa litlamora’o tse bosula e bonahetse. Le hoja hosa bonts’oa bophahamo, liphuputso li ts’ehetsa ho kengoa/nyalano e sireletsehileng ea lisebelisoa tsa marang-rang le phano ea litlhare tsa nako e telele. Liphethoho tsa tataiso ea naha ea kalafo ea HIV (ART) nakong ea boithuto li kanna tsa ama phaphang e ka beng e bonahetse lipakeng tsa lihlopha tsa boithuto VITAL le eSOC.

Funding / Tšehetso ea lichelete:

Etsoa ho Moritz Straus-Foundation, Swiss National Science Foundation.
