# Supplementary material for: Integrating clinical decision support and mobile health for differentiated HIV service delivery in Lesotho (VITAL): a cluster-randomised non-inferiority trial
Source: eClinicalMedicine. 2026 Apr 2;94:103850. doi: 10.1016/j.eclinm.2026.103850 (PMC13084333; doi:10.1016/j.eclinm.2026.103850)
Supplement: Clinical Protocol [file mmc3.pdf]

DocuSign Envelope ID: 94DBBACD-5D19-4B54-B740-1BBD7E9B6CE6

THIS DOCUMENT IS CONFIDENTIAL

ASSESSMENT OF A VIRAL LOAD RESULT-DRIVEN AUTOMATED DIFFERENTIATED SERVICE DELIVERY MODEL FOR PARTICIPANTS TAKING ANTIRETROVIRAL THERAPY IN LESOTHO: VIRAL LOAD TRIGGERED ART CARE IN LESOTHO (VITAL)

**Study Type:**	Other Clinical Trial according to the Swiss Human Research Act (HRA) 2014, Ordinance on Clinical Trials in Human Research (ClinO), Chapter 4
**Risk Categorization:**	Risk category A (lowest risk category; according to HRA, ClinO, Chapter 4)
**Study Registration:**	ClinicalTrials.gov NCT04527874
**Sponsor/ Chief investigator:**	Niklaus Labhardt
**Principal Investigator**	Nadine Tschumi (née Bachmann)
**Investigated Intervention:**	Viral load result-driven differentiated care model for participants taking antiretroviral therapy in north-eastern Lesotho
**Protocol ID:**	2020-2019
**Version and Date:**	Version 1.4, 23/06/2023

CONFIDENTIALITY STATEMENT
The information contained in this document is confidential and the property of the sponsor. The information may not - in full or in part - be transmitted, reproduced, published, or disclosed to others than the applicable Competent Ethics Committees and Regulatory Authorityies without prior written authorization from the sponsor except to the extent necessary to obtain informed consent from those who will participate in the study.

PROTOCOL SIGNATURE FORM

**Study Title**	Assessment of a viral load result-driven automated Differentiated Service Delivery Model for participants taking antiretroviral therapy in Lesotho: VIral load Triggered ART care in Lesotho (VITAL)
**Study ID**	220-2019 Amend and Renew 02

The Sponsor / Chief investigator has approved the protocol version 1.4 (dated 23/06/2023) and confirms hereby to conduct the study according to the protocol, current version of the World Medical Association Declaration of Helsinki, and ICH-GCP guidelines as well as the local legally applicable requirements.

Sponsor and Chief investigator:

Name: Niklaus Labhardt

Date: 23.06.2023 | 12:20 MESZ Signature: Niklaus Labhardt

Principal investigator:

Name: Nadine Tschumi (née Bachmann)

Date: 23.06.2023 | 11:51 MESZ Signature: Nadine Tschumi

Local Principal Investigator:

Name: Malebanye Lerotholi

Date: 23.06.2023 | 12:33 MESZ Signature: Malebanye David Lerotholi

1 TABLE OF CONTENTS

1 TABLE OF CONTENTS 2
2 GLOSSARY OF ABBREVIATIONS 4
3 STUDY SYNOPSIS 6
4 BACKGROUND 9
4.1. The HIV epidemic in sub-Saharan Africa 9
4.2. Rationale for the VITAL trial 9
4.3. The HIV epidemic in Lesotho and the setting of the VITAL trial 10
5 STUDY OBJECTIVES AND DESIGN 10
5.1. Hypothesis and primary objective 10
5.2. Primary and secondary endpoints 10
5.3. Study design 12
5.3.1. Stratification criteria 12
5.4. Study intervention 12
6 STUDY POPULATION AND STUDY PROCEDURES 14
6.1. Inclusion and exclusion criteria 14
6.2. Data management 15
6.3. Recruitment, screening and informed consent procedure 15
6.4. Study procedures 16
6.4.1. Study duration 16
6.4.2. Enrolment clinic visit 16
6.4.3. Follow-up study procedures 16
6.5. Withdrawal and discontinuation 23
7 STATISTICS AND METHODOLOGY 23
7.1. Statistical analysis plan and sample size calculation 23
7.1.1. Design 23
7.1.2. Non-inferiority margin 23
7.1.3. Sample size 23
7.1.4. Analyses of clinical outcome 24
7.1.5. Cost-effectiveness or budget-impact analysis 25
7.2. Handling of missing data and drop-outs 25
8 NESTED STUDIES 26
8.1. aDSDM for Tuberculosis prevention and care 26
8.1.1. Background 26
8.1.2. Methods 26
8.1.3. Discussion 26
8.2. Integrating mental health assessment for optimized patient outcomes 26
8.2.1. Background 26
8.2.2. Methods 26
8.2.3. Discussion 27
8.3. The ideal VL threshold for participants who should get a 3-month FU VL 28
8.3.1. Background 28
8.3.2. Methods 28

DocuSign Envelope ID: 94DBBACD-5D19-4B54-B740-1BBD7E9B6CE6

8.3.3. Discussion 28
8.4. Resistance survey at months 0 and 24 28
8.4.1. Background 28
8.4.2. Methods 28
8.5. The impact of an aDSDM on cervical cancer screening coverage 28
8.5.1. Background 28
8.5.2. Methods 28
8.5.1. Hypothesis 29
9 REGULATORY ASPECTS AND SAFETY 31
9.1. Serious Adverse Events 32
9.1.1. Reporting of SAEs 32
9.1.2. Follow up of SAEs 32
9.2. Periodic safety reporting 33
9.3. Amendments 33
9.4. (Premature) termination of study 33
9.5. Insurance 33
10 FURTHER ASPECTS 33
10.1. Risk-benefit assessment 33
10.2. Overall ethical considerations 34
11 QUALITY CONTROL AND DATA PROTECTION 34
11.1. Quality measures 34
11.2. Data recording and source data 34
11.3. Confidentiality and coding 35
11.4. Retention and destruction of study data and biological material 35
12 MONITORING AND REGISTRATION 35
13 FUNDING / PUBLICATION / DECLARATION OF INTEREST 36
14 REFERENCES 36
15 APPENDICES 39
15.1. Case report form 1: Variables collected at enrolment 39
15.2. Case report form 2: Data downloaded and updated by nurse 43
15.3. Case report form 3: Automatically generated second-line request 47
15.4. Case report form 4: Pharmacy dispense and tracing list generated 49
15.5. Case report form 5: Data from District ART nurse 50
15.6. Additional documents 52

DocuSign Envelope ID: 94DBBACD-5D19-4B54-B740-1BBD7E9B6CE6

2 GLOSSARY OF ABBREVIATIONS

AE	*Adverse Event*
aDSDM	automated differentiated service delivery model
ART	*Antiretroviral Therapy*
ASR/DSUR	*Annual Safety Repot / Development Safety Report*
BASEC	*Business Administration System for Ethical Committees*
CEA	*Cost-effectiveness analysis*
ClinO	*Ordinance on Clinical Trials in Human Research (in German: KlinV, in French: OClin, in Italian: OSRUm)*
CMD	*common mental disorder*
CRF	*Case Report Form*
CTCAE	*Common Terminology Criteria for Adverse Events*
CTX	*Co-trimoxazole*
DAN	*VITAL district ART nurse*
DSD	*Differentiated Service Delivery*
DTG	*Dolutegravir*
EAC	*Enhanced Adherence Counselling*
FADP	*Federal Act on Data Protection (in German: DSG, in French: LPD, in Italian: LPD)*
eCRF	*electronic Case Report Form*
FOPH	*Federal Office of Public Health*
FU	*Follow-up*
GCP	*Good Clinical Practice*
GRT	*Genotypic resistance testing*
HIV	*Human Immunodeficiency Virus*
HRA	*Human Research Act*
ICH	*International Conference on Harmonisation*
ITT	*Intention-to-treat*
IUD	*Intrauterine device*
LTFU	*Loss to follow up*
LIS	*Laboratory Information System*
TB	*Tuberculosis*
TPT	*Tuberculosis Preventive Therapy*
VL	*Viral Load*
ICC	*Inter Cluster Correlation*
WHO	*World Health Organization*
MDR-TB	*multidrug-resistance tuberculosis*
NI	*non-inferiority margin*
OR	*odds ratio*
PAP-test	*Papanicolaou-Test*
PLHIV	*People living with HIV*
PP	*Per-protocol*
SAE	*Serious Adverse Event*
SMS	*Short Message Service*
SSA	*sub-Saharan Africa*

DocuSign Envelope ID: 94DBBACD-5D19-4B54-B740-1BBD7E9B6CE6

SOC	Standard of care
VRA	VITAL records assistant
XDR-TB	extensively drug-resistant tuberculosis

3 STUDY SYNOPSIS

**Study Title**	Assessment of a viral load result-driven automated Differentiated Service Delivery Model for participants taking antiretroviral therapy in Lesotho: VIral load Triggered ART care in Lesotho (VITAL)
**Short Title / Study ID**	VITAL / NCT04527874
**Protocol Version and Date**	23/06/2023
**Study Registration**	Clinicaltrials.gov: NCT04527874
**Study Category and Rationale**	Risk category A (lowest risk category)
**Rationale**	To sustainably provide good quality care to increasing numbers of people living with HIV (PLHIV) receiving antiretroviral therapy (ART), care delivery has to shift from a “one-size-fits-all” approach to differentiated care models. Such models should reallocate resources from patients who are doing well to patient groups who may need more attention, such as those with treatment failure or medical and psycho-social problems. Ideally, such a reallocation allows health systems and patients to save resources while improving quality of care (1–3).<br>One proposed approach to differentiate care and intensity of monitoring is viral load-driven differentiated service delivery (4–6). Reducing the intensity of monitoring in patients with suppressed viral load (VL) and no other clincal problems would substantially reduce the workload at health care facilities and save time and transport cost for patients, thus potentially improve long-term engagement in care (7). Time and resources saved in patients with suppressed VL and no other clinical problems would allow focusing on those participants with elevated viral load and/or other clinical problems (like tuberculosis, which is the most common cause of mortality among PLHIV in sub-Saharan Africa). This may potentially improve PLHIVs’ clinical outcome through intensified adherence support, clinical follow-up and timely switches to second-line ART. In many settings in sub-Saharan Africa, however, the potential of VL monitoring to differentiate care is not exploited and thus constitutes a missed opportunity (5). The work of our consortium in Lesotho has shown that the majority of unsuppressed VLs are not acted upon in a timely manner, be it due to providers and patients not being aware of the results or health care providers not being proficient in the management of treatment failure (8,9).
**Risk / Benefit Assessment**	Anticipated risks to trial participants are minimal and limited to risks related to decreased frequency of clinic visits for participants who are virally suppressed. However, during the COVID-19 pandemic, providing long term supply has become standard of care in most clinics. The study harbours a small social risk of involuntary disclosure due to (encoded) text-messages. The trial has the following potential health benefits for individual participants who receive care at one of the intervention clinics:<br>1. Decreased time to next clinic visit for participants with unsuppressed VL<br>2. Health care workers will have more time to focus on participants with high VLs<br>3. Choice of most suitable adherence support option if needed or visit frequency if virally suppressed, will give participants ownership of their health and exclude intensive clinic visit schedules as a reason for defaulting care<br>4. Decreased time to second-line ART switch through automatically generated second-line application forms<br>5. Empowerment of patients through direct communication of their viral load results<br>6. Improvement of viral load result communication from laboratory to a health care worker<br>Since this trial tests a potentially cost-effective or cost-saving monitoring strategy for people taking ART, which is crucial for the sustainability of VL monitoring in sub-Saharan Africa (SSA), it has a high social and scientific value.
**Objectives**	The **primary objective** is to assess if the proposed automated differentiated service delivery model (aDSDM) is at least non-inferior in terms of clinical outcomes and if it is cost-saving<br>- The proposed aDSDM is non-inferior in the proportion of participants engaged in care and virally suppressed at 24 months follow-up (intention-to-treat (ITT) population)<br>**Secondary hypotheses:**<br>- The proposed aDSDM is superior in the proportion of participants engaged in care and virally suppressed at 24 months follow-up (ITT)<br>- The proposed aDSDM results in fewer clinic visits while being at least non-inferior with regard to clinical outcomes<br>- The proposed aDSDM is cost-effective (if superior in terms of clinical outcomes) / cost-saving if non-inferior in terms of clinical outcomes<br>The **secondary objective** is to build through VITAL a well-documented patient cohort at participating clinics that allow further interventional studies responding to emerging health needs within the cohort. Such interventions would then be designed as trials within cohorts (TWICS) and submitted to NH-REC as amendments of this protocol.
**Endpoints**	**Primary endpoint**

	Proportion of participants engaged in care (defined as documented visit attendance) with documented viral suppression (<50 copies/mL) 24 months (16-28 months) after enrollment*<br><br>**Secondary endpoints which may become the basis for additional claims**<br>1. Proportion of participants with sustained viral suppression (defined as >1 VL <50 copies/mL) during 24 months (16-28 months) follow-up*<br>2. Proportion of participants with sustained viral suppression (defined as >1 VL <1000 copies/mL) during 24 months (16-28 months) follow-up*<br>**Secondary endpoints indicative of clinical benefit or harm**<br>1. Mortality rate at 12 and 24 months after enrollment<br>2. Proportion of patients with confirmed TB diagnosis at 12 and 24 months after enrollment<br>3. Disengagement from care at 12 and 24 months after enrollment<br><br>**Secondary endpoints expressing supportive evidence**<br>- Time to follow-up VL in case of an unsuppressed VL (≥ 50 copies/mL)<br>- Time to switch of ART regimen in case of virologic failure<br>- Rate of clinic visits at 24 months after enrollment<br>- Proportion of participants with ART modification due to virologic failure<br>- Proportion of participants diagnosed with TB at 12 and 24 months after enrollment<br>- Proportion of participants receiving a course of TPT<br>In intervention clusters only:<br>- Proportion of participants requesting a VL result notification through SMS<br>- Proportion of SMS delivered successfully<br>- Proportion of participants using the call-back option through District ART Nurse<br>- Proportion of participants screened positive fo TB by automated call<br>- Proportion of participants appreciating the automated differentiated service delivery model Proportion of health care providers appreciating the automated differentiated service delivery model<br>- Proportion of females screened for cervical cancer according to national guidelines<br><br>**Subgroup analyses**<br>1. Proportion of participants with viral re-suppression (<50 copies/mL) 24 months (16-28 months) after enrollment among all participants with an unsuppressed VL (≥ 50 copies/mL) during the first 12 months of follow-up*<br>2. Proportion of participants with viral re-suppression (<1000 copies/mL) 24 months (16-28 months) after enrollment among all participants with an unsuppressed VL (≥ 1000 copies/mL) during the first 12 months of follow-up*<br><br>1. **Sensitivity analysis**Proportion of participants engaged in care (defined as documented visit attendance) with documented viral suppression (<1000 copies/mL) 24 months (16-28 months) after enrollment*
**Study Design**	Multicenter cluster-randomized non-inferiority trial
**Statistical Considerations**	Analyses will be performed following CONSORT guidelines for cluster-randomised trials. Focusing on our binary primary endpoint and odds ratios (ORs) as effect measures, we aim at testing the null hypothesis H_0: OR ≤ NI versus H_A: OR > NI, where NI represents the prespecified level of non-inferiority. Clusters will be set as a unit of randomisation (stratified by baseline clinic performance), whereas individuals are set as a unit of analysis.
**Inclusion- / Exclusion Criteria**	On an **individual level**, the inclusion criteria for the VITAL trial are the following:<br>- Taking antiretroviral therapy (independent of viral suppression)<br>- ≥ 18 years old<br>- Written informed consent<br>- intention to remain in the same facility for the duration of the trial as expressed at enrollment<br>- not enrolled in another study if judged as non-compatible by the (Local) Principal Investigator<br>On a **cluster level**, inclusion criteria for the VITAL trial are the following:<br>- nurse-led public or missionary clinic in the districts of Butha-Buthe and Mokhotlong<br>- consent of clinic management (signed agreement with clinic management)<br>- access to the internet (internet connection must not be constant, but there must be possibility to down- and upload information daily)<br>- the clinic sends VL samples to Butha-Buthe laboratory for analysis
**Number of Participants with Rationale**	We calculated the sample size for the non-inferiority cluster randomised design and chose a margin of non-inferiority for the odds ratio of reaching our primary endpoint of engagement in care with documented viral suppression of 0.8.<br>With a minimum of 8 clusters in the intervention group and a minimum of 8 clusters in the control group, we will be well powered (>80% power) to detect one-sided differences of >10% in the proportion of participants who were engaged in care and virally suppressed in the intervention compared to the control arm.

**Study Intervention**	The concept of the proposed aDSDM is to use VL results, other clinical characteristics (TB screening results) and participants' preference to automatically triage participants into groups requiring different levels of attention and care. Innovatively, triaging of participants will be done automatically capitalising on an existing VL database platform. The implemented aDSDM will differentiate care according to three elements:<br>- clinical characteristics (with focus on VL measurement)<br>- sub-population (women, men)<br>- participants' and health care providers' preferences<br>To ensure effective flow of information, VL results and other relevant information is sent directly to participants' phones, whereas health care providers receive results directly on their study tablet together with the recommended action. Further features of the platform are preference-based tailored adherence reminders and automated calls to participants for symptomatic tuberculosis screening. The proposed aDSDM is designed for being scaled up at national and regional level as it mainly builds on automated triage and communication with participants and health care workers, thus not requiring additional human resources.
**Control Intervention**	In control clusters, a slightly improved standard of care is provided. Improved means that before study start clinic staff is again trained and mentored on national HIV guidelines.
**Study procedures**	VITAL will enrol participants for a period of 6-12 months, and each participant will be followed-up for 24 months.<br>In both arms, participants will be enrolled using questionnaires collecting socio-demographic, clinical and therapeutic baseline data. All participants will be linked to the study-database where visits and laboratory parameters are captured. In the intervention arm participants are followed up according to the aDSDM. Control clusters follow the standard of care.
**Study Duration and Schedule**	The estimated duration is 47 months<br>Planned 09/2020 of First-Participant-In<br>Planned 07/2024 of Last-Participant-Out
**Nested studies**	1. aDSDM for Tuberculosis screening, prevention and care<br>2. Integrating mental health assessment for optimized patient outcomes<br>3. The ideal VL threshold for participants who should get a 3-month FU VL<br>4. Resistance survey at months 0 and 24<br>5. The impact of an aDSDM on cervical cancer screening coverage<br>6. Qualitative nested studies
**Sponsor/ Chief Investigator**	Niklaus Labhardt
**Principle investigator**	Nadine Tschumi (née Bachmann)
**Principle Investigator Lesotho**	Malebanye Lerotholi
**Study Centers**	18 clinics from Butha-Buthe and Mokhotlong, in northern Lesotho
**Data privacy**	Trial and participant data will be handled with uttermost discretion and are only accessible to authorised personnel who require the data to fulfil their duties within the scope of the study. The data management of this study will be performed using an extension of an existent VL database, incorporating various safety measures.
**Ethical consideration**	Establishing cost-effective/saving monitoring strategies for people taking ART is crucial for the sustainability of ART programs in sub-Saharan Africa, and VL-driven differentiated service delivery models have been proposed to achieve this goal. The VITAL trial tests whether an aDSDM is non-inferior and cost-saving and aims at improving HIV care in Lesotho.<br>The evidence generated in the VITAL trial is intended to inform future national and international guidelines and policies. If successful, the tested aDSDM has the potential to be scaled up to national level. Furthermore, evidence generated from nested studies may inform local/national policies.
**GCP Statement**	This study will be conducted in compliance with the protocol, the current version of the Declaration of Helsinki, the ICH GCP E6(R2), the HRA as well as other locally relevant legal and regulatory requirements.

188

4 BACKGROUND

4.1. The HIV epidemic in sub-Saharan Africa

Sub-Saharan Africa (SSA) is home to 25.6 million people living with HIV (PLHIV), corresponding to 68% of the global burden of the epidemic (10). The number of people on antiretroviral therapy (ART) rose rapidly across SSA to 16.4 million by the end of 2018 (10,11).

Since 2013 routine viral load (VL) monitoring is recommended by the World Health Organization (WHO) as the preferred option to identify treatment failure of patients taking ART in resource-limited settings (12). Factors that made the introduction of routine VL monitoring possible in resource-limited settings include increased availability of VL testing, decreasing costs of VL assays, growing evidence of resistance development both at an individual and at the population level (13), universal test and treat strategies, and decreasing costs of ART (5). The WHO recommends VL measurement 6 months after the initiation of the empiric first-line ART and every 12 months thereafter. Furthermore, the WHO recommends that patients with a VL ≥1000 copies/mL (c/mL) undergo enhanced adherence counselling (EAC) and repeat VL testing after 3-6 months. Only a second VL ≥1000 c/mL triggers switching to second-line ART. In most countries in SSA, all patients, independent of VL results, are attending clinics every 1 to 3 months for clinical assessment. While there is little evidence that this would benefit patients' clinical outcome (14,15), it represents a challenge to the capacity of local health care facilities. At the same time, many ART programs fail to switch patients with a virologic failure promptly (8). Delays frequently exceed one year, leading to morbidity, mortality, elevating the risk of (additional) drug resistance development and onward transmission of the (potentially resistant) virus (16–20). Thus, the potential of VL monitoring is not yet optimised in SSA, since VL results often do not trigger action (19,21–26) and are not used to guide differentiated care (5).

4.2. Rationale for the VITAL trial

The updated WHO consolidated guidelines on the use of ART recommend providing client-centred and high-quality care to all PLHIV, including service delivery recommendations based on *differentiated service framework* (1,2). It is broadly accepted that a "one-size-fits-all" model of HIV services does not represent a sustainable solution for the provision of access to ART and support services for the 37 million PLHIV today. Instead, health systems will need to adapt HIV services to specific client populations (according to clinical and socio-demographic characteristics) and contexts (3,27).

At the same time, establishing cost-effective monitoring strategies for people taking ART is crucial for the sustainability of ART programs in SSA (4). VL monitoring still represents a substantial cost in resource-limited settings, and HIV policymakers are concerned with its sustainable affordability since costs affect the overall health care they can provide from their limited available resources (28). To achieve cost-effectiveness of VL monitoring and thereby long-term sustainability of ART programs in SSA, VL-driven differentiated service delivery models have been proposed (5). VL monitoring provides an ideal opportunity for triaging patients into those who need more attention and closer follow-up and those who need less frequent monitoring. Reducing the intensity of monitoring in the patients who do not require enhanced adherence support or a switch to second-line ART – which represents the vast majority of patients - would substantially reduce the workload and costs of health care facilities.

Further, a reduction in the frequency of clinic visits could improve patients' long-term engagement in care, since reasons for defaulting care include inability to keep up with the intensive clinic visit schedule due to travel time and cost, and loss of work time (7). Evidence suggests that a reduction of clinical engagement in the context of differentiated service delivery does not impact clinical outcomes (4). Time and resources saved in stable patients would allow focusing on those patients with elevated VLs, potentially improving their clinical and virolocococogic outcome through intensified adherence support, clinical follow-up and timely switches to second-line ART. The concept of the proposed automated differentiated service delivery model (aDSDM) is to use VL results to automatically triage participants into groups requiring different levels of attention and care. The

DocuSign Envelope ID: 94DBBACD-5D19-4B54-B740-1BBD7E9B6CE6

implemented aDSDM approach will use a range of eHealth options to support participants' health, adherence to ART and retention in care. The proposed aDSDM is designed to have the potential for being scaled up at national and regional level as it mainly builds on automated triage and communication with patients and health care workers, thus not requiring additional human resources.

4.3. The HIV epidemic in Lesotho and the setting of the VITAL trial

Lesotho is a small landlocked country, surrounded by South Africa, and has an adult HIV prevalence of 23.6%, corresponding to the second-highest HIV prevalence worldwide (29). About 70% of Lesotho's population lives in rural mountainous areas characterised by widespread poverty, poor transport infrastructure and hard-to-reach villages. These geographic characteristics impose an extra impediment on the HIV/AIDS epidemic in Lesotho and access to the health care facilities remains a major barrier for engagement in care.
The VITAL trial will be conducted in the districts of Butha-Buthe and Mokhotlong, in northern Lesotho, from where 18 nurse-led health facilities will participate. Both districts, Butha-Buthe and Mokhotlong, are characterised by mostly rural settings with an estimated population of 220,000, mainly subsistence farmers and mine workers as well as construction or domestic labourers who work in neighbouring South Africa. Each district has only one single mid-size town: Butha-Buthe with ca. 25,000 inhabitants, and Mokhotlong with ca. 10,000 inhabitants. The remaining population lives in villages scattered over a mountainous area of 5,842 km^2. According to the recent household-based national survey from 2016-2017, the adult HIV prevalence is 17.8% in Butha-Buthe and 26.1% in Mokhotlong (30).

5 STUDY OBJECTIVES AND DESIGN

5.1. Hypothesis and primary objective

The primary objective is to assess if the proposed aDSDM is at least non-inferior in terms of clinical outcomes and if it is cost-saving.

Primary hypothesis:
The proposed aDSDM is non-inferior in the proportion of participants engaged in care (defined as attendance of FU visit according to ART supply) and virally suppressed at 24 months follow-up (intention-to-treat (ITT).
Secondary hypotheses:

- The proposed aDSDM is superior in the proportion of participants engaged in care and virally suppressed at 24 months follow-up (ITT)
- The proposed aDSDM is cost-saving (if found non inferior) or cost-effective (if found superior)

5.2. Primary and secondary endpoints

Primary and secondary endpoints of the VITAL trial are listed in *Table 1* below.

**Primary endpoint**	***Hypothesis***
Proportion of participants engaged in care (defined as documented visit attendance) with documented viral suppression (<50 copies/mL) 24 months (16-28 months) after enrollment*	- *Primary hypothesis: The intervention arm is non-inferior (ITT)*<br>- *Secondary hypothesis: The intervention arm is superior (ITT)*

**Secondary endpoints which may become the basis for additional claims**	
1. Proportion of participants with sustained viral suppression (defined as >1 VL <50 copies/mL) during 24 months (16-28 months) follow-up*<br>2. Proportion of participants with sustained viral suppression (defined as >1 VL <1000 copies/mL) during 24 months (16-28 months) follow-up*	*Higher in intervention clusters (ITT and PP)*
**Secondary endpoints indicative of clinical benefit or harm**	
1. Mortality rate at 12 and 24 months after enrollment	*No difference*
2. Proportion of patients with confirmed TB diagnosis at 12 and 24 months after enrollment	*No difference*
3. Disengagement from care at 12 and 24 months after enrollment	*Lower in intervention clusters*
**Secondary endpoints expressing supportive evidence**	
- Time to follow-up VL in case of an unsuppressed VL (≥ 50 copies/mL)	*Shorter in intervention clusters*
- Time to switch of ART regimen in case of virologic failure	*Shorter in intervention clusters*
- Rate of clinic visits at 24 months after enrollment	
- Proportion of participants with ART modification due to virologic failure at 12 and 24 months among participants with virologic failure	*Higher in intervention clusters*<br>*no difference*
- Proportion of participants diagnosed with TB at 12 and 24 months after enrollment	*no difference*
- Proportion of participants receiving a course of TPT	*higher in intervention clusters*
- Proportion of females screened for cervical cancer according to national guidelines	*higher in intervention clusters*
In intervention clusters only:<br>- Proportion of participants requesting a VL result notification through SMS<br>- Proportion of SMS delivered successfully<br>- Proportion of participants using the call-back option through District ART Nurse<br>- Proportion of participants screened positive fo TB by automated call<br>- Proportion of participants appreciating the automated differentiated service delivery model<br>- Proportion of health care providers appreciating the automated differentiated service delivery model	
**Subgroup analyses**	
1. Proportion of participants with viral re-suppression (<50 copies/mL) 24 months (16-28 months) after enrollment among all participants with an unsuppressed VL (≥ 50 copies/mL) during the first 12 months of follow-up*<br>2. Proportion of participants with viral re-suppression (<1000 copies/mL) 24 months (16-28 months) after enrollment among all participants with an unsuppressed VL (≥ 1000 copies/mL) during the first 12 months of follow-up*	*Higher in intervention clusters (ITT and PP)*
**Sensitivity analyses**	

1. Proportion of participants engaged in care (defined as documented visit attendance) with documented viral suppression (<1000 copies/mL) 24 months (16-28 months) after enrollment*	*Higher in intervention clusters (ITT)*

Table 1. Primary and secondary endpoints as well as subgroup and sensitivity analyses according to hypotheses. *Months will be analyzed as calendar months. *if multiple VLs available in time window, the closest to 24 months after enrollment will be used for analysis.*

The primary endpoint is a combination of engagement in care with viral suppression (<20 c/mL) at 24 months follow-up (16-28 months). Engagment in care is defined as documented visit attendance at 24 months (16-28 months).
For the primary endpoint, multiple hypotheses will be hierarchically tested (non-inferiority in ITT population, superiority in ITT population), but no statistical multiplicity adjustment will be needed because of the closed testing principle (31,32).
If the primary objective has been established, secondary endpoints which may become the basis for additional claims will be evaluated hierarchically (33). Per-protocol (PP) population for seconday endpoints will be defined as ≥12 months of follow-up including at least one VL and consecutive clinic visit (within 2 months after blood draw). Statistically significant effects in these secondary endpoints, which are part of our confirmatory strategy, could be considered for additional claims. Clinically very important secondary endpoints are listed separately and will be evaluated independently of the primary objective. They would require further investigation if significant differences are observed, but the primary objective has not been achieved (33). For the secondary endpoints expressing supportive evidence, no claims are intended and only descriptive statistics will be presented. Thus, no multiple testing procedure is needed. However, these secondary endpoints may provide an additional clinical characterisation of the effect of the tested aDSDM.

5.3. Study design

The VITAL trial is a multicenter cluster-randomised controlled, non-inferiority trial in a resource-limited setting. The clusters (rural nurse-led clinics) will be randomised into control and intervention clusters in a 1:1 allocation. Randomisation will be stratified by district. All clinics fulfilling eligibility criteria (see Inclusion and exclusion criteria) will be included.

5.3.1. Stratification criteria

Randomisation will be stratified by district (Butha-Buthe and Mokhotlong).
Using the VL database of our research consortium (8), baseline clinic-performance – measured as the fraction of patients with a suppressed VL (<20 copies/mL) in 2018 – was considered as a stratification factor. However, it was found that all clinics in Butha-Buthe performed better than all clinics in Mokhotlong (in line with the recent LePHIA report (30)). Clinics in Butha-Buthe had a median fraction of suppressed VLs of 77.5 (IQR= [74.7,80.7]), while Mokhotlong had a median fraction of suppressed VLs of 57.65 (IQR= [56.2,58.8]). Thus, baseline clinic performance was dropped as a stratification factor.
In both districts, clusters will be randomised at a meeting with representatives of all participating clinics. Opaque (black), equally-sized and sealed envelopes containing the group allocation (control or intervention) will be held in a braided basket or typical Lesotho hat and envelopes will drawn by representatives, one after each other according to prespecified randomized sequence. Allocation disclosure and documentation thereof will only happen after all representatives have drawn an envelope at very end of drawing event.

5.4. Study intervention

In intervention clusters, an aDSDM will be implemented. Available literature suggests for differentiated service delivery to succeed, to *differentiate* care for defined groups according to three elements: I. clinical characteristics, II. sub-population; and III. Context (3). For VITAL, the context is homogenous

DocuSign Envelope ID: 94DBBACD-5D19-4B54-B740-1BBD7E9B6CE6

(rural, high prevalence setting, generalised epidemic) and was thus dropped for the differentiation. Instead, participants' and providers' preferences will additionally be considered. Therefore, the following dimensions will determine follow-up in the aDSDM of VITAL *(see Figure 1):*

I. **Clinical characteristics:**

Clinical characteristics will focus on the last viral load result but consider TB (screening) results. Based on the last VL result and TB screening result (if available), the VITALapp will show the suggested procedure to the nurse. A message on VL result will be sent as an SMS to the participant (see VL measurement), and the information will be displayed to the nurse on a list he/she downloads to the study tablet (see Case report form 2: Data downloaded and updated by nurse). The nurse will take herself the last CD4 cell count (if available) in to consideration for the follow-up procedure (not displayed on tablet).

a. VL<50, last TB screening negative: long refill interval (6-12 months)
b. *VL ≥50:* clinic visit as soon as possible, adherence support measures and follow-up VL in 3 months
c. *2 consecutive VL ≥50and currently on first-line ART:* consider switch to second-line ART regimen (the nurse receives an automatically generated second-line request, see Case report form 3: Automatically generated second-line request

II. **Sub-population**

Follow-up will also be tailored to subpopulations:

a. *Women who are pregnant or breastfeeding*: According to guidelines, women who are pregnant or breastfeeding have VL measurements every 3 months. They will get a maximum of 3 months treatment supply and will be informed on antenatal, childbirth, postpartum and paediatric care services.
b. *All women:* According to national guidelines in Lesotho, all women living with HIV should get screened for cervical cancer annualy. Informative text-messages on cervical cancer screeing and screening reminders will be sent (see Additional support). In case of an abnormal cervical cancer screening result, the ART supply will be adapted according to the next follow-up date related to cervical cancer screening. If needed, participants will be referred to *St Charles Mission Hospital Seboche* or another specialized clinic in Lesotho for further management (i.e. conization).

III. **Preference/ feasibility**

Participant preference: Participants may choose the following:

a. *Visit frequency:* Participants may inform the nurse at their consultations about their preference for shorter refill intervals
b. *eHealth support:* Participants may decide if they wish to receive SMS and calls (automated TB screening call and call-backs from a nurse). For SMS they may also specify frequency and for both calls and SMS their preferred days and time.

Provider decision: Health care providers get information from the aDSDM and participant preference. They may, in both directions (asking for more and fewer visits), always over-rule the suggested procedure depending on their clinical judgment as well as other circumstances (co-morbidities, stock-out in drug-supply, etc.)

Importantly, the implemented aDSDM is guided by a secured online database where all participant-information and laboratory results are collected, see Data management.

DocuSign Envelope ID: 94DBBACD-5D19-4B54-B740-1BBD7E9B6CE6

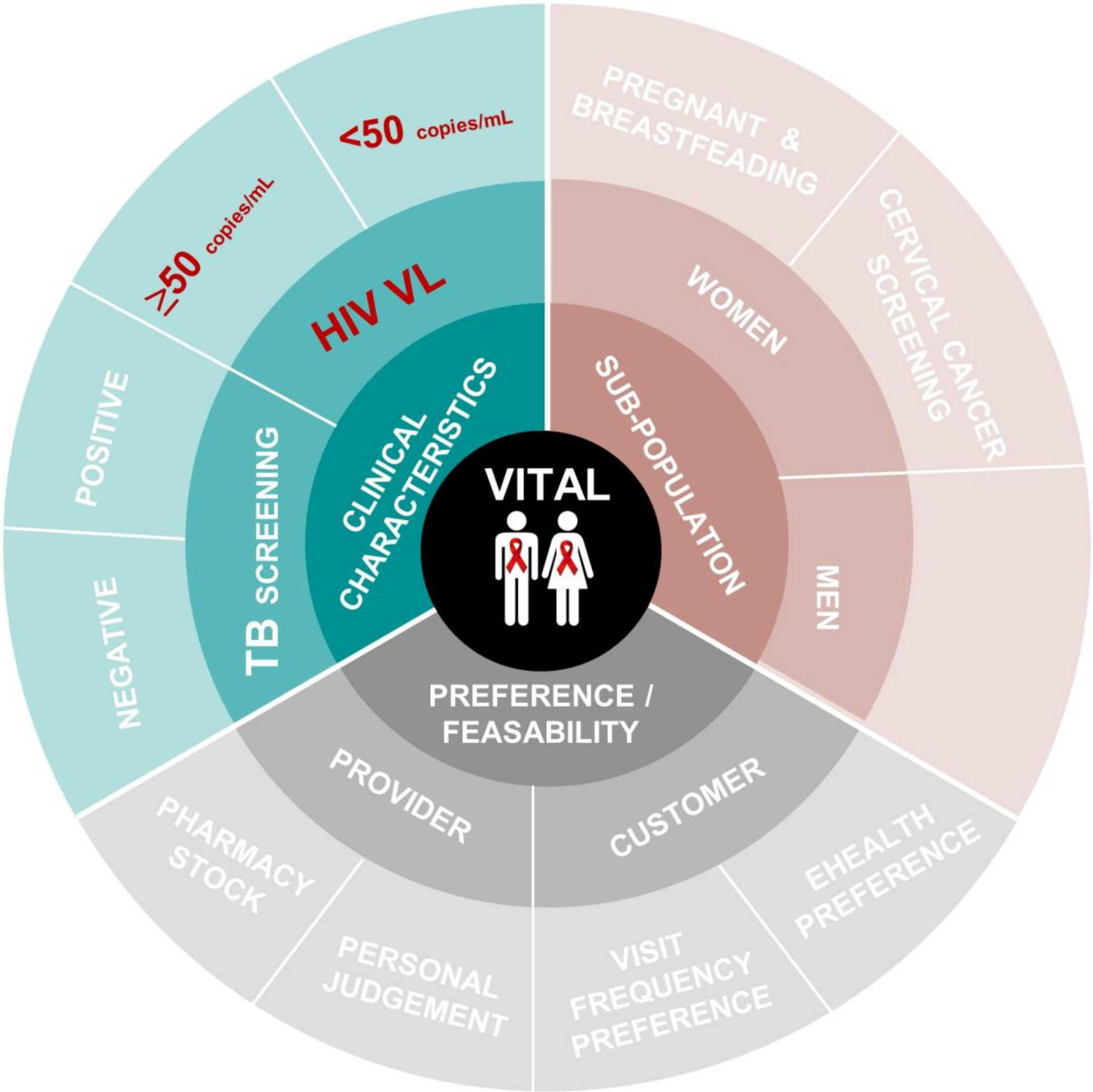

Figure 1. Schematic VITAL overview. *Determinants of follow-up; adapted for VITAL from (2), updated version*

6 STUDY POPULATION AND STUDY PROCEDURES

6.1. Inclusion and exclusion criteria

On an **individual level**, the inclusion criteria for the VITAL trial are the following:

- Taking antiretroviral therapy (independent of viral suppression)
- ≥ 18 years old
- Written informed consent
- intention to remain in the same facility for the duration of the trial as expressed at enrollment
- not enrolled in another study if judged as non-compatible by the (Local) Principal Investigator

On a **cluster level**, inclusion criteria for the VITAL trial are the following:

- nurse-led public or missionary clinic in the districts of Butha-Buthe and Mokhotlong
- consent of clinic management (signed agreement with clinic management)

- access to the internet (internet connection must not be constant, but there must be possibility to down- and upload information daily)
- the clinic sends VL samples to Butha-Buthe laboratory for analysis

Our study population consists of all participants visiting one of 18 study clinics (9 control and 9 intervention clinics) during the enrolment period, fulfilling individual inclusion criteria (see Recruitment, screening and informed consent procedure). We expect to enrol 2655 individuals per arm. For the statistical justification of our sample size, see Sample size.

6.2. Data management

Data management is essential to the VITAL trial and will be important for the subsequent description of enrolment and study procedures.
The data management of the VITAL trial will be guided by an existing VL database of the research consortium (Study entitled "Implementation of routine viral load monitoring in rural Lesotho: A prospective cohort-study on virologic outcomes among patients on antiretroviral therapy in Lesotho" (ID-134-2016)). A connected VITAL app will be developed to connect study tablets at the clinics (intervention and control clusters) with the VL database. All participant data collected on these tablets at clinics will be uploaded to the VL database. Further, all laboratory test results requested for VITAL participants (VL measurements and TB tests) will be uploaded into the VL database through the Laboratory Information System (LIS). The VL database will guide the triaging of participants according to uploaded laboratory results. eHealth support, information and screening will be implemented and directly delivered from the VL database.
At each participating clinic (intervention and control clusters), there will be two study tablets: One used by a VITAL records assistant (VRA) and one by the nurse. Participants attending a clinic visit can be searched on the VITAL application (app) installed on theses tablets using their ART-number. Depending on the user (VRA or nurse), the VITAL application will display the following information/interface:
VRA user:

- Recruitment including documentation of informed consent
- Enrolment data collection
- Documentation of pharmacy refills (Daily review and entry of pharmacy documentation)
- Documentation of participant tracing

Nurse user:

- Documentation and update of participants data
- Reception of recommended actions and documentation of actions taken
- second-line regimen request forms

Further, the VL database will entail two secured platforms: one where the second-line committee may review second-line requests and an one where the district ART nurse may manage phone requests.

6.3. Recruitment, screening and informed consent procedure

Participants will be recruited at clinics by well-trained VRAs using a study tablet (one VRA per clinic). VITAL will enrol participants for 6-12 months (expecting to enrol all participants regularly visiting a clinic during this time). The VRAs will explain to each participant the nature of the study, its purpose, the procedures involved, the expected duration, the potential risks and benefits and any discomfort it may entail. Each participant will be informed that the participation in the study is voluntary and that he or she may withdraw from the study at any time and that withdrawal of consent will not affect his or her subsequent medical assistance and treatment. The participant will be informed that his or her medical records may be examined by authorised individuals other than their treating nurse either on-site or remotely.
All study participants of the study will be provided with a participant information sheet and a consent form describing the study and providing sufficient information for participants to make an informed decision about their participation in the study. Both sheets will be provided in the local language, Sesotho, and the participant will receive a copy of the consent form in written format to take home.

Participants have the right to withdraw at any time without giving reasons. In case of withdrawal, only data collected until the time of withdrawal will be used for research purposes (in an encoded manner). The formal consent of a participant, using the approved consent form, will be obtained before the participant is submitted to any study procedure. Illiterate participants will provide a thumb-print and a witness (independent to the trial and > 21 years old), chosen by the participant, will co-sign the form. The consent form will be co-signed and dated by the VRA at the same time as the participant or participants' witness sign. A copy of the signed informed consent will be given to the study participant. The consent form will be retained as part of the study records, and the VRA will immediately use the study tablet to photograph the consent form and attach it to the enrollment data of the participant.

6.4. Study procedures

6.4.1. Study duration

The VITAL trial was launched on 07.09.2020 and will continue until 31.07.2024 (excluding preparation and analysis). The enrolment period of the trial will be 6-12 months. If minimum sample-size is not reached during this period, recruitment may be extended to six months. We are expecting to enrol all participants regularly visiting a clinic during this time. All participants will be followed-up for 24 months (16-28 months). Thus, the anticipated total duration of this trial will be **47 months.** In both study arms, VL measurement dates will be according to guidelines and participants' previous VL results. In intervention clusters, follow-up visit dates will be determined by the aDSDM. In control clusters, follow-up visit dates will be according to the standard of care (SOC).

6.4.2. Enrolment clinic visit

In both control and intervention clusters, directly after recruitment and informed consent procedure, the VRA will collect enrolment data of all participants (see Case report form 1: Variables collected at enrolment). Enrolment data will contain variables of the following categories:

- General participant information
- Sup-population specific health-related information
- VL database update
- Adherence information
- eHealth information (in intervention clusters only)
- Mental health questionnaire (Appendix 9)

These variables will not be updated at each visit. However, variables can be updated if participant information changes.

6.4.3. Follow-up study procedures

Figure 2 displays an overview of the follow-up procedures of the VITAL intervention. An aDSDM containing all components of the ART care package will be implemented in intervention clusters containing long-term ART supply, VL text-messages, preference-based EAC, remote TB screening and additional support. The VITAL trial will not interfere with the SOxC VL monitoring schedule. After VL measurement, participants will obtain ART for 1 month and will be asked to return to the clinic 1 month thereafter. Further, for both arms, VL ≥50 copies/mL will trigger an ART Advisory Committee notification for guidance, and possible regimen switch to second or third-line therapy, respectively. Further, in both arms, the VITAL app installed on study tablets will be used for enrollment and documentation of follow-up procedures. Apart from this, VITAL will not interfere with SOC in control clusters. **The follow-up study procedures described below, refer to intervention clusters.**

	VL < 50		VL ≥ 50		no VL	
	Control	Intervention	Control	Intervention	Control	Intervention
VL measurement* (6 months after ART start, thereafter…)	VL every 12 months	• VL every 12 months<br>• result & long-term supply information by SMS	repeat VL in 3 months	• repeat VL in 3 months<br>• result & EAC information by SMS	VL at next clinic visit	• clinic visit reminder<br>• VL at next clinic visit<br>• result by SMS
ART supply* (months)	1-6	1-12	1-3	1-3	1-6	1-6
EAC – What			standard EAC	≥1 of the following:<br>i. standard EAC<br>ii. EAC on phone<br>iii. intake reminder SMS		
EAC – When (time after VL)			4-8-12 weeks	i. 4-8-12 weeks<br>ii. preference-based<br>iii. preference-based		
TB screen – What	standard	• Standard<br>• automated phone call	standard	standard	standard	standard
TB screen – When	at every clinic visit	• at every clinic visit<br>• on phone every 3 months**	at every clinic visit	at every clinic visit	at every clinic visit	at every clinic visit
TB prophylaxis supply*	1-6	1-6 (same as ART supply)	1-3	1-6	1-6	1-6
additional support		• call-back from DAN<br>• intake/visit reminders<br>• health SMS		• call-back from DAN<br>• intake/visit reminders<br>• health SMS		• call-back from DAN<br>• intake/visit reminders<br>• health SMS
AAC notification			after 2 VL ≥50 despite EAC	• after 2 VL ≥50 despite EAC<br>• Simplified AAC notification procedure***		
cervical cancer screening	bi-annually	• Bi-annually<br>• ReminderSMS<br>• Clinical recommendation	bi-annually	• Bi-annually<br>• Reminder SMS<br>• Clinical recommendation	bi-annually	• Bi-annually<br>• Reminder SMS<br>• Clinical recommendation

VL = viral load, TB = tuberculosis, ART = antiretroviral therapy, EAC = enhanced adherence counselling, DAN = VITAL district ART nurse, AAC = ART advisory committee
* >3 months supply in intervention only given if last TB screening negative, not pregnant or breastfeeding and not conflicting with the repetition of an abnormal cervical cancer screening result
** if long-term supply > 3 months is provided
*** if second VL≥1000 copies/mL suggestion to consider genotypic resistance testing (GRT)

Updated in January 2023 to align with the Sixth Edition of National guidelines on the use of antiretroviral therapy for HIV prevention and treatment (January 2022)

Figure 2. HIV care package and according VITAL intervention. *Red indicates main differences to standard of care, updated version*

VL measurement

VL measurements will be done following the individual schedule, according to guidelines. In intervention clusters, SMS informing participants on clinic appointments and VL results, and further procedures will be offered.
The exact wording of messages/message options were determined together with health care professionals in Lesotho. All messages will be translated into Sesotho. In order to be informative but non-disclosing, messages will not include words like *HIV, ART, treatment, lab, blood draw* or similar. However, VL result will be displayed as a blank number and the message will end with *Greetings from the respective clinic*. In intervention clinics, an educational poster will be put up to explain the meaning of the respective VL-result SMS.

One month before VL due date, participants receive a SMS reminder:
Please remember to go to your test appointment in one month. Greetings, [clinic name]

One week before VL due date, participants receive a second SMS reminder:
Please remember to go to your test appointment in one week. Greetings, [clinic name]

After VL measurement, participants receive an automatically generated SMS according to their VL result:

DocuSign Envelope ID: 94DBBACD-5D19-4B54-B740-1BBD7E9B6CE6

**VL < 50**	**VL ≥ 50**	**Technical failure of VL measurement**
Congratulations on good test ([result])! Keep it up! Discuss long-term supply! Greetings, [clinic name]	**a. 1st VL ≥ 50**<br>*Your test ([result]) is out, please come and see us as soon as possible to discuss support options. Greetings, [clinic name]*<br>**b. 2nd VL ≥ 50**<br>*Your test ([result]) is out, please come and see us as soon as possible to discuss a switch. Greetings, [clinic name]*	*Your test should be repeated, please come back as soon as possible, sorry for inconvenience. Greetings, [clinic name]*

Medication supply

ART, Tuberculosis Preventive Therapy (TPT) and TB treatment supply (for non-resistant TB cases) will be provided at clinic visits. TPT will be given the same as ART supply (with maximum of 6 months TPT supply). The amount of medication supply will be determined by clinical characteristics (with focus on VL suppression), subpopulation and participants' and providers' preference and feasibility (see *Figure 1* and *Figure 2*). More than 3 months supply will only be provided if the last VL< 50copies/mL if the last TB screening was negative, if the participant is not pregnant or breastfeeding and if no repetition of an abnormal cervical cancer screening result is indicated at an earlier time-point.

The nurse regularly downloads electronic participant lists to his/her study tablet, which were automatically generated by the VL database (see Case report form 2: Data downloaded and updated by nurse). Participants attending a clinic visit can be searched on this list using their ART-number. The list can be updated offline and uploaded to the VL database later (once online).

The list contains the following clinical information:

- Last 2 VL results and due date of next VL
- Remote TB screening result (if available)
- TB Xpert Result (if available)
- Previous TPT information
- Next Cervical Cancer Screening date

Further, the list contains information on contact with the district ART nurse (DAN), if applicable and the following information on recommended action regarding ART and TPT.

Recommended ART (according to VL):

**VL < 50**	**VL ≥ 50**
No special action / consider long-term supply to [date of next VL]	**a. 1st VL ≥ 50**<br>*Preference based EAC and next VL [month/year that is 3 months after 1st VL].*<br><br>**b. 2nd VL ≥ 50**<br><br>*Continue EAC, consult AAC.*<br><br>**c. 2nd VL ≥ 1000**<br>*Consult AAC for switch to effective regimen, consider GRT*

**Technical failure of VL measurement**	**Last VL result more than 12 months ago**	**Last VL result ≥ 50 and more than 3 months ago**
Repeat VL today or as soon as possible.	*Do VL today or as soon as possible.*	*Repeat VL today or as soon as possible.*

Recommended action TPT (TB screening done regardless of distant screening result):

**Participant on TPT**	**Participant not on TPT**
Distant TB screening was [positive/negative (date of screening), due to the following symptom: […]<br><br>*Today: Screen for TB symptoms: cough, fevers, night sweats, weight loss. Compare chart weights from previous visit and today.*<br><br>- *If screening positive, stop TPT, provide sputum bottles and include ART number on TB request form.*<br>- *If screening negative and course of TPT not finished, continue TPT.*<br>- *If screening negative and course of TPT is finished, discontinue TPT.*<br>- *If a recent Xpert result was positive, stop TPT (if not stopped yet), start TB treatment independent of symptomatic screening.*	*Distant TB screening was [positive/negative (date of screening), due to the following symptom: […]*<br><br>*Today: Screen for TB symptoms: cough, fevers, night sweats, weight loss. Compare chart weights from previous visit and today.*<br><br>- *If screening positive, provide sputum bottles and include ART number on TB request form.*<br>- *If screening positive, but a recent Xpert was negative, more clinical evaluation is needed. Consider referring to hospital.*<br>- *If screening negative, and participant has never had TPT, give TPT.*<br>- *If screening negative, and participant has had TPT before, no action needed.*<br>- *If screening negative and TPT was interrupted within the last 3 months due to a positive TB screening, but following Xpert result was negative, continue course of TPT.*<br>- *If screening negative and TPT was interrupted more than 3 months ago due to a positive TB screening, but following Xpert result was negative, start a new full course of TPT.*<br>- *If a recent Xpert result was positive, start TB treatment independent of symptomatic screening.*

DocuSign Envelope ID: 94DBBACD-5D19-4B54-B740-1BBD7E9B6CE6

The nurse will be asked to fill in variables about the participants' visit by the VITAL app on her tablet. Variables filled in by the nurse cover the following categories:

- ART action taken (ART prescribed, ART supply, information on next VL)
- Participants EAC preference (if applicable)
- Participants adherence (if receiving EAC)
- TPT/TB action taken
- Cervical cancer screening
- Next visit date, according to the following recommendations:

Next visit date

**VL <50**	**VL ≥ 50**
Nurse may enter 1-12 months and specify if less (i.e. participant wish, other medical condition, pregnancy)	**a. 1st VL ≥ 50**<br>*1-3 months according to preference based EAC*

Pharmacy dispense documentation

Depending on the clinic, ART, TPT and TB treatment are given out by the nurse directly or at the pharmacy. In both cases,the VRA responsible for participant enrolment will document pharmacy dispenses during the entire VITAL trial using a study tablet. The number of ART, TPT and TB treatment pills dispensed to each participant will be documented (see Case report form 4: Pharmacy dispense and tracing list generated).

Tracing list generated

Using the pharmacy dispense documentation, a list of those participants who are late for a refill for over two months will automatically be created and provided as a tracing list to the facility. Notifications will appear on the study tablet for the VRA user. Both, the VRA and the nurse, will be able to access a full list of patients who need to be traced on their study tablet. However, it will be the VRA's responsibility to do the tracing (call the patient on all available phone numbers, then call village health worker), to discuss it with the nurse and record tracing results (see Case report form 4: Pharmacy dispense and tracing list generated).

Enhanced adherence counselling

Following SOC, a VL ≥1'000 copies/mL triggers EAC. In SOC, EAC is provided around 4, 8 and 12 weeks after the VL phlebotomy at the clinic. In the VITAL intervention, EAC is according to participant preference. At the nurse visit after the VL measurement, participants may choose among the following EAC options (choice of more than one possible, see Case report form 2: Data downloaded and updated by nurse):

i. Standard SOC EAC
ii. On the phone with a district ART nurse (DAN)
iii. ART intake reminders

ii. EAC on the phone with DAN

An online list is generated for the DAN as an overview of who needs a call-back for EAC, and he/she can enter information from this call into the database that then appears on the tablet of the clinic nurse.

Variables in this list are among the following categories (see Case report form 5: Data from District ART nurse):

- Call specific information
- Participant's adherence information

To compare self-reporting of adherence on the phone and in person, adherence information provided to DAN will not be displayed to the nurse at the clinic and vice-versa.

iii. ART intake reminders

Participants choosing ART intake reminders as (one of) their preferred adherence support measures may at the same time specify frequency and text of these reminders (see Case report form 2: Data downloaded and updated by nurse).

Remote TB screening

Participants accepting an ART supply of over 3 months will receive an automated remote TB screening phone call 3 months after their clinic visit and every 3 months thereafter. The phone call will be at their preferred day and time (defined at enrollment) and screen participants for TB according to the WHO recommended symptom screening. They will be asked about the presence of each of the following symptoms and asked to answer by dialling *1 = yes, 2 = no*, *see Figure 3*.

- Current cough
- Fever
- Night sweats
- Weight loss

A positive screening result will automatically trigger an SMS inviting the participant to the clinic. An unanswered call will be repeated 3 times, with gaps of one day, before being marked as failed. If two phone numbers of a participant are known, the first two TB screening calls will go to the first phone number and the two consecutive calls to the second phone number. For unreachable participants, a separate list will be generated and the district ART nurse will call the relatives of the participant *("Whom can we contact if we don't reach you?")*.

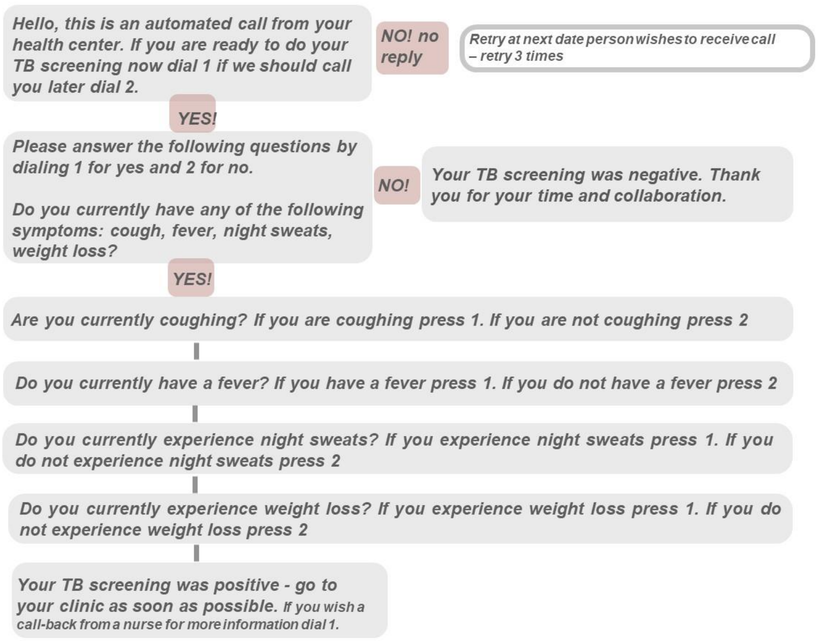

Figure 3. Remote TB screening algorithm, updated version.

ART Advisory committee notification

As per national guidelines (sixth edition, January 2022),the ART advisory committee is notified in case of virologic failure (defined as two consecutive VL ≥ 50 copies/mL). In VITAL intervention clusters, this second-line request is automatically generated by the VL database if the second VL above 50 is uploaded. It appears on a list on the tablet of the nurse for confirmation and submission to the ART Advisory Committee through the nurse. The ART advisory committee notification contains variables of the following categories (see Case report form 3: Automatically generated second-line request):

- Clinical information, current and history (on HIV and TB)
- Preference-based EAC information

The nurse should not wait for the next clinic visit of the participant to submit the ART advisory committee notification; ideally, until then the nurse will have received feedback from the committee, and can switch the treatment regimen if recommended.

If the alert appears on the tablet, the nurse can take the following actions:

- Confirm that provided information (participants information, VL history) is correct
- Add/correct ART history and clinical history
- Add remarks (free text) (i.e. adherence)
- Submit
- Decline notification
- Name and contact of a nurse (phone)

The full notification is then sent to the ART Advisory Committee. The ART Advisory committee can review the full second-line request online and add their decision:

- Switch approved/declined
- Recommended regimen
- Remarks
- The committee who approved the second-line response
- Date of decision

The full request with response is sent to the tablet of the responsible nurse. This shall happen before the participant's visit to discuss VL results (4 weeks after blood draw) so that on that visit a potential switch may already take place.

Additional support

Additional support and education measures are offered in the VITAL intervention clinics.

i. Call-back from DAN

At enrollment, participants will be informed on their option of requesting a call-back from a nurse by sending the text-message "call-back" or by leaving a missed phone call (phone number distributed at enrollment). The DAN will call back and enter the following information to a list.

- Call information
- Reason for call-back request

ii. ART intake reminders

Participants not receiving EAC may also request ART intake reminders at each clinic visit. They may specify frequency and text of these reminders (*see Appendix 2*).

iii. Educational posters at the clinic

In collaboration with health care professionals in Lesotho, posters will be generated and put up at all intervention clinics during the VITAL trial. These posters will serve to explain the meaning of the text messages and all other eHealth support in detail.

iv. Subpopulation specific health messages

DocuSign Envelope ID: 94DBBACD-5D19-4B54-B740-1BBD7E9B6CE6

6.5. Women: *“Your cervical cancer screening is due – please remember to get this done and stay healthy. Greetings, [clinic name]”* Withdrawal and discontinuation

A participant may be withdrawn from the study upon withdrawal of informed consent. In that case, only data of participants up to the point of withdrawl of consent will be analyzed. A cluster may be withdrawn from the study upon withdrawal of clinic management consent. Premature cluster withdrawal will be reported. On the cluster level, only data up to the point of withdrawl will be analyzed. Participants who are lost to follow-up during the VITAL trial will still be analysed as part of the ITT population and will be considered as not having reached the composite primary endpoint. Participants who are lost to follow-up during the VITAL trial after a follow-up of over one year, including at least one VL measurement during VITAL and subsequent attendance at the clinic, will be considered for the PP population analyzed for secondary endpoints. Participants who miss their scheduled clinic visit will be traced using the automatically generated tracing list.

7 STATISTICS AND METHODOLOGY

7.1. Statistical analysis plan and sample size calculation

7.1.1. Design

The non-inferiority design only applies to the primary endpoint (engagement in care with documented viral suppression). This design choice was made because an aDSDM should likely be the standard of care in resource-limited settings if it does not negatively affect participants’ health outcomes as compared to standard of care. Two envisaged benefits of an aDSDM approach are a reduction in: (1) participant volume at healthcare facilities, which will allow healthcare workers to focus on those participants who are not doing well, potentially improving their clinical and virologic outcomes; and (2) the substantial time and financial burden (transport costs) on participants of having to attend a clinic.

7.1.2. Non-inferiority margin

Based on consultations with experts, we chose a margin of non-inferiority (NI) for the odds ratio (OR) of reaching our primary endpoint of engagement in care with documented viral suppression of 0.8. That is, if the OR of the primary endpoint in the intervention group compared to the control group is statistically significantly higher than 0.8, then the implemented aDSDM approach is considered to be non-inferior to standard of care. On an absolute scale, this non-inferiority margin corresponds to a higher absolute probability of failing to reach the primary endpoint of 5% in the intervention compared to the control group, assuming that 65% per cent of participants in the control arm will be engaged in care with documented viral suppression at the end of the follow-up period. Compared to literature on non-inferiority or equivalence trials in similar settings, our NI margin was set conservatively. Both, Jaffer and colleagues (35) for their equivalence cluster randomized trial on ART home delivery in rural Uganda and Geldsetzer and colleagues (36) for their non-inferiority cluster randomized trial on community ART delivery in Dar es Salaam, Tanzania, choose a NI margin that reflected a lower absolute probability of viral suppression in the intervention group of 9%, assuming that 80% of participants in the control arm did reach viral suppression at the end of the follow-up period (corresponding to a NI margin for the OR for viral suppression of 0.61).

7.1.3. Sample size

We calculated the sample size for the non-inferiority cluster randomised design according to (37–39), which corresponds to an individually randomized design multiplied by a design effect. We used odds ratios (OR) to assess the association between intervention or control allocation and our binary primary endpoint. An OR of 1 corresponds to no relative effect, an OR >1 to a positive relative and an OR <1 to a negative relative effect in the intervention versus control clusters. We aim at testing the null hypothesis H_0: OR ≤ NI versus H_A: OR > NI, where NI represents the prespecified level of non-inferiority (0.8) and the rejection of the null hypothesis implies non-inferiority.

We used the VL database of the research consortium (8) to obtain viral suppression data of the study clinics in 2018. We estimated the percentage of engagement in care with an HIV viral suppression of individuals on ART for over 6 months in Lesotho to be 65%. We hypothesise that engagement in care with viral suppression in the aDSDM intervention group will be 10% higher than in the control group. We used the same VL database to calculate an intra-cluster correlation coefficient (ICC). We fitted a linear mixed-effects model (40) to the last VL (log10) of each patient, corrected for sex and the first CD4 cell count (per 200) and included the respective clinics as random effects. Unfortunantly, two pairs of clinic could not be distinguished due to ambiguous abbreviations. We estimated an ICC of 0.04 from the model fit, as the proportion of the variance explained by the cluster structure in our population. According to (41), we included the mean cluster size and standard deviation to our design effect, which we estimated as the number of routine VL measurement performed in 2018 at our study clinics and the according standard deviation (mean cluster size of 346.7, standard deviation of 127.5). Our desired type I error rate was chosen as 2.5%. With a minimum of 8 clusters (corresponding to 2655 participants) in the intervention group and a minimum of 8 clusters in the control group, we will be well powered (>80% power) to detect one-sided differences of >10% in the proportion of participants who were engaged in care and virally suppressed in the intervention compared to the control arm.
We did not correct our sample size calculations for our stratified design, which will decrease the ICC and thereby our sample size, but will rather use this a security margin.

7.1.4. **Analyses of clinical outcome**

For the primary endpoint, participants will be analysed according to their cluster allocation arm (intention-to-treat (ITT)). Baseline participants' characteristics and intervention-cluster specific outcomes will be summarized as frequency and percentage with 95% confidence interval, or median with interquartile ranges.
The primary analysis will use a logistic multivariable mixed-effects regression model to assess the difference between engagement in care with documented viral suppression 24 (16-28) months after enrollment in the intervention versus control arm, where cluster variation is accounted for as a random effect. The analysis choice was made due to the high efficacy of mixed-effects regression models for highly variable cluster sizes. This analysis will be adjusted for the following set of covariables:

- Study arm allocation (fixed effect)
- District (the pre-specified randomisation stratification factor, fixed effect)
- Clustering according to clinics (clusters, mixed effect)
- Other Baseline factors strongly prognostic of the primary outcome (defined in statistical analysis plan)

Non-inferiority of the intervention arm will be concluded if the lower bound of the 95% CI of the OR of intervention is superior to 0.8 (non-inferiority margin). Non-inferiority and superiority will be concluded if the OR is superior to one and the 95% CI does not include 1. For all covariables, univariable and multivariable regression coefficients will be presented.

Participants with missing primary endpoint information are considered as a failure to be engaged in care. Missing baseline participants' characteristics will be imputed using multiple imputation techniques in subsequent analyses. Sensitivity analyses will be conducted to provide evidence that the result seen from the primary analysis is robust. These analyses will focus on the deviations in model assumptions and will be detailed in the statistical analysis plan.
Clinically very important secondary endpoints are listed separately, will be evaluated independently of the primary objective and would require further investigation if significant differences were observed, but the primary objective was not been achieved.
As for the primary endpoint analysis, estimated OR from a mixed-effect logistic regression for intervention will be reported.

Continuous secondary endpoints will be reported as mean with 95% Wald CI for each treatment arm, as well as difference in means between participants allocated to the standard or care and participants allocated to the intervention arm.
For all tests, we will use 2-sided p-values with alpha 0.05 level of significance.
A detailed statistical analysis plan will be developed before the last participant completes follow-up and will be added to the study protocol at clinicaltrials.gov. Deviations from the statistical plan will be described and justified in the final trial report. Results will be reported according to the CONSORT guidelines for cluster-randomized trials (42) and study findings will be submitted for peer-review in open-access journal. Study authorship will follow International Committee of Medical Journal Editors recommendations and we do not intend to use professional writers.

Subgroup and sensitivity analysis: We will restrict our trial population to participants with unsuppressed VL (≥ 20 copies/mL) during the first 12 months of follow-up and analyse 1) the proportion of participants with viral re-suppression (<20 copies/mL) 24 months (16-28 months) after enrolment and 2) the proportion with sustained VL suppression (defined as >1 VL <20 copies/mL) during 24 months (16-28 months) follow-up. Robustness to the VL threshold at 20 copies/mL will be addressed in sensitivity analyses where the threshold will be increased to 1000 copies/mL. The rationale for modifying the VL threshold is that some clinics might shift to point-of-care VL measurement or VL measurement from dry blood spot and these approaches have a higher threshold of detection.

7.1.5. Cost-effectiveness or budget-impact analysis

We will analyze cost-effectiveness if our intervention is found superior or budget-impact if our intervention is found non-inferior with a time frame of three years (corresponding to the study period). The following data will be collected: (1) estimating the cost of the intervention; and (2) the number of HIV-positive persons achieving the primary endpoint (engagement in care along with viral suppression) through the intervention, which will be derived from the study.
Cost data will include: (1) Conversion rate of local currency to U.S. dollars at 6-month intervals over the life of the project; (2) costs of all commodities used in the intervention; (3) community sensitization, promotional and advertising costs; (4) average time clients spent with intervention including transportation / staff time / time for referrals; (5) local wages of target population; (6) incentives to clinics; (7) other relevant costs, including training of providers and transport costs; and (8) time and motion studies will be conducted to determine the time and resources necessary to provide the intervention. Time and motion studies will be conducted over a two to four week period at two random sites during enrollment and again when the intervention is running at full capacity. An experienced research assistant will collect data on time required to complete each step of the intervention. Results from initial time and motions studies will be shared with the teams and strategies for efficiency shared to reduce the time needed for the intervention. Observing multiple visits will allow estimation of the average time taken for each step; the time taken for research purposes (e.g. data collection) will be noted separately from the estimated time needed for clinical services. Interviews with study staff will also quantify the effort required for each step of the intervention.
A discount rate of 3% will be used with sensitivity analysis of 0% to 5%. Additionally, for analysis taking the societal perspective, we shall also include the cost to clients in terms of lost time, wages, childcare, and other relevant opportunity costs. To assess these issues, we will ask participants what expenses and opportunity costs they incurred to receive the interventions in the surveys already planned for the project. We will also collect data on the average cost of medical care in Lesotho associated with HIV infection and AIDS through literature review.
Cost data will be used with study results and mathematical models of HIV transmission to estimate the population level effectiveness and cost-effectiveness of the intervention. The models will use de-identified study data.

7.2. Handling of missing data and drop-outs

DocuSign Envelope ID: 94DBBACD-5D19-4B54-B740-1BBD7E9B6CE6

Our main analysis will be an intention to treat analysis. Participants with missing clinical outcomes will be dropped from the respective clinical assessments, with the exception of the primary endpoint for which missing information are considered as a failure to be engaged in care. Participants' characteristics of the dropped population will be compared to patient characteristics of the trial participants that have an outcome to avoid further bias. Missing baseline participants' characteristics will be imputed using multiple imputation techniques in subsequent analyses.

8 NESTED STUDIES

The VITAL trial provides the ideal setting for further nested studies.

8.1. aDSDM for Tuberculosis prevention and care

8.1.1. Background

TB is the most common cause of mortality among PLHIV in sub-Saharan Africa. Up to a third of active TB disease goes undiagnosed and uptake of TB preventive therapy (TPT) for PLHIV has been below targets, despite demonstrated disease prevention and mortality benefits. There is limited evidence as to how differentiated care for HIV will affect TB outcomes.

8.1.2. Methods

The aDSDM intervention model proposed for VL-driven HIV care provides an opportunity to assess a model for integrated TB services as well. Automated TPT treatment recommendations on the nurse's tablet may facilitate dispensing of TPT. Multi-month dispensing of TPT will be coordinated with ART dispensing. Decreased in-clinic TB screening opportunities for participants receiving multi-month scripting for ART will be augmented with electronic symptom screens. Using routinely available laboratory data (GeneXpert TB test results), we will compare TB incidence in the control and intervention arms. Using pharmacy dispensing data, we will assess the proportion of persons in each group who receive a course of TPT, and who receive TB treatment.

8.1.3. Discussion

We hypothesize that a greater proportion of persons in the intervention arm will receive a course of TPT during the study period. We hypothesize that there will a higher proportion of intervention arm participants diagnosed with active TB by month 12, but a lower proportion of intervention arm participants diagnosed with active TB by month 24.

8.2. Integrating mental health assessment for optimized patient outcomes

8.2.1. Background

As HIV infected people on ART live longer and age, comorbidities like mental health gain importance and are associated with HIV care outcomes.There is a potential threat to achievement of the 90-90-90 targets if mental health is not addressed as this affects every step of the care cascade, including ART adherence and viral suppression, which is relevant to the focus of the parent study. However, data on common mental disorders (CMDs) among PLHIV in Lesotho are very scarce. Objectives of this nested study include: (i) assess the burden of CMDs among adult PLHIV in Lesotho, (ii) establish the association between CMDs and clinical and virologic outcomes in this population, (iii) assess the impact of study treatment (i.e., automated differentiated service model using VL results) on burden of CMDs at 12 and 24 months follow-up and (iv) produce a psychometrically valid brief assessment tool for CMDs that can be administered by lay providers.

8.2.2. Methods

VITAL participants enrolled in both intervention and standard of care clinics will be screened for CMDs using four screener test tools that have been validated in similar settings, but not yet in Lesotho. The

nested study will focus on the following CMDs in this setting: major depression, generalized anxiety disorder (GAD), posttraumatic stress disorder (PTSD), and problematic substance use (alcohol and illcit drugs) using the Patient Health Questionnaire-9 (PHQ-9), Generalised Anxiety Disorder-7 (GAD-7), Primary Care Post Traumatic Stress Disorder (PC PTSD), the Alcohol Use Disorders Identification Test-Concise (AUDIT-C) and one item asking about weekly illicit drug use, respectively. All screening tools will be translated into Sesotho. Trained VRAs will administer the translated tools to a random subsample of VITAL participants at baseline (at enrolment into main VITAL study) and at the 12- and 24-month follow-up visits. For the 12-month follow-up visit, participants may attend during the 8-16 month window and for the 24-month follow-up visit, participants may attend during the 16-28 month window.

The estimated prevalence of CMDs will be assessed using cross-sectional data collected at enrollment visit into VITAL. Any effect of sociodemographic determinants (age group, gender, education status, employment status, marital status) and clinical factors (ART regimen, WHO stage, VL suppression) will be assessed during descriptive analysis. In order to reduce risk of harm to participants completing the screener tests, all patients with suicidal thoughts (i.e those with score 1 or above on question 9 on the PHQ-9, which assesses thoughts that one would be better off dead or of hurting oneself in some way), the participant will be subsequently assessed by a trained clinic nurse in a suicide risk assessment protocol. All staff will follow the referral process protocol for suicide risk assessment (Appendix 1) and the trained clinic nurse will follow the suicide assessment procedures (Appendix 2). Briefly, the suicide risk assessment tool assesses four primary aspects of suicide risk: suicidal intention, suicide plan, access to suicidal means, and reasons for living. After conducting the suicide risk assessment, the nurse will receive a phone consultation by a trained mental health provider or doctor to determine appropriate course of action. Participants demed to be at imminent risk of suicide will be taken to the local hospital for further observation and assessment. Participants deemed to be at moderate risk will be scheduled with the local mental health provider for clinical follow-up (e.g., medication or therapy). For participants deemed to be low risk, a passive referral for mental health follow-up will be provided. These risk assessment procedures have been instituted from the beginning of the study but were not previously written into the protocol.

Participants screened for CMDs at enrollment/baseline will be followed up at 12 and 24 months after baseline to determine any association between identified CMDs and clinical and virologic outcomes. Analysis of this follow up data will allow for description of any longitudinal trends.

To assess whether there is any effect of the primary study intervention (automated differentiated service model using VL results to triage patients on CMDs), the crude and adjusted (i.e., adjusted for relevant demographic and clinical factors) prevalence of the CMDs will be compared over time between intervention and control clusters/clinics. This analysis will allow for appropriate follow up qualitative studies that will be designed and submitted to the NHREC for ethics approval at a later stage.

To ensure the above CMD asssesment screener tools are valid for routine clinical use in Lesotho, it is necessary to conduct a study on the psychometric properties of the measures. This is planned for a subset of randomly selected 400-500 participants during the 12 month follow up of the VITAL study using field tested and translated Diagnostic Interview for Anxiety, Mood,and Obsessive-compulsive and related Neuropsychiatric Disorders (DIAMOND) tool. A detailed methods will be submitted as an amendment to the ethics committee.

8.2.3. **Discussion**

We expect to estimate the burden of CMDs among HIV patients on ART in Lesotho and generate valuable evidence that can be used to identify appropriate interventions (depending on the distribution of mental health disorders in this population) to better integrate mental health diagnosis and treatment into HIV care in Lesotho. We hypothesize that the estimated burden of CMDs will be lower in VITAL intervention clinics after 12 and 24 months. The differentiated care offered by the intervention that integrates patient preferences on frequency of nurse visits, drug pick ups and mode of EACs would reduce substantial time and financial burden (transport costs), subsequently having positive effects on the patients mental health.

8.3. The ideal VL threshold for participants who should get a 3-month FU VL

8.3.1. Background

There is no clear rationale for a threshold of 1000 copies/mL defining virologic failure (43).

8.3.2. Methods

In both, control and intervention clusters, a standard of care improved by a 3-month follow-up VL for participants with an initial VL 20-999 was implemented until January 2023. Thereafter, follow the revised national guidelines of Lesotho (sixth edition, January 2022) the viral load thresholds was adapted from 1000 copies/mL to <50 copies/mL.

The necessity for the second VL in different strata of participants with VL 20-999 will be assessed retrospectively, aiming at defining a threshold where close VL follow-up measurement should be recommended.

8.3.3. Discussion

We hypothesise that an initial VL greater than 20 is not only a single elevated VL (blip) for a large fraction of participants and that it is thus clinically relevant to follow up these participants.

8.4. Resistance survey at months 0 and 24

8.4.1. Background

In Lesotho, as in large parts of SSA, Dolutegravir (DTG) is being rolled out as the first-line treatment choice and preferred second-line drug for those failing non-nucleoside reverse transcriptase inhibitors with no history of raltegravir use by Mid/End 2019. However, first case report(s) of DTG resistance have been published (44) and information on DTG resistance and treatment outcomes in the context of non-B subtypes, and infrequent VL monitoring is scarce (45). Thus, there is a need for longitudinal clinical surveillance and resistance surveys during the large-scale roll-out of DTG.

8.4.2. Methods

The VITAL trial harbours ideal prerequisites to perform such longitudinal surveillance in a real-life setting. The first blood samples collected for the VITAL trial are approximately within 6 months after the roll-out of DTG in Lesotho, while the last blood samples collected will be 2 years thereafter. With 18 clinics participating from Lesotho with a mean number of 346 participants and assuming a failure rate of 10%, we expect over 600 participants with unsuppressed VL per time point.

8.5. The impact of an aDSDM on cervical cancer screening coverage

8.5.1. Background

Cervical cancer is the leading cause of cancer deaths for women in most sub-Saharan African countries, including Lesotho (46). Women living with HIV are four to five times more likely to develop invasive cervical cancer (47). Because cervical cancer is a preventable disease through early detection, this nested study aimes at quantifying the cervical cancer screening coverage and understanding the barriers to cervical cancer screening among women living with HIV at health centers enrolled in the VITAL trial. Further, this nested study will test if the VITAL eHealth support may result in increased cervical cancer screening coverage in intervention clinics.

8.5.2. Methods

At enrollment all female participants will be asked whether and when they had last attended a cervical cancer screening. Data regarding cervical cancer screening will be collected in control and intervention clusters (*Appendix 15.2*). In intervention clusters, the recommended action regarding

cervical cancer screening will be displayed as per National guidelines in Lesotho on the VITAL app of the nurse. In addition, in intervention clusters all female participants who agreed to receive health related SMS at enrollment, will receive informative text messages on cervical cancer (screening) and all participants due for screening will receive reminder text messages.

8.5.1. Hypothesis

Low awareness and socioeconomic barriers may lead to underutilization of cervical cancer services among women living with HIV in Lesotho. Thus, we hypothesize that eHealth tools providing information and reminders on cervical cancer screening may empower women to take ownership of their health and to attend the recommended annual screening. Recommendations on cervical cancer screening on the nurse's app may further increase screening rates in intervention clusters.

8.6. Qualitative nested studies

8.6.1 Objective: The goal of this sub-study is to conduct an in-depth exploration of and receive feedback on (1) aspects of the VITAL intervention to inform necessary adaptations and (2) mental health needs in the community and possible intervention strategies to inform the development of a mental health intervention to be delivered within VITAL.

8.6.2. Rationale: The VITAL intervention has now been instituted in all participating clinics in the study with over 2300 participants enrolled in total (anticipating a sample size of over 2655 per arm). There are several observations based on data collected to date that would help us better understand participant behavior in the study and potentially make adaptations to current and future interventions.

First, the VITAL intervention uses SMSs to support provision of differentiated patient healthcare. Participants provide their preferences for the frequency with which they wish to receive messages (for ART intake reminders) and the type of messages they would like to receive (ART intake reminders, clinic visit reminders, VL notification). However, we do not know the extent to which participants read the messages sent to them, whether the messages are helpful in participants' uptake of health services, and whether participants have recommendations for changes to the message content.

Second, we have observed in the data so far that over one-third (~36%) of women in the study report never having tested for cervical cancer. It is unclear why this number is so high, as per guidelines in Lesotho all HIV-infected women should be screened for cervical cancer upon HIV diagnosis and annually thereafter (visual inspection with acetic acid is recommended for premenopausal females and Pap smears for postmenopausal women). Cervical cancer is the most common cancer among Basotho women and causes the most cancer-related deaths in this group. Women living with HIV are four to five times more likely to develop invasive cervical cancer.
The VITAL intervention is designed to be a differentiated healthcare platform that increases uptake of a variety of healthcare services, including cervical cancer, by supporting nurses and participants by eHealth tools. Thus, it is important to understand what barriers may exist to women's uptake of cervical cancer services, in order to design a suitable intervention.

Third, the VITAL study also assesses common mental disorders in participants, including depression, anxiety, substance use, and trauma. The data collected from VITAL so far suggests a sizeable mental health burden. For example, about 20% of the sample reports at least mild depressive symptoms and 12% of the sample has problematic alcohol use. Unfortunately, the availability of evidence-based mental health treatment in primary care clinics in Butha-Buthe and Mokhotlong is scarce. We therefore wish to develop a culturally-sensitive intervention to address the mental health burden that can feasibly be delivered by non-specialist providers.

8.6.3. Study aims: The aims of the qualitative sub-study is to understand in greater depth the following domains:
(1) patient preferences and feedback on the SMSs received in the VITAL intervention

(2) barriers and facilitators to uptake of a variety of health services, including women's cervical cancer screening and automated TB screening calls
(3) local conceptualizations of depression and substance use and feedback on potential intervention components and delivery methods

8.6.4. Study design and procedures: We will conduct in-depth individual interviews (IDIs) with a subset of participants who are enrolled in the VITAL study. There will be two sub-studies: one focused on SMS preferences and barriers and facilitators to uptake of health services (including cervical cancer) and one focused on mental health. We will develop semi-structured interview guides to elicit participant responses to the topics.

Participants who are enrolled in VITAL who return to the clinic for a visit and who meet the eligibility criteria (see Table 3) will be invited to participate in the study. Study staff will briefly explain the study and if participants are interested, participants will be scheduled for a time to conduct informed consent and carry out the interview. The informed consent and interview may take place immediately after screening, or at a time more convenient for participants. A trained staff member will conduct informed consent with the participants at a private space at the clinic (see Appendices 3 -- 6 for English and Sesotho informed consent forms for the two qualitative sub-studies). Participants will be given a signed copy of the consent form. Once consented, the research assistant will conduct the interview using a semi-structured interview guide (see Appendices 7-8). All interviews will be audio-recorded. After the interview is complete, the audio-recordings will be stored on Switchdrive platform and will be deleted from the audio device.

Participants are not provided with any remuneration for their participation in the study.

**SMS preferences and barriers and facilitators to uptake of health services**	**Mental health conceptualization and treatment**
Enrolled as a study participant in one of the VITAL intervention clinics	Enrolled as a study participant in VITAL (not necessarily intervention clinic)
Willing to have interview audio-recorded	Scores ≥ 5 on the Patient Health Questionnaire- 9 (PHQ-9) or ≥ 3 (for women)/ ≥ 4 (for men) on the Alcohol Use Disorders Identification Test (AUDIT-C)
	Willing to have interview audio-recorded

Table 3. Eligibility criteria for each sub-study

8.6.5. Sample size: Sample size is guided by the information power model for qualitative research (following Maltrud et al., 2016). See Table 4 for a description of the dimensions and how they relate to the current study. As can be seen from the table, not all elements are knowable in advance. A similar sample size will be needed for both sub-studies, though the mental health sub-study will likely require a slightly larger sample. Based on prior experience conducting qualitative studies, we anticipate enrolling up to 30 participants for each sub-study. However, the exact number will be determined through the data collection process.

Dimensions of information power and description	SMS preferences and barriers and facilitators to uptake of health services	Mental health conceptualization and treatment
Study aim- whether the study aim is narrow or broad. Broad aims require a larger sample size.	Narrow	Broad

DocuSign Envelope ID: 94DBBACD-5D19-4B54-B740-1BBD7E9B6CE6

Sample specificity- whether experiences, knowledge, or characteristics of the sample participants are highly specific to the study aim. Specificity also refers to the variation in relevant experiences and in sample demographics. When participant experiences and characteristics are more focal to the study and/or when there is more variability in participant backgrounds, this is considered "dense" specificity and requires a smaller sample size. Otherwise, specificity is considered "sparse."	Dense	Dense
Established theory- whether the study is guided by existing theory. Use of existing theory requires a smaller sample size.	Use of behavioral economics theory to understand technology preferences and behavior change	Non-theory driven
Quality of interview dialogue- how the interview was conducted and whether the quality of communication allowed for clear and unambiguous dialogues. Clear communication and higher quality dialogues requires a smaller sample size.	Unknowable in advance, though interviewers will be newer to qualitative interviewing	Unknowable in advance, though interviewers will be newer to qualitative interviewing
Analysis strategy- whether the analysis focuses on in-depth narratives/discourses (case analysis) versus cross-case analysis comparing responses of different participants. Case analysis requires a smaller sample size. Exploratory analyses also require a smaller sample size.	Cross-case analysis	Cross-case analysis

Table 4. *Dimensions of the information power model for qualitative sample size calculations*

8.6.6. Data analysis: We plan to conduct thematic analysis for both sub-studies. All interviews will be audio-recorded, translated to English, and transcribed. We may use software to support transcription (https://otter.ai/login). Transcripts will be read by trained members of the research team. Ideas and notes about the content will be made and a list of codes will be generated. The codes will form a codebook, which will include the identified codes, definitions, and example quotes. Transcripts will be coded by two coders. Once a minimum acceptable level of interrater reliability has been achieved, subsequent interviewers will be coded individually. Ongoing discrepancies will be reviewed and discussed as a team. Discrepancies will be resolved via consensus.

9 REGULATORY ASPECTS AND SAFETY

The VITAL study is a pragmatic trial where clinical management of participants remains with the health personnel of participating health centers, under the responsibility of the Ministry of Health, following the National guidelines. The VITAL study team does not itself take any decisions regarding management of participants. The VITAL intervention will provide alerts, reminders and recommendations according to monitoring and management algorithms in national treatment guidelines as outlined in Study procedures. This will facilitate the best possible information flow

DocuSign Envelope ID: 94DBBACD-5D19-4B54-B740-1BBD7E9B6CE6

between laboratory results, clinic staff and participants. The VITAL study team will, however, not interfere with the clinical management of its participants. The clinician in charge may overrule at anytime the recommendations provided by the VITAL app. The responsibility for correct medical management of patients in line with National treatment guidelines in Lesotho will thus entirely remain with the clinic staff in charge of the patient.

This study is conducted in compliance with the protocol, the current version of the Declaration of Helsinki, the ICH-GCP, the HRA as well as other locally relevant legal and regulatory requirements.

9.1. Serious Adverse Events

A Serious Adverse Event (SAE) (ClinO, Art. 63) is any untoward medical occurrence that

- Results in death or is life-threatening,
- Requires in-participant hospitalisation or prolongation of existing hospitalisation,
- Results in persistent or significant disability or incapacity, or
- Causes a congenital anomaly or birth defect

The Sponsor/Chief Investigator will consult with the Principal Investigator and/or Local Principal Investigator as well as with the health care professionals in charge at the clinic where the SAE occured and will make i) an assessment of causality of the intervention, and ii) a severity assessment according to the Division of AIDS Table for Grading the Severity of Adult and Pediatric Adverse Events, Corrected Version 2.1, 2017 (48).

Relationship	Description
Possibly	Causal relationship cannot be ruled out
Not related	Causal relationship can be ruled out

9.1.1. Reporting of SAEs

Prescription and use of ART will follow current national guidelines of Lesotho. All ART used in Lesotho have a well-established safety profile. The most frequent adverse events are summarized on page 54 of the Lesotho national guidelines on the use of antiretroviral therapy for HIV prevention and treatment, 5th edition, 2016 (49) and on page 138 of the consolidated guidelines on the use of antiretroviral drugs for treating and preventing HIV infection of the WHO (1). SAE will be graded according to the Division of AIDS Table for Grading the Severity of Adult and Pediatric Adverse Events, Corrected Version 2.1, 2017 (48) and managed according to study sites standard procedure following the national guidelines. The VITAL study team will advise in management of SAEs where appropriate. Responsibility for correct management and up-referral if indicated is with the treating health care professionals at the clinics. In case of SAEs, health care personnel at VITAL clinics is instructed to inform the VITAL study team using a dedicated phone number within 72 hours of his/her awareness of the SAE. First person of contact is usually the DAN, otherwise the local principal investigator. DAN/local principal investigator must then inform the Sponsor/Chief investigator within 72 hours of their awareness of the SAE. If a causal relationship cannot be ruled out, the Sponsor/Chief investigator will inform the local ethics committee in Lesotho within 72 hours of his awareness. The health care personnel at clinics is responsible for all direct safety procedures among study participants.

9.1.2. Follow up of SAEs

Because VITAL study is a pragmatic trial where participants are managed by the health care personnel at the health centers the VITAL study team has to rely on the health center's personnel to alert in case of a potential SAE. To improve reporting of SAEs, the VITAL study app will have a feature that serves as a reminder for the health care provider to report in case of suspected SAE. At every clinic visit the nurse is asked by the VITAL app if the participant is or was seriously ill (life-threatening)

and a positive answer triggers a message to the District ART Nurse (DAN) ans subsequently further assessment and reporting of SAE will follow the procedure as outlined in section 9.1.1.

9.2. Periodic safety reporting

SAEs possibly related to the VITAL intervention will be reported to the Ethics Committee of Lesotho by the sponsor/chief investigator within 72 hours he became aware of the SAE in a detailed SAE report form. The annual report to the Ethics Committee in Lesotho on the VITAL study progress will contain a line listing of all SAEs (independent of causal relationship to VITAL).

9.3. Amendments

Substantial changes to the study setup and study organisation, the protocol and relevant study documents are submitted to the Ethics Committee in Lesotho for approval before implementation. Under emergency circumstances, deviations from the protocol to protect the rights, safety and well-being of human subjects may proceed without prior approval of the Ethics Committee. Such deviations shall be documented and reported to the Ethics Committee in Lesotho as soon as possible. A list of all non-substantial amendments will be submitted once a year as part of the annual study progress report to the Ethics Committee in Lesotho.

9.4. (Premature) termination of study

No interim analysis will be performed for the VITAL trial. However, the Sponsor/Chief-Investigator may terminate the study prematurely according to certain circumstances, e.g.

- Ethical concerns,
- Insufficient participant recruitment,
- When the safety of the participants is doubtful or at risk (e.g. when the benefit-risk assessment is no longer positive),
- Alterations in accepted clinical practice that make the continuation of the study unwise, or
- Early evidence of harm of the VITAL intervention will be evaluated bi-monthly: Technical data on information flow and clinical data which is not part of any of the primary or secondary endpoints will be analyzed.

Upon regular study termination, the Ethics Committee is notified via BASEC within 90 days (ClinO, Art. 38).

Upon premature study termination or study interruption, the Ethics Committee is notified via BASEC within 15 days (ClinO, Art. 38).

9.5. Insurance

In the event of study-related damage or injuries, the liability of the insurance of the University Hospital Basel provides compensation, except for claims that arise from participants' misconduct or gross negligence.

10 FURTHER ASPECTS

10.1. Risk-benefit assessment

The physical risk of the VITAL trial to participants is minimal and is limited to risks related to decreased clinic visit frequencies of virally suppressed participants. However, there is little evidence that participants seen more frequently have better clinical outcomes (14). Participants will not miss any laboratory tests due to the participation in this trial and the VL monitoring schedule is strictly followed. If participants stay away from the clinic >3 months, they will receive an automated and interactive TB

DocuSign Envelope ID: 94DBBACD-5D19-4B54-B740-1BBD7E9B6CE6

screening call after 3 months. The study harbours a minimal social risk of involuntary disclosure of HIV status due to voluntary and encrypted text-messages (ART intake reminders).
The VITAL trial has the potential to improve HIV care and harbours health benefits for participants. Text-messages will potentially bring participants with high VL results back to care faster and once in care, and health care workers will have more time to focus on these participants. For EAC, participants may choose an option that suits their needs the best; giving them ownership of their therapy and their health and excluding intensive clinic visit schedules as a reason for defaulting care (7). If a participant has a 2nd VL above 1'000c/ml or 3rd VL above 20c/ml, a second-line application is generated automatically from VL database and appears as an alert to the nurse for confirmation/ submission to second-line committee. Thus, this trial has the potential of speeding up switches to second-line, by (i) bringing participants back to care faster after high VLs, (ii) reducing the workload associated with second-line requests for nurses and (iii) not awaiting the participants' next visit before preparing the second-line request if indicated (i.e. ideally, the participant could be switched at the subsequent visit already). Decreasing the time to switch could potentially reduce morbidity, mortality, risk of (additional) drug resistance development and onward transmission of the (potentially resistant) virus (16–20).
Importantly, the VITAL trial tests a potentially cost-effective monitoring strategy for people taking ART, which is crucial for the sustainability of VL monitoring in SSA (4). Thus, this trial has a high social and scientific value.

10.2. Overall ethical considerations

Establishing cost-effective monitoring strategies for people taking ART is crucial for the sustainability of ART programs in SSA (4), and VL-driven differentiated service delivery models have been proposed to achieve this goal (5). The VITAL trial tests the cost-effective and non-inferiority to the standard of care of an aDSDM and at the same time aims at improving HIV care in Lesotho.
The risk of the VITAL trial to participants is minimal. Participation is entirely voluntary, and informed consent can be withdrawn at any time.
All data obtained in this study will belong to the International Clinical and Health Services Research Unit, Clinical Epidemiology, Department of Clinical Research, University Hospital Basel, University of Basel. The Sponsor-Investigator may make the data and samples available to VITAL study team members or external researchers for GCP-conform further use.
The evidence generated in the VITAL trial is intended to inform future national and international clinical guidelines. The study intervention was intentionally designed in a way such that in case of a non-inferior outcome, it will be feasible to upscale it to a national level. Furthermore, evidence generated from nested studies (e.g. on the change of local drug resistance profiles) may inform local/national policies.

11 QUALITY CONTROL AND DATA PROTECTION

11.1. Quality measures

All personnel working on the VITAL trial will attend training workshops before the start of the trial. Adherence to study protocol of intervention clusters will be evaluated bi-monthly and inform quality visits of study coordinator at each study clinic (both intervention and control clusters), also planned on a bi-monthly basis. Poor adherence to protocol will trigger further study-specific training.

Additional monitoring activities will be carried out through a trial monitor from or trained by the Clinical Operations Unit of the Swiss Tropical and Public Health Institute (Swiss TPH).

The sponsor / chief investigator or the Ethics Committee may visit the research sites for quality assurance. Direct access to the source data and all study-related files is granted on such occasions. All involved parties keep the participant data strictly confidential.

11.2. Data recording and source data

DocuSign Envelope ID: 94DBBACD-5D19-4B54-B740-1BBD7E9B6CE6

The data management is an essential part of the VITAL trial and is in detail described in 4.3. All study data will be uploaded to the VL database through study tablets or computers. The VL database has been approved (Study entitled "*Implementation of routine viral load monitoring in rural Lesotho: A prospective cohort-study on virologic outcomes among patients on antiretroviral therapy in Lesotho*" (ID-134-2016)). On the VL database, participants will be coded using their ART number and VL database specific identifiers, no participant names will be shown to the investigators or health care worker involved in the trial.

All data for the study is entered to the VITAL app and stored on the VL database, which acts as the primary source document. If data is missing on the VL database, routinely collected medical records at the health centers, primarily the patient file, if necessary also participants' health booklet (bukana), antenatal care register, postnatal care register, ART register, ART treatment card and file, TB treatment register, TB treatment card, pharmacy register/dispensing logs and the cervical cancer screening register may additionally act as source documents.

11.3. Confidentiality and coding

Trial and participant data will be handled with uttermost discretion and is only accessible to authorised personnel who require the data to fulfil their duties within the scope of the study. The data management of this study will be performed using an extension of the already existent VL database (Study entitled "*Implementation of routine viral load monitoring in rural Lesotho: A prospective cohort-study on virologic outcomes among patients on antiretroviral therapy in Lesotho*" (ID-134-2016)). The VL database incorporates the following safety measures: (i) two factor authentification, and (ii) Participants' names are encrypted. The data are stored in banking-standard ISO 27001-audited data centres in Switzerland. Nurses and VRAs at health centres will identify participants by searching their ART number using the VITAL application on the study tablet.

Biological material in this study is not identified by participant name but by a unique participant number (ART number). Biological material is appropriately stored in a restricted area only accessible to the authorised personnel.

11.4. Retention and destruction of study data and biological material

All study data will be archived for 25 years after study termination or premature termination of the study.
The collection of biological material will follow usual procedures at the respective health centres and that blood leftover after VL testing will routinely be stored according to the Study entitled "*Implementation of routine viral load monitoring in rural Lesotho: A prospective cohort-study on virologic outcomes among patients on antiretroviral therapy in Lesotho*" (ID-134-2016).
All blood samples will be labelled with a unique identifier and stored at the Butha-Buthe Government Hospital laboratory, Butha-Buthe, Lesotho, or ii) Haus Petersplatz, Department of Biomedicine, University of Basel, Basel, Switzerland. Leftover biological material may be used for further biomedical research beyond the scope of the present study, securing the anonymity of participants, according to the above mentioned protocol (ID-134-2016).

12 MONITORING AND REGISTRATION

The trial will undergo monitoring to ensure compliance with the study protocol and good clinical practice (ICH GCP E6R2) principles. For monitoring, the study team will collaborate with the Clinical Operations Unit of the Swiss Tropical and Public Health Institute (Swiss TPH). Local monitors will be trained and perform monitoring visits at health centres. Monitors will have access to source data as well as all study documentation, and the sponsor-investigator, trial coordinator, and/or another central member of the study team will assist them in all their queries. The study has a separate monitoring plan.

DocuSign Envelope ID: 94DBBACD-5D19-4B54-B740-1BBD7E9B6CE6

The trial is registered under clinicaltrials.gov (NCT04527874).

36

13 FUNDING / PUBLICATION / DECLARATION OF INTEREST

This study will be funded through the Swiss National Science Foundation (grant number PCEFP3_181355; obtained by NDL) and the Moritz Straus foundation based in Basel, Switzerland. The funding sources are/will not be not involved in the study design, data collection, data analysis, interpretation of the results, or writing the manuscript. The study will be embedded in the SolidarMed Lesotho country programme and will thus benefit from logistics and human resources of this organisation. The listed co-investigators have no conflicts of interest.

All results of this trial and its nested studies will be published in peer-reviewed journals and coded data will be made available in a public data repository upon publication.

14 REFERENCES

1. World Health Organization. Consolidated guidelines on the use of antiretroviral drugs for treating and preventing HIV infection : recommendations for a public health approach. 2016. 429 p.
2. Grimsrud A, Bygrave H, Doherty M, Ehrenkranz P, Ellman T, Ferris R, et al. Reimagining HIV service delivery: the role of differentiated care from prevention to suppression. Journal of the International AIDS Society [Internet]. 2016 Jan 1;19(1):21484. Available from: https://doi.org/10.7448/IAS.19.1.21484
3. International AIDS Society (IAS). Differentiated care for HIV: a decision framework for antiretroviral therapy delivery. Durban, South Africa. 2016;
4. Phillips A, Shroufi A, Vojnov L, Cohn J, Roberts T, Ellman T, et al. Sustainable HIV treatment in Africa through viral-load-informed differentiated care. Nature. 2015 Dec;528(7580):S68-76.
5. Barnabas R V, Revill P, Tan N, Phillips A. Cost-effectiveness of routine viral load monitoring in low- and middle-income countries: a systematic review. Journal of the International AIDS Society. 2017 Nov;20 Suppl 7.
6. Ehrenkranz PD, Baptiste SL, Bygrave H, Ellman T, Doi N, Grimsrud A, et al. The missed potential of CD4 and viral load testing to improve clinical outcomes for people living with HIV in lower-resource settings. PLoS medicine. 2019 May;16(5):e1002820.
7. Ware NC, Wyatt MA, Geng EH, Kaaya SF, Agbaji OO, Muyindike WR, et al. Toward an understanding of disengagement from HIV treatment and care in sub-Saharan Africa: a qualitative study. PLoS medicine. 2013;10(1):e1001369; discussion e1001369.
8. Glass TR, Motaboli L, Nsakala B, Lerotholi M, Vanobberghen F, Amstutz A, et al. The viral load monitoring cascade in a resource-limited setting: A prospective multicentre cohort study after introduction of routine viral load monitoring in rural Lesotho. PLOS ONE [Internet]. 2019 Aug 28;14(8):e0220337. Available from: https://doi.org/10.1371/journal.pone.0220337
9. Labhardt ND, Ringera I, Lejone TI, Cheleboi M, Wagner S, Muhairwe J, et al. When patients fail UNAIDS' last 90 - the "failure cascade" beyond 90-90-90 in rural Lesotho, Southern Africa: a prospective cohort study. Journal of the International AIDS Society. 2017 Jul;20(1):21803.
10. UNAIDS. UNAIDS data 2019 [Internet]. 2019 [cited 2019 Sep 2]. Available from: https://www.unaids.org/sites/default/files/media_asset/2019-UNAIDS-data_en.pdf
11. Nash D, Yotebieng M, Sohn AH. Treating all people living with HIV in sub-Saharan Africa: a new era calling for new approaches. Journal of virus eradication [Internet]. 2018 Nov 15;4(Suppl 2):1–4. Available from: https://www.ncbi.nlm.nih.gov/pubmed/30515307
12. World Health Organization. Consolidated guidelines on the use of antiretroviral drugs for treating and preventing HIV infection : recommendations for a public health approach. [Internet]. 2013 [cited 2018 Apr 30]. 269 p. Available from: https://www.ncbi.nlm.nih.gov/books/NBK195400/pdf/Bookshelf_NBK195400.pdf
13. WHO. HIV DRUG RESISTANCE REPORT. 2017 [cited 2018 Apr 24]; Available from: http://apps.who.int/iris/bitstream/handle/10665/255896/9789241512831-eng.pdf;jsessionid=ED8F5ED51C7C2D65906A7230871ED51A?sequence=1
14. Tagar E, Sundaram M, Condliffe K, Matatiyo B, Chimbwandira F, Chilima B, et al. Multi-Country Analysis of Treatment Costs for HIV/AIDS (MATCH): Facility-Level ART Unit Cost Analysis in Ethiopia, Malawi, Rwanda, South Africa and Zambia. PLOS ONE [Internet]. 2014 Nov 12;9(11):e108304. Available from: https://doi.org/10.1371/journal.pone.0108304
15. Mody A, Roy M, Sikombe K, Savory T, Holmes C, Bolton-Moore C, et al. Improved Retention With 6-Month Clinic Return Intervals for Stable Human Immunodeficiency Virus-Infected Patients in Zambia. Clinical infectious diseases : an official publication of the Infectious Diseases Society of America [Internet]. 2018 Jan 6;66(2):237–43. Available from: https://www.ncbi.nlm.nih.gov/pubmed/29020295

DocuSign Envelope ID: 94DBBACD-5D19-4B54-B740-1BBD7E9B6CE6

16. Narainsamy D, Mahomed S. Delays in switching patients onto second-line antiretroviral treatment at a public hospital in eThekwini, KwaZulu-Natal. Southern African Journal of HIV Medicine; Vol 18, No 1 (2017) [Internet]. 2017; Available from: https://sajhivmed.org.za/index.php/hivmed/article/view/696
17. Shroufi A, Van Cutsem G, Cambiano V, Bansi-Matharu L, Duncan K, Murphy RA, et al. Simplifying switch to second-line ART: Predicted effect of defining failure of first-line efavirenz-based regimens in sub-Saharan Africa by a single viral load more than 1000 copies/ml. AIDS (London, England). 2019 Jun;
18. Ramadhani HO, Bartlett JA, Thielman NM, Pence BW, Kimani SM, Maro VP, et al. The Effect of Switching to Second-Line Antiretroviral Therapy on the Risk of Opportunistic Infections Among Patients Infected With Human Immunodeficiency Virus in Northern Tanzania. Open forum infectious diseases [Internet]. 2016 Jan 29;3(1):ofw018–ofw018. Available from: https://www.ncbi.nlm.nih.gov/pubmed/26949717
19. Petersen ML, Tran L, Geng EH, Reynolds SJ, Kambugu A, Wood R, et al. Delayed switch of antiretroviral therapy after virologic failure associated with elevated mortality among HIV-infected adults in Africa. AIDS (London, England). 2014 Sep;28(14):2097–107.
20. Murphy RA, Court R, Maartens G, Sunpath H. Second-Line Antiretroviral Therapy in Sub-Saharan Africa: It Is Time to Mind the Gaps. AIDS research and human retroviruses. 2017 Dec;33(12):1181–4.
21. Ssempijja V, Nakigozi G, Chang L, Gray R, Wawer M, Ndyanabo A, et al. Rates of switching to second-line antiretroviral therapy and impact of delayed switching on immunologic, virologic, and mortality outcomes among HIV-infected adults with virologic failure in Rakai, Uganda. BMC infectious diseases. 2017 Aug;17(1):582.
22. El-Sadr WM, Rabkin M, Nkengasong J, Birx DL. Realizing the potential of routine viral load testing in sub-Saharan Africa. Journal of the International AIDS Society [Internet]. 2017 Nov 12;20 Suppl 7(Suppl Suppl 7):e25010. Available from: https://www.ncbi.nlm.nih.gov/pubmed/29130621
23. Carmona S, Peter T, Berrie L. HIV viral load scale-up: multiple interventions to meet the HIV treatment cascade. Current opinion in HIV and AIDS. 2017 Mar;12(2):157–64.
24. Schwartz SR, Kavanagh MM, Sugarman J, Solomon SS, Njindam IM, Rebe K, et al. HIV viral load monitoring among key populations in low- and middle-income countries: challenges and opportunities. Journal of the International AIDS Society. 2017 Nov;20 Suppl 7.
25. Marcus R, Ferrand RA, Kranzer K, Bekker L-G. The case for viral load testing in adolescents in resource-limited settings. Journal of the International AIDS Society. 2017 Nov;20 Suppl 7.
26. Arpadi SM, Shiau S, De Gusmao EP, Violari A. Routine viral load monitoring in HIV-infected infants and children in low- and middle-income countries: challenges and opportunities. Journal of the International AIDS Society [Internet]. 2017 Nov 24;20 Suppl 7(Suppl Suppl 7):e25001. Available from: https://www.ncbi.nlm.nih.gov/pubmed/29171190
27. UNAIDS. 90–90–90 - An ambitious treatment target to help end the AIDS epidemic | UNAIDS [Internet]. 2014 [cited 2019 Aug 1]. Available from: https://www.unaids.org/en/resources/documents/2017/90-90-90
28. Wilkinson T, Sculpher MJ, Claxton K, Revill P, Briggs A, Cairns JA, et al. The International Decision Support Initiative Reference Case for Economic Evaluation: An Aid to Thought. Value in health : the journal of the International Society for Pharmacoeconomics and Outcomes Research. 2016 Dec;19(8):921–8.
29. UNAIDS. Lesotho 2018 [Internet]. [cited 2019 Sep 30]. Available from: https://www.unaids.org/en/regionscountries/countries/lesotho
30. LePHIA report. LESOTHO POPULATION-BASED HIV IMPACT ASSESSMENT. 2016 [cited 2018 Apr 30]; Available from: http://phia.icap.columbia.edu/wp-content/uploads/2018/02/Lesotho-Summary-Sheet_A4.2.7.18.HR_.pdf
31. Moyé LA. Multiple analyses in clinical trials: fundamentals for investigators. Springer; 2003.
32. Lesaffre E. Use and misuse of the p-value. Bulletin of the NYU hospital for joint diseases. 2008;66(2):146–9.
33. European Medicine Agency. Guideline on multiplicity issues in clinical trials. 2016;44(December 2016):1–15.
34. WHO. PACKAGE OF CARE FOR ADVANCED HIV DISEASE POLICY BRIEF HIV TREATMENT [Internet]. 2018 [cited 2019 Sep 13]. Available from: http://www.who.int/medicines/publications/
35. Jaffar S, Amuron B, Foster S, Birungi J, Levin J, Namara G, et al. Rates of virological failure in patients treated in a home-based versus a facility-based HIV-care model in Jinja, southeast Uganda: a cluster-randomised equivalence trial. Lancet (London, England). 2009 Dec;374(9707):2080–9.
36. Geldsetzer P, Francis JM, Ulenga N, Sando D, Lema IA, Mboggo E, et al. The impact of community health worker-led home delivery of antiretroviral therapy on virological suppression: a non-inferiority cluster-randomized health systems trial in Dar es Salaam, Tanzania. BMC Health Services Research [Internet]. 2017;17(1):160. Available from: https://doi.org/10.1186/s12913-017-2032-7
37. Chow S-C, Shao J, Wang H, Lokhnygina Y, Shao J, Wang H, et al. Sample Size Calculations in Clinical Research: Third Edition [Internet]. Chow S-C, Shao J, Wang H, Lokhnygina Y, editors. Third edition. | Boca Raton : Taylor & Francis, 2017. | Series: Chapman & Hall/CRC biostatistics series | “A CRC title, part of the Taylor & Francis imprint, a member of the Taylor & Francis Group, the academic division of T&F Informa plc.”: Chapman and Hall/CRC; 2017 [cited 2019 Oct 3]. Available from: https://www.taylorfrancis.com/books/9781351727129
38. Rotondi M, Donner A. Sample size estimation in cluster randomized trials: An evidence-based perspective. Computational Statistics & Data Analysis [Internet]. 2012;56(5):1174–87. Available from: http://www.sciencedirect.com/science/article/pii/S0167947310004780
39. Wang H, Chow S-C, Li G. ON SAMPLE SIZE CALCULATION BASED ON ODDS RATIO IN CLINICAL TRIALS. Journal of Biopharmaceutical Statistics [Internet]. 2002 Jan 12;12(4):471–83. Available from: https://doi.org/10.1081/BIP-120016231
40. Douglas Bates, Martin Maechler, Ben Bolker SW. Fitting Linear Mixed-Effects Models Using lme4. Journal of Statistical Software. 2015;67(1):1–48.
41. Eldridge SM, Ashby D, Kerry S. Sample size for cluster randomized trials: effect of coefficient of variation of cluster size and analysis method. International journal of epidemiology. 2006 Oct;35(5):1292–300.

42. Campbell MK, Piaggio G, Elbourne DR, Altman DG. Consort 2010 statement: extension to cluster randomised trials. BMJ : British Medical Journal [Internet]. 2012 Sep 4;345:e5661. Available from: http://www.bmj.com/content/345/bmj.e5661.abstract
43. Amstutz A, Nsakala BL, Vanobberghen F, Muhairwe J, Glass TR, Achieng B, et al. SESOTHO trial ("Switch Either near Suppression Or THOusand") – switch to second-line versus WHO-guided standard of care for unsuppressed patients on first-line ART with viremia below 1000 copies/mL: protocol of a multicenter, parallel-group, open-label, r. BMC Infectious Diseases [Internet]. 2018;18(1):76. Available from: https://doi.org/10.1186/s12879-018-2979-y
44. McKellar MS, Okeke NL, McGee KS, Hurt CB. Canary in the Coal Mine? Transmitted Mutations Conferring Resistance to All Integrase Strand Transfer Inhibitors in a Treatment-Naive Patient. Open Forum Infectious Diseases [Internet]. 2018 Nov 8;5(11). Available from: https://dx.doi.org/10.1093/ofid/ofy294
45. Dorward J, Hamers RL. Dolutegravir in sub-Saharan Africa: context is crucial. The Lancet HIV [Internet]. 2019 Feb 1;6(2):e72–3. Available from: https://doi.org/10.1016/S2352-3018(18)30331-X
46. Collaboration GB of DC. Global, Regional, and National Cancer Incidence, Mortality, Years of Life Lost, Years Lived With Disability, and Disability-Adjusted Life-years for 32 Cancer Groups, 1990 to 2015: A Systematic Analysis for the Global Burden of Disease Study. JAMA Oncology [Internet]. 2017 Apr 1;3(4):524–48. Available from: https://doi.org/10.1001/jamaoncol.2016.5688
47. UNAIDS. Cervical cancer and HIV—two diseases, one response [Internet]. 2018 [cited 2019 Nov 6]. Available from: https://www.unaids.org/en/resources/presscentre/featurestories/2018/october/cervical-cancer-and-hiv
48. Division of AIDS. Division of AIDS (DAIDS) Table for Grading the Severity of Adult and Pediatric Adverse Events [Internet]. 2017 [cited 2019 Oct 7]. Available from: https://rsc.niaid.nih.gov/sites/default/files/daidsgradingcorrectedv21.pdf
49. Ministry of Health, Government of Lesotho. NATIONAL GUIDELINES ON THE USE OF ANTIRETROVIRAL THERAPY FOR HIV PREVENTION AND TREATMENT Fifth Edition [Internet]. 2016 [cited 2019 Oct 7]. Available from: https://aidsfree.usaid.gov/sites/default/files/lesotho_art_2016.pdf

DocuSign Envelope ID: 94DBBACD-5D19-4B54-B740-1BBD7E9B6CE6

15 APPENDICES

15.1. Case report form 1: Variables collected at enrolment

Variables on gray background are only collected in the intervention clinics, all others are collected independent of study arm allocation. Variables on pink background are only collected for female participants.

Variable	*Categories*	*Format*
**General information**		
Clinic	*1. Makhunoane<br>2. Linakeng<br>3. Tsime<br>4. St. Peters<br>5. St. Paul<br>6. Boiketsiso<br>7. Motete<br>8. Rampai<br>9. Ngoajane<br>10. Muela<br>11. Malefiloane<br>12. St. James<br>13. Moeketsane<br>14. Mapholaneng<br>15. Linakaneng<br>16. Molikaliko<br>17. Libibing<br>18. St Martins*	*Categorical*
ART number		*L/MM/NNNNN<br>L/MM/NNNNNN<br>L/MM/NNNNN/$N<br>L/MM/NNNNNN/$N<br><br>Where<br>L is a letter (A-Z),<br>M is a number (0-9) or a letter (only A,I,N),<br>N is a number (0-9).*
Participant name		*Not collected by the VRA, but displayed by the VITALapp if participant has entries on the VL database*
Participant initals		*Not collected by the VRA, but displayed by the VITALapp if participant has entries on the VL database*
Sex		*Binary<br>Prefilled in VITALapp*
Birthdate		*Predefined date format<br>Prefilled in VITALapp*
Consent for VITAL given	▪ *Yes*<br>▪ *No*	*If “No” no data can be collected by the VITALapp*
Consent for VL monitoring cohort	▪ *Yes*<br>▪ *No*	*If “No” no VL samples*

study given		*will be biobanked*
Date of VITAL enrolment		*date format*
Date of VITAL consent		*date format*
Completed educational level? (at enrolment)	▪ *None*<br>▪ *Primary*<br>▪ *Secondary*<br>▪ *High-school*<br>▪ *Tertiary*	*Categorical*
Employment situation? (at enrolment)	▪ *Employed in Lesotho*<br>▪ *Employed in RSA*<br>▪ *Self-employed with regular income*<br>▪ *Subsistence farming*<br>▪ *Housewife*<br>▪ *No regular income / unemployed*	*Categorical*
Marital status?	▪ *single, never married*<br>▪ *married*<br>▪ *domestic partnership*<br>▪ *divorced*<br>▪ *separated*<br>▪ *widowed*<br>▪ *prefers not to answer*	
travel time to clinic (one way, in minutes, walking)		*Numeric*
travel cost to clinic by public transport (round trip, maloti)		*Numeric*
village		*Free text*
Do you have your national ID with you?	▪ *Yes*<br>▪ *No*	
If No: Why don't you have it?	▪ *Never made it*<br>▪ *Lost it*<br>▪ *Don't have it with me today*	*Singlechoice*
If Yes: What is your national ID number?		*Prespecified National ID number formal*
**Health-related information**		
Current intake of co-medication?	▪ *Yes*<br>▪ *No*	
Current intake of co-medication?	▪ *Co-trimoxazole (CTX)*<br>▪ *TB treatment*<br>▪ *TB prevention: IPT*<br>▪ *TB prevention: INH+Rifapentine*<br>▪ *TB prevention: other*<br>○ *speecify*<br>▪ *traditional medicine*<br>○ *specify*<br>▪ *high blood pressure medicine*<br>▪ *blood sugar medicine*<br>▪ *Vitamine B6*<br>▪ *other*<br>○ *specify*	*Categorical + free text (other)*
How reliably do you use contraceptives?	▪ *Always*<br>▪ *Usually*<br>▪ *Occasionally*	*Categorical*

	▪ *Rarely*<br>▪ *Never*<br>▪ *Not sexually active*	
If female: *What type(s) of contraceptive(s) / family planning do you use?*	▪ *Oral*<br>▪ *Injectables*<br>▪ *IUD (Intrauterine device)*<br>▪ *Implants*<br>▪ *Condom/Barrier*<br>▪ *Other*	*Categorical + free text*
If female: *Have you ever been screened for cervical cancer?*	▪ *No*<br>▪ *Yes, within the last year*<br>▪ *Yes, 1-2 years ago*<br>▪ *Yes, more than 2 years ago*	
**Adherence information**		
History of treatment interruption > 1 week?	*If yes: duration (in weeks)*	*Binary + Numeric*
Did you miss ART at two or more consecutive days within the last month?	▪ *Yes*<br>▪ *No*	*Binary*
pillcount available within the last 6 months?	▪ *Yes*<br>▪ *No*	*Binary*
adherence according to pill count (% from pill count, if on a two pill regimen separate pillcounts by comma)	*(% from pill count)*	*If pillcount available within the last 6 months*
**VL database update**		
Antiretroviral drugs of current ART regimen	▪ *D4T*<br>▪ *AZT*<br>▪ *ABC*<br>▪ *TDF*<br>▪ *3TC*<br>▪ *EFV*<br>▪ *NVP*<br>▪ *LPV/r*<br>▪ *ATV*<br>▪ *DTV*<br>▪ *DTG*<br>▪ *RAL*<br>▪ *other*<br>*Specify (text)*	*Categorical + free text*<br>*Prefill (if available)*
Treatment line	*1,2,3*	
Start date of current ART regimen		*Predefined date format*<br>*Prefill*
Date of ART initiation		*Predefined date format*<br>*Prefill*
Date of HIV diagnosis		*Predefined date format*
Date of first CD4 cell count		*Predefined date format*<br>*Prefill*
First CD4 cell count		*Prefill*
previous ART	▪ *AZT*<br>▪ *TDF*<br>▪ *3TC*<br>▪ *EFV*<br>▪ *NVP*	*Categorical, choose more than one or none*

	▪ *LPV/r*<br>▪ *ATV*<br>▪ *DRV*<br>▪ *DTG*<br>▪ *RAL*	
Previous ART start and stop date		*Date format*
Date of HIV diagnosis		*date format*
**eHealth information**		
Do you or your family have access to a phone where we can send confidential information on?	*If yes:*<br>▪ *First mobile owner*<br>▪ *First phone number*<br>▪ *Second mobile owner*<br>▪ *Second phone number*	*Binary + phone number format (LS or RSA format)*
Is there someone we can call if we do not reach you? (i.e. village health worker)	*If yes:*<br>*Phone number*	*Binary + phone number format*<br>*(LS or RSA format)*
Can we send you encoded SMS on your laboratory results? *(show examples)*	***SMS examples***<br>▪ ***VL < 50***<br>*Congratulations on good test ([result])! Keep it up! Discuss long-term supply! Greetings, [clinic name]*<br>▪ ***VL ≥ 50***<br>*Your test ([result]) is out, please come and see us as soon as possible to discuss support options. Greetings, [clinic name]*<br>▪ ***Technical failure of VL measurement***<br>*Your test should be repeated, please come back as soon as possible, sorry for inconvenience. Greetings, [clinic name]*	*Binary*
Can we send you ART refill visit reminders?		*Binary*
Can we send you general health-related educational SMS?		*Binary*
What is the best day for you to receive SMS or calls?		*Mon-Sun*
What is the best time for you to receive SMS or calls?		*0-24*
Do you wish to receive ART intake reminders independent of your VL result?	*If yes:*<br>▪ *Specify weekday*<br>▪ *Specify time*<br>▪ *Choose message:*<br>o *Meds time (emoji for clock)*<br>o *Nako ea lithlare (emoji for clock)*<br>o *Recharge!*	*Binary, free text and categorical*

	○ *Healthy living!*<br>○ *Bophelo bo botle!*<br>○ *Me and good health!*<br>○ *Nna le bophelo bo botle!*<br>○ *Right time!*<br>○ *Nake e nepahetseng*	
For how many months, would you prefer to take ART supply home?	*1-12*	*categorical*
Can we do your TB screening by automated phone calls?		*Binary*

15.2. Case report form 2: Data downloaded and updated by nurse

Variables on gray background are only collected in the intervention clinics, all others are collected independent of study arm allocation.

**Variable**	***Categories***	***Format***
**List downloaded by nurse**		
Visit date		
ART number		
last VL		*continuous*
last VL date		*predefined date format*
2nd last VL		*Continuous*
2nd last VL date		*predefined date format*
3rd last VL		*Continuous*
3rd last VL date		*predefined date format*
Last TB Xpert Result	*If available*<br>▪ *TB positive, no resistance*<br>▪ *TB positive, Rifampicin resistant*<br>▪ *TB positive, isoniazid resistance*<br>▪ *TB positive, Rifampicin and iso resistance*<br>▪ *TB negative*	*will be imported from LIS to VL database*
Last TB Xpert Date	*If available*	*Predefined date format*
Result from distant TB screening	*If positive: List of symptoms (cough, fevers, night sweats, weight loss)*	*Binary and categorical*
Tuberculosis Preventive Therapy (TPT)	▪ *Currently on TPT*<br>▪ *Never had TPT*<br>▪ *Previously had TPT*	*Categorical*
Contact with DAN	*If yes: "Had contact with DAN for: [free text entered by district art nurse]"*	*Binary and free text*
Recommended due date of next VL		*predefined date format*
**Recommended action HIV**	▪ *No special action / consider long-term supply to [date of next VL]*<br>▪ *Informe about low-level viremia – repeat VL in 3 month [date of next VL]*<br>▪ *Preference based EAC and repeat VL in 3 month [date of next VL]*<br>▪ *ART advisory committee notification*<br>▪ *Repeat VL today*	

**Recommended action TPT**	▪ *Participant on TPT, screening positive, stopped TPT, provided sputum bottles.*<br>▪ *Participant on TPT, screening negative and course of TPT not finished, continued TPT.*<br>▪ *Participant on TPT, screening negative and course of TPT finished, discontinued TPT.*<br>▪ *Participant on TPT, a recent Xpert result was positive, stopped TPT, started TB treatment.*<br>▪ *Participant not on TPT, screening positive, TPT not started, provided sputum bottles.*<br>▪ *Participant not on TPT, screening positive, but a recent Xpert was negative, referred to hospital.*<br>▪ *Screening negative, participant has never had TPT, started TPT.*<br>▪ *Screening negative, and participant has had TPT before, no action taken.*<br>▪ *Screening negative and TPT was interrupted within the last 3 months due to a positive TB screening, but following Xpert result was negative, continued course of TPT.*<br>▪ *Screening negative and TPT was interrupted more than 3 months ago due to a positive TB screening, but following Xpert result was negative, started a new full course of TPT.*<br><br>*Participant not on TPT, a recent Xpert result was positive, started TB treatment.*	
**List filled in by nurse**		
Date of visit		*predefined date format*
Have you been tested for COVID-19 since your last visit (swap)	▪ *Yes / No*	*Binary*
**If female:** Currently pregnant		*Binary*
**If currently pregnant:** expected date of delivery		*predefined date format*
**At first visit after delivery:**<br>pregnancy outcome	▪ live birth<br>▪ still birth<br>▪ any congenital anomaly or birth defect	
**If female:** Currently breastfeeding	▪ *Yes*<br>▪ *No*	*Binary*
Serious illness of participant:<br>Was the participant hospitalized since the last visit or do you have to hospitalize the participant	▪ *Yes*<br>▪ *No*	*Binary + free text*

today?		
Current WHO/ treatment stage	▪ *1/T1*<br>▪ *2/T2*<br>▪ *3/T3*<br>▪ *4/T4*	*Single choice*
Point of care (POC) VL availabe	▪ *Yes*<br>▪ *No*	*Binary*
If Yes:<br>POC VL value	▪	*Free text*
POC VL reporting date	▪ *Calender choice*	*Date format*
Current intake of co-medication?	▪ *Yes*<br>▪ *No*	
Comedication	▪ *Co-trimoxazole (CTX)*<br>▪ *TB treatment*<br>▪ *TB prevention: IPT*<br>▪ *TB prevention: INH+Rifapentine*<br>▪ *TB prevention: other*<br>o *speecify*<br>▪ *traditional medicine*<br>o *specify*<br>▪ *high blood pressure medicine*<br>▪ *blood sugar medicine*<br>▪ *Vitamine B6*<br>▪ *other*<br>▪ *specify*	
ART drugs prescribed	▪ *AZT*<br>▪ *ABC*<br>▪ *TDF*<br>▪ *3TC*<br>▪ *EFV*<br>▪ *NVP*<br>▪ *LPV*<br>▪ *ATV*<br>▪ *DRV*<br>▪ *DTG*<br>▪ *RAL*	*Categorical, choose more than one*
ART drugs start date (and stop date if applicable)		*Date*
TPT given		*Binary*
Requested Xpert for TB	*If yes: enter labnumber*	*Binary + free text*
TB treatment given		*Binary*
Last CD4 cell count		*If available*
Last CD4 cell count date	▪	*If available*<br>*Predefined date format*<br>*Match with database and give warning in case of mismatch*
**HIV action taken**	▪ *No special action taken*<br>▪ *Informed about low-level viremia, repeat VL in 3 months*<br>▪ *Preference based EAC and next VL in 3 months*	*Categorical + predefined date format*

	▪ *ART advisory committee notification done today*<br>▪ *Switched to second-line*<br>▪ *VL done/repeated today*	
**Month ART supply given**		*integer*
**TPT action taken**	▪ *Participant on TPT, screening positive, stopped TPT, provided sputum bottles.*<br>▪ *Participant on TPT, screening negative and course of TPT not finished, continued TPT.*<br>▪ *Participant on TPT, screening negative and course of TPT finished, discontinued TPT.*<br>▪ *Participant on TPT, a recent Xpert result was positive, stopped TPT, started TB treatment.*<br>▪ *Participant not on TPT, screening positive, TPT not started, provided sputum bottles.*<br>▪ *Participant not on TPT, screening positive, but a recent Xpert was negative, referred to hospital.*<br>▪ *Screening negative, participant has never had TPT, started TPT.*<br>▪ *Screening negative, and participant has had TPT before, no action taken.*<br>▪ *Screening negative and TPT was interrupted within the last 3 months due to a positive TB screening, but following Xpert result was negative, continued course of TPT.*<br>▪ *Screening negative and TPT was interrupted more than 3 months ago due to a positive TB screening, but following Xpert result was negative, started a new full course of TPT.*<br>▪ *Participant not on TPT, a recent Xpert result was positive, started TB treatment.*	*Categorical*
If VL ≥ 50: Preference-based EAC	▪ *Standard face-to-face EAC at the clinc*<br>▪ *EAC on phone with DAN*<br>▪ *ART Intake reminders*<br>○ *Preferred weekdays (choice of multiple possible)*<br>○ *preferred time*<br>○ *specify message (free text)*	*Choice of more than one possible*
If VL < 50: ART intake reminders independent of VL	*If yes:*<br>○ *Preferred weekdays (choice of multiple possible)*<br>○ *preferred time*<br>○ *specify message (free text)*	
If < 25 years: Sexually active?	▪ *Yes*<br>▪ *No*	*Binary*
Last cervical cancer screening		*Date*

DocuSign Envelope ID: 94DBBACD-5D19-4B54-B740-1BBD7E9B6CE6

date before today (empty if never screened)?		
Cervical screening done at this visit?	▪ *Yes*<br>▪ *No*	*Binary*
**If screening done:** Cervical screening result	▪ Normal<br>▪ Abnormal	
**If screening done:** Cervical screening method	▪ Visual inspection (VIA)<br>▪ HPV Nucleic acid test<br>▪ Pap smear/cytology	
**Date of next ART refill ***		
Applied for second-line		*Binary*
**Adherence of participants having EAC**		
When was the last time you missed any medications?**	▪ *Past week*<br>▪ *1-2 weeks ago*<br>▪ *3-4 weeks ago*<br>▪ *Never or >4 weeks ago*	*Categorical, choose more than one*
If not "never or >4 weeks ago": What were the reasons for not taking ART?**	▪ *Forgetting*<br>▪ *Busy*<br>▪ *Change to daily routine*<br>▪ *Travelling/ being away from home*<br>▪ *Side effects/ fear of side effects*<br>▪ *Stigma/ fear of being seen taking pills/ nondisclosure*<br>▪ *Run out of pills/ pills lost/ pills stolen*<br>▪ *Stock out at clinic*<br>▪ *Felt well*<br>▪ *Depressed/ alcohol/ daha*<br>▪ *If other: specify (free text)*	*Categorical and text*
How often did you miss ART in the last month?	▪ *Every day*<br>▪ *More than 1/ week*<br>▪ *Once a week*<br>▪ *Once every two weeks*<br>▪ *Once a month*<br>▪ *Never*	*Categorical*
Did you miss ART at two or more consecutive days within the last month?	▪ *Yes*<br>▪ *No*	*Binary*

****Does not appear on Online platform used by district ART nurse.**

Further mental health data is collected by the VRA at enrollment (see enclosed document).

15.3. Case report form 3: Automatically generated ART advisory committee notification

Variable	*Categories*	*Format*
**second-line request**		
Clinic		*Categorical*
Date of report		*Predefined date format*
ART number		
Sex		
Age		

ART start		*predefined date format*
First CD4 result (cells/mL)		*continuous, 0-2500*
Date of first CD4 result		*predefined date format*
last VL		*continuous*
last VL date		*predefined date format*
2nd last VL		*continuous*
2nd last VL date		*predefined date format*
3rd last VL		*continuous*
3rd last VL date		*predefined date format*
Antiretroviral drugs of current ART regimen	▪ *D4T*<br>▪ *AZT*<br>▪ *ABC*<br>▪ *TDF*<br>▪ *3TC*<br>▪ *EFV*<br>▪ *NVP*<br>▪ *LPV/r*<br>▪ *ATV*<br>▪ *DTV*<br>▪ *DTG*<br>▪ *RAL*<br>▪ *other*<br>○ *Specify (text)*	*Categorical + free text*
Antiretroviral drugs previously exposed to	▪ *D4T*<br>▪ *AZT*<br>▪ *ABC*<br>▪ *TDF*<br>▪ *3TC*<br>▪ *EFV*<br>▪ *NVP*<br>▪ *LPV/r*<br>▪ *ATV*<br>▪ *DTV*<br>▪ *DTG*<br>▪ *RAL*	*more than one can be chosen*
Last TB-screening result		*binary*
Last TB-screening date		*Predefined date format*
TB Xpert Result	*If available*	*will be imported from LIS to VL database*
Current intake of co-medication	▪ *Yes / No*	*Binary*
Current intake of co-medication	*If yes*<br>▪ *Co-trimoxazole (CTX)*<br>▪ *TB treatment*<br>▪ *TB prevention: IPT*<br>▪ *TB prevention: INH+Rifapentine*<br>▪ *TB prevention: other*<br>○ *speecify*	*Multiple choice*

DocuSign Envelope ID: 94DBBACD-5D19-4B54-B740-1BBD7E9B6CE6

	▪ *traditional medicine*<br>○ *specify*<br>▪ *high blood pressure medicine*<br>▪ *Blood sugar medicine*<br>▪ *other*<br>▪ *specify*	
**Adherence information of participant from last 3 months**		
How often did participant miss ART in the last months?	▪ *Every day*<br>▪ *More than 1/ week*<br>▪ *Once a week*<br>▪ *Once every two weeks*<br>▪ *Once a month*<br>▪ *Never*	*Categorical*
Did participant miss ART at two or more following days within the last month?**	▪ *Yes*<br>▪ *No*	*Binary*
**EAC information**		
EAC choice	▪ *Standard face-to-face EAC at the clinc*<br>▪ *EAC on phone with DAN*<br>▪ *ART Intake reminders*	*Categorical*
If EAC choice “ART intake reminder”	*If yes:*<br>▪ *Specify weekday*<br>▪ *Specify time*<br>▪ *Choose message:*<br>○ *Meds time (emoji for clock)*<br>○ *Nako ea lithlare (emoji for clock)*<br>○ *Recharge!*<br>○ *Healthy living!*<br>○ *Bophelo bo botle!*<br>○ *Me and good health!*<br>○ *Nna le bophelo bo botle!*<br>○ *Right time!*<br>○ *Nake e nepahetseng*	*Binary, free text and categorical*

***Recommented actions
1st VL >= 50, Prefeence based EAC and next VL[3 months after 1st VL]
2nd VL >= 50, Continue EAC, consult AAC
2nd VL >= 1000, Consult AAC for switch to effective regimen, consider GRT

15.4. Case report form 4: Pharmacy dispense and tracing list generated

**Variable**	***Categories***	***Format***
**Pharmacy dispense**		
ART number		
Date of next ART refill		*Predefined date format*
Number of days ART pills dispensed for		
If TPT given: Date of next TPT refill		*Predefined date format*

If TPT given: Start of TPT date		*Predefined date format*
If TB treatment given: Date of next TB treatment refill		*Predefined date format*
If TB treatment given: Start of TB treatment date		*Predefined date format*

**Tracing**		
ART number		
Participant name		*Displayed to the VRA*
Tracing result	▪ *Participant died*<br>▪ *Participant is hospitalized*<br>▪ *Participant gets ART from another clinic in Lesotho*<br>▪ *Participant gets ART from another clinic in South Africa*<br>▪ *Participant does not take ART anymore*<br>▪ *Participant stops taking part in VITAL*<br>▪ *No information found about the participant at all*	
If Tracing result "Participant died":	▪ *HIV-related*<br>▪ *TB-related*<br>▪ *Cancer*<br>▪ *Accident*<br>▪ *Suicide*<br>▪ *Other*	

15.5. Case report form 5: Data from District ART nurse

Purple background indicates variables provided for DAN and gray background refers to variables filled in by the DAN (offered for participants attending intervention clinics).

Variable	*Categories*	*Format*
**EAC with DAN**		
ART number		
Initals		
DOB / Sex		
Phone number		
Phone owner		
Last DAN contact		
Next DAN contact		
Number of call attempts		
Duration of call		
EAC session number		
Contact with DAN (reason + clinical reason)		
Next EAC session date		*date*
DAN contact comments	▪	*To be displayed on*

		health center nurse tablet
When was the last time you missed any medications?	▪ *Past week*<br>▪ *1-2 weeks ago*<br>▪ *3-4 weeks ago*<br>▪ *>4 weeks ago or never*	*Categorical*
Did you miss ART at two or more following days within the last month?**	▪ *Yes*<br>▪ *No*	*Categorical*
How often did you miss ART in the last months?**	▪ *Every day*<br>▪ *More than 1/ week*<br>▪ *Once a week*<br>▪ *Once every two weeks*<br>▪ *Once a month*<br>▪ *Never*	*Categorical*
If not "Never": What were the reasons for not taking ART?**	▪ *Forgetting*<br>▪ *Busy*<br>▪ *Change to daily routine*<br>▪ *Travelling/ being away from home*<br>▪ *Side effects/ fear of side effects*<br>▪ *Run out of pills*<br>▪ *Depressed/ alcohol/ Daha*<br>▪ *If other: specify (free text)*	

**Does not appear on the study tablet of nurse.

**Call-back from VITAL DAN**	
ART number	
Number of call attempts	
Duration of call	
Reason for call-back	▪ *Clinical question*<br>○ *Specify (text)*<br>▪ *Did not understand VL message*<br>▪ *Study withdrawal*<br>▪ *Phone number changed*<br>○ *New number*<br>▪ *Unclear / other*<br>○ *Specify (text)*

15.6. Additional documents

This protocol is valid together with the following documents:

- VITALstudy_ICF-Intervention_English_v1.0.docx
- VITALstudy_ICF-Intervention_Sesotho_v1.0.docx
- VITALstudy_ICF-Control_English_v1.0.docx
- VITALstudy_ICF-Control_Sesotho_v1.0.docx

- Appendix 1_VITAL suicide risk assessment SOP all staff_23.04.21.docx
- Appendix 2_VITAL suicide risk assessment SOP nurses v2.0_23.04.21.docx
- Appendix 3_VITAL qual substudy_ICF-intervention feedback_English_v1.0_2021.06.25.docx
- Appendix 4_VITAL qual substudy_ICF-intervention feedback_Sesotho_v1.0_2021.06.25.docx
- Appendix 5_VITAL qual substudy_ICF-mental health_English_v1.0_2021.06.25.docx
- Appendix 6_VITAL qual substudy_ICF-mental health_Sesotho_v1.0_2021.06.25.docx
- Appendix 7_VITAL Intervention feedback IDI guide_10.07.21.docx
- Appendix 8_VITAL Mental health IDI guide_10.07.21.docx
- Appendix 9_VITALstudy_MH_Questionnaires_v1.2.docx
- Appendix 10_VITALstudy_monitoring_plan_v1.2.docx
